# An information content principle explains regulatory patterns of gene expression across human tissues

**DOI:** 10.1038/s41467-026-71279-1

**Published:** 2026-04-11

**Authors:** Ruthie Golomb, Maayan Yoles, Simon Fishilevich, Bar Cohen, Sapir Savariego Peled, Dvir Dahary, David Gokhman, Yitzhak Pilpel

**Affiliations:** 1 Department of Molecular Genetics, Weizmann Institute of Science, Rehovot, Israel; 2 Department of Molecular Cell Biology, Weizmann Institute of Science, Rehovot, Israel

**Keywords:** Gene regulation, Evolutionary genetics, Gene regulatory networks

## Abstract

Gene expression ranges from broadly expressed to tissue-specific patterns, with many genes displaying intermediate specificity. Understanding how regulatory architecture scales with tissue specificity can reveal fundamental principles of genome regulation. By analyzing *cis*-regulatory element counts across human genes with varying tissue specificity, we identify a non-monotonic pattern: genes with intermediate specificity harbor the most regulatory elements, suggesting distinct regulatory strategies across the expression spectrum. We apply the Minimum Description Length principle from information theory, and maximum parsimony from phylogenetics, to quantify regulatory demand underlying expression patterns. This measure scales consistently with *cis*-regulatory element counts, transcription factors, microRNAs, and gene structure, and distinguishes switch-like regulation in selectively expressed genes from fine-tuning regulation in broadly expressed genes. Regulatory element abundance peaks in genes of intermediate evolutionary age. Regulatory architecture appears to scale with informational costs, suggesting that the genome operates as a decompression device, where regulation is dictated by minimally required complexity.

## Abstract (web summary)

Gene expression ranges from broad to tissue‑specific, but how regulation scales across this range was unclear. Here, the authors show that genes with intermediate specificity have the most regulatory elements and reveal informational principles shaping genomic regulatory architecture.

## Introduction

Gene expression patterns vary widely across human tissues, reflecting distinct functional demands across cell types. Some genes are ubiquitously expressed, performing essential housekeeping roles such as RNA processing, protein synthesis, and core metabolism^1,2^. Others exhibit tissue-specific expression associated with specialized functions like neuronal signaling, spermatogenesis, and sensory perception^1,3^. Yet, between these extremes lies a large class of genes with intermediate tissue specificity, expressed highly in some tissues, yet minimally or not at all in others. Intermediately expressed genes have received less systematic attention, and their biological roles remain less characterized. Notably, genome-wide profiling by Yanai et al. demonstrated that this ‘midrange’ class comprises a substantial fraction of genes and suggested that crucial functional information resides within them^4^. Despite their abundance, it is unclear whether the characteristics of intermediately expressed genes simply represent a midpoint between broadly and narrowly expressed genes, or whether they exhibit distinct, even exaggerated, features.

Patterns of gene expression are governed by multiple interconnected layers of regulation. Central to this regulatory architecture are *cis*-regulatory elements (CREs), which are sequences that regulate the expression levels of target genes, sometimes across large genomic distances^5–7^. CREs include enhancers, promoters, and silencers and play key roles in establishing tissue-specific expression patterns. Genome-wide studies have extensively identified CREs and mapped them to their target genes^8–11^. Transcription factors (TFs) bind to these CREs, coordinating temporal and spatial expression profiles^12–16^. The human genome encodes approximately 1600 TFs, with annotated target genes for many^17–19^. Regulation also occurs post-transcriptionally, involving factors such as microRNAs (miRNAs), that modulate mRNA stability and translation^20,21^. Over 2000 human miRNAs have been annotated, and genome-wide interaction maps are available^22–25^. Gene regulation is also encoded within gene structure itself, as the length of untranslated regions (UTRs), coding sequences (CDS), and introns influences regulatory potential. Longer segments can accommodate more *cis*-acting elements, facilitating complex transcriptional and post-transcriptional control^26–29^. For instance, during early development, maternally supplied mRNAs retain long 3′ UTRs that support extensive post-transcriptional control, whereas zygotic mRNAs possess extended promoters to support the complex transcriptional programs required for initiating cell fate decisions^30^.

This diversity in expression patterns and regulatory mechanisms raises a fundamental question: how does regulatory architecture scale with expression pattern? Several models are possible. Broadly expressed genes may require complex regulatory mechanisms to maintain expression across the body. Conversely, tissue-specific genes might require tight regulation to ensure repression outside their intended context. Genes with intermediate tissue specificity might require intermediate levels of regulation or alternatively, the most elaborate control to balance selective activation and repression. Or, regulatory information content might be unrelated to expression patterns altogether and instead shaped primarily by other evolutionary or functional constraints.

To address this question, we conducted a genome-wide analysis of how gene expression patterns relate to multiple layers of regulatory control. Classic tissue specificity measures summarize where a gene is expressed but do not typically consider how expression patterns relate to the biological relationships between tissues. However, tissue relationships are meaningful: a gene expressed in three closely related immune tissues likely requires less regulatory information than one expressed in three unrelated tissues. To incorporate this dimension, we developed an ontogeny-aware, parsimony-based measure that estimates the minimal number of regulatory transitions required to explain a gene’s expression pattern across the tissue hierarchy. This measure ultimately quantifies the expression complexity, or regulatory demand, of a gene.

We applied this framework to examine the relationship between multiple regulatory features and the measured expression complexity of genes. We further tested whether this relationship behaves differently in selectively expressed versus ubiquitous genes, assessing whether the regulatory features act as binary ‘switches’ or quantitative ‘knobs’. We extended this analysis to explore how these patterns are shaped by evolutionary gene age. Collectively, this approach offers a quantitative framework for understanding how gene expression patterns are encoded by regulatory architecture and shaped evolutionarily.

## Results

### Tissue specificity spans a broad distribution with a substantial intermediate class

To quantify tissue specificity, we used the tau index^4^, a widely adopted measure that integrates both the number of tissues in which a gene is expressed and the variability of its expression levels across tissues. The tau values range from 0 (ubiquitous even expression across all tissues) to 1 (strict tissue specificity). A comparative evaluation of nine tissue specificity metrics identified tau as the most robust^31^, and it is widely used, e.g., in the Human Protein Atlas (HPA)^32,33^. We calculated tau values for 18,234 protein-coding genes from HPA expression data, derived from both bulk RNA-seq covering 40 tissues and single-cell RNA-seq data aggregated by 81 cell types^34^. Consistent with previous findings by HPA^34^, tau values computed from bulk data closely mirrored those derived from the single-cell data (Spearman’s *ρ* = 0.88, *p* < 1 × 10⁻³⁰⁰; Supplementary Fig. 1A), indicating that tissue specificity is consistent across different biological resolutions. Additionally, tau correlated strongly with the Gini index (Spearman *ρ* = 0.98, *p* < 1 × 10⁻³⁰⁰; Supplementary Fig. 1B), another established measure of tissue specificity, further supporting its consistency across metrics.

**Fig. 1:**
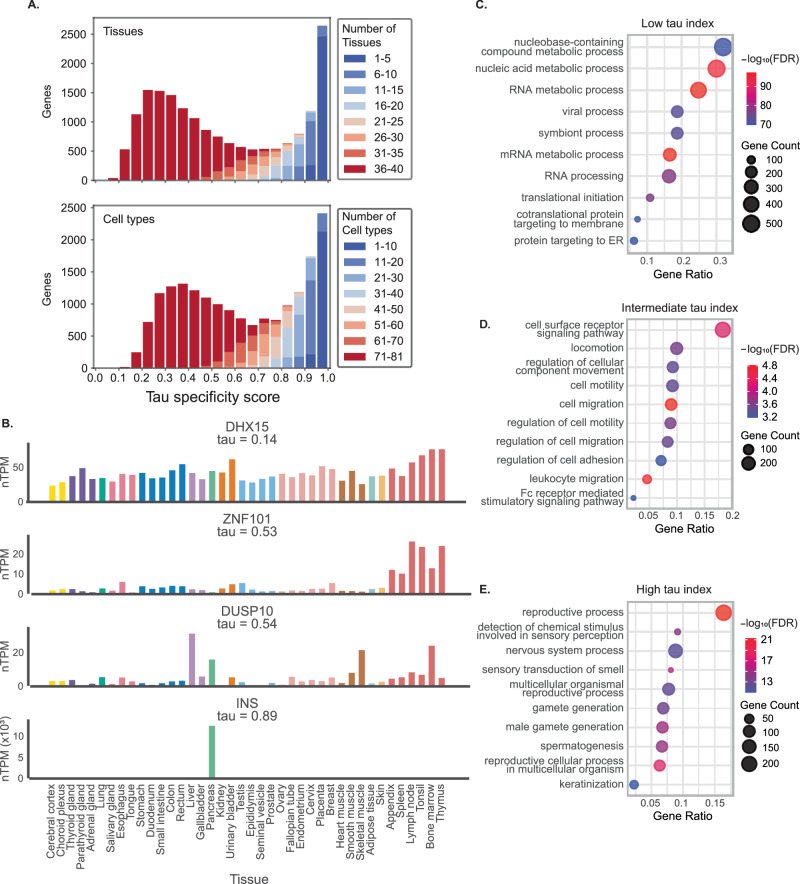
Tissue specificity of human protein-coding genes and associated functional enrichments. **A** Distribution of tau scores, calculated from bulk RNA-seq tissue samples (top) and single-cell RNA-seq data aggregated by cell type (bottom), for 18,324 human protein-coding genes. Tau ranges from 0 (ubiquitous, even expression) to 1 (strict tissue specificity). Bars are colored by the number of tissues or cell types in which each gene is expressed, illustrating the relationship between tau values and expression breadth. **B** Bar plots showing tissue expression profiles of representative genes spanning the tau spectrum. Tissues are color-coded by groups with shared functional characteristics, as defined by the HPA. Y-axis values indicate normalized gene expression (normalized transcripts per million, nTPM) across 40 tissues. **C–E** Bubble plots showing ranked Gene Ontology (GO) term enrichment based on tau values: **C** Genes broadly expressed (ranked from lowest to highest tau). **D** Genes with intermediate tissue specificity (ranked by proximity to tau = 0.5) **E** Highly tissue-specific genes (ranked from highest to lowest tau). The x-axis represents the gene ratio, bubble size indicates gene count, and bubble color shows the -log₁₀ adjusted *p*-value.

The distribution of tau values exhibited a broad dynamic range, with peaks at both the low and high ends (Fig.1A). While most genes exhibited either broad expression (low tau) or strict tissue specificity (high tau), a substantial subset fell in the intermediate range. By coloring the bar plot according to the number of tissues or cell types a gene is expressed in, we found that tau is strongly, but not exclusively, determined by expression breadth. This is further illustrated by plotting tau against the number of tissues (Supplementary Fig. 1C), revealing that broadly expressed genes can still span a wide range of tau values. Notably, genes with tau values up to approximately 0.5 are expressed in essentially all tissues, indicating that roughly half of the tau range reflects expression level variation rather than number of tissues. Values near zero indicate uniform expression across tissues, whereas values approaching 0.5 reflect increasing unevenness; beyond this region, higher tau values correspond to progressively restricted expression, ultimately limited to one or a few tissues.

To illustrate the range of expression profiles captured by the tau index, we display representative genes spanning the spectrum of tissue specificity. *DHX15*, an RNA helicase, exhibits uniform expression across all tissues and a low tau value (0.14), consistent with its housekeeping function. At the opposite extreme, the gene encoding insulin (*INS*) is predominantly expressed in the pancreas and shows a high tau value (0.89), exemplifying tissue specificity. Intermediate specificity is more heterogeneous, as demonstrated by two genes with tau values of ~ 0.5 (Fig.1B). *ZNF101*, a zinc-finger protein involved in transcription regulation, is expressed at low levels across most tissues but shows elevated expression in immune-associated tissues such as lymph nodes, tonsils, and thymus. In contrast, *DUSP10*, a MAP kinase phosphatase, is also broadly expressed at low levels, but exhibits relatively high expression in a functionally diverse set of tissues including liver, pancreas, skeletal muscle, and bone marrow. While both genes share similar tau values, their expression patterns differ in the degree of tissue-related coherence. These examples highlight the diversity of expression patterns across the tau spectrum, with intermediate values capturing both tissue-coherent and functionally dispersed expression profiles.

To characterize biological processes associated with these gene-expression patterns, we performed ranked enrichment analyses using GOrilla^35^, ranking genes by tau values. Genes with low tau values were enriched for fundamental cellular functions, such as RNA metabolism and translation initiation, consistent with their housekeeping roles (Fig. 1C). Genes with high tau values showed significant enrichment for specialized processes, including sensory detection (olfaction), reproduction (spermatogenesis), and keratinization, matching tissue-specific functions (Fig. 1E). Genes with intermediate tau values closest to 0.5 were enriched for processes such as cell-migration and immune-related processes (Fig. 1D); albeit with lower statistical significance. This lower significance suggests that intermediate-specificity genes represent a biological phenomenon, yet they comprise a more functionally heterogeneous group.

These results collectively highlight a wide continuum of tissue specificity, motivating the question of how regulatory complexity scales across this spectrum.

### CRE abundance peaks at intermediate levels of tissue specificity

How might regulatory architecture relate to tissue specificity? To reveal this relationship, we first examined the distribution of regulatory elements across the tissue specificity spectrum. We began with CREs because they constitute the primary regulatory elements controlling spatiotemporal and cell-type-specific gene expression in metazoan genomes and are extensively annotated across diverse tissues. The number of CREs linked to a gene can serve as a genome-wide, quantitative proxy for the diversity of *cis*-regulatory inputs needed to implement its expression pattern.

Candidate CRE (cCRE) annotations and predicted gene-cCRE associations were obtained from the GeneHancer database^10^, which integrates data from multiple sources and assigns confidence scores to cCREs and their target links. We used both the full dataset (419,020 cCREs) and the more stringent “Double Elite” subset (122,815 cCREs), which includes only high-confidence cCRE-gene pairs. The main figures show results based on the full set, while Supplementary Fig. 2 presents the Double Elite analyses, with results consistent across both thresholds (see Methods).

**Fig. 2:**
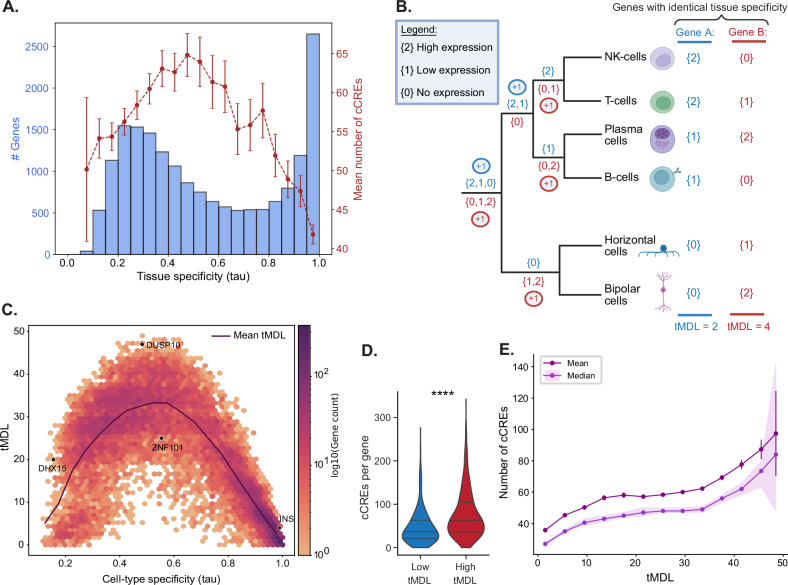
tMDL captures regulatory information content beyond tissue specificity. **A** Histogram of tau scores calculated from tissue-level bulk RNA-seq data (blue bars, left y-axis), with overlaid line plot (red dashed line, right y-axis) indicating the mean number of candidate *cis*-regulatory elements (cCREs) linked to genes within each tau bin, with error bars representing the 90% confidence interval (CI) of the mean. **B** Schematic illustrating how the tree-aware minimum description length (tMDL) is calculated using Fitch’s parsimony algorithm. Two hypothetical genes (Gene A, blue; Gene B, red) have identical tau values but differ in their expression patterns across a hierarchical cell-type tree. Leaf nodes show discretized expression levels: {0} for no expression, {1} for low expression, and {2} for high expression. Internal nodes are labeled with the possible expression states consistent with the expression patterns of their descendant cell types. Regulatory transitions are indicated along branches as “+1,” with each +1 representing one required expression-level change, and the total number accumulates along the path. The total number of transitions across the tree defines the tMDL score, shown at the bottom for each gene. Created in BioRender. Dahan, O. (2026) https://BioRender.com/toyrkho. **C** Hexbin plot showing the relationship between cell-type specificity (tau) and tMDL across human protein-coding genes, calculated from single-cell RNA-seq data aggregated by cell type. Each hexagon represents the genes within that region of the plot, with color indicating local density. The example genes from Fig. 1B are marked with black dots. **D** Violin plot comparing the number of cCREs between low- and high-tMDL genes (bottom and top 20%) within the intermediate tau gene set. Low-tMDL: *n* = 1,185 genes; high-tMDL: *n* = 1215 genes. Two-sided Mann-Whitney U test: *p* = 2.21 × 10⁻⁴⁷, *r* = 0.30. **E** Line plot showing the mean (purple) and median (violet) number of cCREs per gene, computed across genes ranked by tMDL and grouped into non-overlapping fixed-interval bins. Error bars and shaded areas represent 90% CIs for mean and median, respectively. All analyses in this figure were performed on *n* = 18,234 human protein-coding genes.

We grouped genes into tissue specificity (tau) bins based on bulk RNA-seq expression across tissues and in each bin, calculated the mean number of linked cCREs per gene. We observed an inverted U-shaped relationship. Genes with intermediate tissue specificity exhibited the highest mean cCRE counts, while both broadly expressed and highly tissue-specific genes have fewer cCREs (Fig. 2A). This pattern indicates that genes with intermediate tissue specificity require the most diverse cCRE inputs to achieve selective and context-dependent expression. Motivated by this finding, we sought a theoretical framework that could account for this non-monotonic relationship.

### Minimum Description Length (MDL) as a framework for understanding gene regulatory architecture

The observation that cCRE abundance peaks in genes with intermediate tissue specificity suggests a non-monotonic relationship between tissue specificity and regulatory information content per gene. To explain this pattern, we turned to a principle from information theory called the Minimum Description Length (MDL). MDL states that the best representation of a system is the one that minimizes the amount of information required to describe it^36,37^. In essence, this frames gene regulation as a problem of information compression: how to encode a complex expression pattern concisely. Applied to gene regulation, this principle predicts that genes with simple expression patterns should require minimal regulatory architecture, while those with more complex patterns should require more elaborate control mechanisms.

For example, housekeeping genes may follow the simple rule “express in all tissues,” and tissue-specific genes follow “express only in tissue X.” Both represent concise regulatory instructions. In contrast, a gene that must be expressed in a subset of tissues, for instance “express in tissues A, D, and F but not or lowly in B, C, or E”, generally requires a more complex regulatory program. According to the MDL principle, regulatory information content should therefore follow an inverted U-shaped relationship with tissue specificity. Genes at either extreme with a short MDL describing their expression pattern should require simpler regulatory mechanisms, with lower information content in their regulatory program than those with intermediate tissue specificity.

### A maximum parsimony-based measure estimates MDL

While the tau index effectively captures expression distribution across tissues, it is only a proxy for estimating the regulatory information content predicted by MDL. The tau index does not account for the hierarchical or ontogenetic relationships between tissues. For example, both *ZNF101* and *DUSP10* have intermediate tau values (Fig.1B), yet their expression profiles differ markedly in their tissue coherence. *ZNF101* is highly expressed in functionally related immune tissues such as lymph nodes, tonsils, and thymus, suggesting the potential for a shared regulatory program. In contrast, *DUSP10* shows elevated expression in a more dispersed set of tissues which are not related in expression programs. Although tau treats these genes as equivalent in specificity, their regulatory demands likely differ, with *ZNF101* requiring fewer developmental switches and thus a shorter MDL, while *DUSP10* may necessitate multiple independent regulatory instructions. The relationships between all tissues or cell types in the body can be depicted by a hierarchical tree of relatedness, approximated by similarity in expression programs^38^ (though exceptions can arise from convergent expression among developmentally distinct tissues). This underlying organization of tissues underscores the need for a tree-aware measure of regulatory demand that captures expression distribution in the context of this hierarchical structure.

Another computational biology domain that captures similarities based on hierarchical trees is phylogenetics. In evolutionary trees, maximum parsimony algorithms are used to infer the traits of ancestral species and to estimate the number of changes (e.g., nucleotide substitutions) along each branch. We recognized that the phylogenetic and ontogenetic problems are related, as both involve inferring the number of events (mutations in evolution or gene expression changes during development, respectively) that occur along each branch of a tree from a common node. This conceptual connection between phylogenetic methods and ontogeny has been explored in previous work that applied phylogenetic tools to expression-based cell type trees to gain developmental and functional insights^39–42^.

Here we accordingly adapted a maximum parsimony approach from phylogenetics to quantify a gene’s regulatory demands. By analogy to phylogenetics, we constructed a hierarchical tree of cell type relationships using the genome-wide gene expression profiles and hierarchical clustering (see Methods), as in prior studies^38,41,43^. We used cell type-level data to build the tree, as single-cell-derived profiles provide a cleaner estimate of biological relationships than bulk tissue data, which can be confounded by compositional bias. We then adapted Fitch’s algorithm^44^, originally developed to infer the minimum number of mutations required to explain sequence variation across a phylogenetic tree. In our framework, this algorithm is used to assess the minimal number of expression-level transitions needed to produce a given expression pattern, where a transition is defined as a change in discretized expression bin. The resulting score, referred to as the tree-aware MDL (tMDL), reflects the estimated minimal regulatory load required for a gene’s expression program while accounting for the relatedness of cell types (see Fig. 2B, and Supplementary Fig. 4 for method’s robustness analyses). To ensure that our results do not depend on the specific cell-type hierarchy used, we recomputed tMDL using an independent chromatin accessibility-based cell-type tree derived from single-cell ATAC-seq data^45^. tMDL values were highly correlated across trees (Pearson *r* = 0.95, *p* < 1 × 10⁻³⁰⁰; Spearman *ρ* = 0.95, *p* < 1 × 10⁻³⁰⁰; Supplementary Fig. 3).

Higher tMDL scores indicate that more independent regulatory changes are required to produce the observed expression profile. Genes with high tMDL scores are generally expressed in several cell types that are not clustered together on the hierarchical tree. For instance, *ZNF101* and *DUSP10*, that both show intermediate tau values, differ markedly in their tMDL. *ZNF101* has a tMDL of 17, among the lowest observed at this tau level (Fig. 2C), because its high expression is restricted to closely related immune cell types. In contrast, *DUSP10* has a tMDL of 45, one of the highest for genes with similar tau, due to its high expression across a set of distantly related cell types. This illustrates how tMDL distinguishes between expression profiles that appear similar in breadth and tau but differ in the complexity of cell-type-specific regulation.

Figure 2C shows, as predicted by the MDL principle, that genes with intermediate cell-type specificity (tau ≈ 0.5) showed the highest tMDL, indicating they require the most regulatory information content. Notably, genes in this intermediate range also exhibit considerable variation in their tMDL scores, suggesting diverse levels of regulatory demand among genes with similar expression breadth and cell-type specificity. To examine this variation more directly, we focused on genes with intermediate tissue specificity and compared those in the bottom versus top 20% of tMDL values using a rank-based stratification (see Methods). Despite having comparable tau values, intermediate-tau genes with high tMDL harbor significantly more linked cCREs than those with low tMDL (two-sided Mann-Whitney U test, *p* = 2.2 × 10^−47^, *r* = 0.30; Fig. 2D), indicating that regulatory architecture varies substantially even among genes with similar expression breadth. This distinction is not specific to cCREs and is also observed across additional regulatory layers (Supplementary Fig. 5). These findings highlight how combining tau with tMDL provides a richer, more comprehensive view of regulatory architecture.

Genes with high tau (cell-type-specific) showed low tMDL, near one, reflecting the simple logic of being “off” in most cell types and “on” in only one or a few, often requiring just a single regulatory transition. Genes with low tau (broadly expressed) generally had higher tMDLs than cell-type-specific genes, but lower than many intermediate-tau genes. As tau approaches zero, tMDLs tend to decrease, consistent with the expectation that uniform expression requires fewer regulatory transitions. Nonetheless, broadly expressed genes also showed tMDL variability, reflecting differences in how consistent expression levels are maintained across cell types.

### Diverse regulatory features align with MDL framework

We next tested whether various layers of gene regulation scale with complexity as predicted by MDL. We examined four regulatory features: cCREs, TFs, miRNAs, and lengths of various gene structural elements, assessing their relationship with regulatory demand (tMDL) and tissue specificity (tau).

### cCREs

As seen in Fig. 2A, cCRE count per gene peaks among genes with intermediate tissue specificity. We asked whether cCRE count also scales with tMDL, thereby reinforcing its value as a proxy for regulatory information content. To test this, genes ranked by tMDL were grouped into non-overlapping fixed-interval bins, and the mean and median cCRE counts were calculated within each bin. This analysis revealed a clear trend: genes with higher tMDL scores tended to have more cCREs (Fig. 2E). We conclude that genes with more transitions in expression state within the tree require more inputs from cCREs.

To assess robustness to regulatory annotation and species, we repeated the cCRE analysis using a mouse single-cell chromatin accessibility atlas^46^ (Supplementary Fig. 6). The increase in cCRE number with tMDL was preserved, and the inverted U-shaped relationship with tissue specificity was also retained, though with some variation among broadly expressed genes. This supports the robustness of the observed cCRE regulatory patterns.

### Transcription factors

Given the central role of TFs in modulating gene expression, we next examined how TF targeting relates to tissue specificity and regulatory information content. TF-target gene relationships were obtained from the hTFtarget database, which compiles experimentally supported TF binding events from ChIP-seq datasets and motif predictions, assigning targets when binding occurs in promoter regions^47^.

**Fig. 3:**
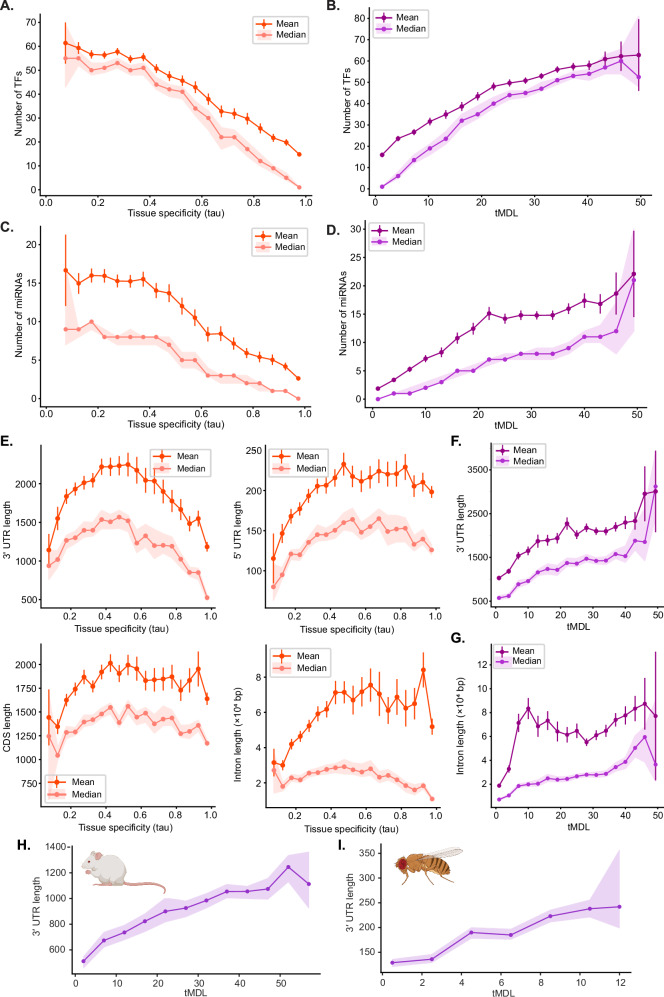
Transcriptional and structural regulatory features scale with regulatory demand (tMDL). **A**, **B** Mean and median transcription factors (TF) counts per gene were computed across ranked tau (**A**) or tree-aware minimum description length (tMDL) bins (**B**). **C**, **D** Mean and median miRNA counts per gene across bins of tau (**C**) and tMDL (**D**). **E** Gene structure features across tau: 3′ UTR, 5′ UTR, coding sequence (CDS), and intron lengths. **F**, **G** 3′ UTR length (**F**) and intron length (**G**) across bins of tMDL. Panels A-G were performed on 18,234 human protein-coding genes; the exact number included in each panel varies slightly depending on feature availability. Complete gene-level annotations are provided in Supplementary Data 1. **H**, **I** Median 3′ UTR length per gene across ranked tMDL bins in mouse (14,486 genes; H) and *Drosophila* (9,686 genes; I). Mouse analyses were performed using single-cell RNA-seq data aggregated by cell type^85^, whereas *Drosophila* analyses used tissue-level bulk RNA-seq data^86^. Mouse and Drosophila icons created in BioRender. Dahan, O. (2026) https://BioRender.com/m1g8zl2. In all panels, dark lines with error bars (90% CI) represent mean values and light lines with shaded area (90% CI) represent median values. Orange hues correspond to tau-based plots, and purple hues to tMDL-based plots.

While cCRE count peaked among genes with intermediate tissue specificity, TF targeting followed a distinct pattern. Broadly expressed genes were targeted by the most TFs (Fig. 3A). Notably, TF counts were high and relatively constant for genes with tau values below ~0.4, and declined progressively as tau increased beyond this point. This suggests that TF number primarily scales with expression breadth, and once genes are broadly expressed, variation in expression levels is not accompanied by further increases in TF count. This trend differs from the cCRE pattern, suggesting that broadly expressed genes may require a more extensive *trans*-regulatory network to ensure robust and high expression across diverse cellular environments^48^. Since different tissues deploy distinct TF repertoires, genes that must be expressed broadly are likely compatible with many TF environments, resulting in a larger set of observed TF-target links. Genes with restricted expression breadth rely on more limited TF input, requiring activation in only a narrow set of conditions or cell types. We note that TFs can act as activators, repressors, or possess dual roles depending on cellular context and target gene^49–51^.

Despite these differences regarding tissue specificity, we observed a strong positive trend between TF number and tMDL (Fig. 3B). Genes with more complex expression patterns, as captured by higher tMDLs, tended to be targeted by more TFs. Although TF number declines steadily with increasing tissue specificity, its robust progressive increase with tMDL suggests that TF targeting still reflects overall regulatory burden, capturing aspects of regulatory complexity that go beyond tissue specificity alone. These findings show that, like cCRE count, TF targeting scales with regulatory demand as captured by tMDL.

### microRNAs

We next turned to post-transcriptional regulation, focusing on miRNAs. Experimentally detected miRNA-target interactions were obtained from TarBase, a comprehensive, manually curated database that catalogs over 2 million unique miRNA-gene pairs supported by diverse experimental methods^52^. Similar to TF targeting, we found that broadly expressed genes harbored the highest number of targeting miRNAs, while tissue-specific genes contained the fewest (Fig. 3C). When compared with tMDL, the number of targeting miRNAs showed a strong positive slope (Fig. 3D), meaning genes with more complex expression patterns tended to be targeted by a greater number of miRNAs. This trend demonstrates that genes under higher regulatory demand also engage more extensively with post-transcriptional mechanisms, aligning with the MDL framework’s prediction. It closely parallels the trend for TFs, which have been shown to co-regulate shared targets with miRNAs and even regulate each other in integrated network motifs^53^. Despite these similarities, TF and miRNA counts showed a low correlation (Supplementary Fig. 7), indicating their similar scaling with tMDL is not due to a dependency between them. The progressive increase in both miRNA and TF targeting with tMDL suggests that both transcriptional and post-transcriptional trans-regulatory inputs scale with overall regulatory demand, even when not directly reflected by tissue specificity.

### Gene structure

We next examined how gene structural features (i.e., CDS, intron, and UTR lengths) relate to tissue specificity and tMDL. Prior studies have linked gene structure to regulatory potential and mRNA stability^26,27,29,30,54,55^. Additionally, broadly expressed housekeeping genes are known to be typically shorter; an observation thought to reflect selection against the energetic cost of long transcripts^56^. Under the MDL framework, tissue-specific genes are also expected to be relatively short, since regulatory information may be embedded within the gene’s structure too. In contrast, genes with more complex expression patterns may encode additional regulatory instructions within their structure, resulting in increased length.

Consistent with this, genes with intermediate tissue specificity showed the longest CDS, introns, and UTRs (Fig. 3E). While some features displayed a more asymmetrical inverted U-shape, particularly with broadly expressed genes often being the shortest, the 3′ UTR length showed the most pronounced trend of peaking among genes with intermediate specificity.

We next asked whether these structural features correlate with tMDL. Both 3′ UTR and intron lengths increased with tMDL, indicating that genes requiring more regulatory state transitions tend to have longer non-coding regions (Fig. 3F, G). This aligns with their known regulatory roles: 3′ UTRs often serve as hubs for post-transcriptional control, including miRNAs, RNA-binding protein interactions, and other regulatory elements, while introns can harbor CREs, miRNA binding sites, and additional regulatory elements^57–59^. Among all features, 3′ UTR length showed the strongest relationship with both tau and tMDL, highlighting its regulatory importance. Although longer 3′ UTRs offer more space for miRNA binding, the correlation between miRNA count and 3′ UTR length was only moderate (Supplementary Fig. 7), indicating that UTR length captures additional regulatory complexity. In contrast, CDS and 5′ UTR lengths showed a lower correlation with tMDL, consistent with the 5′ UTR’s primary role in translation initiation rather than expression regulation^60^, and with prior observations that 5′ UTR length does not contribute to tissue specificity^61^.

Finally, we asked whether the observed scaling between tMDL and regulatory complexity is specific to humans or reflects a more general principle across species. We extended this analysis to mouse and to the more distantly related *Drosophila* (See methods; Supplementary Fig. 8). In both species, the characteristic distribution of tau values, with peaks corresponding to broadly expressed and tissue-specific genes, was preserved (Supplementary Fig. 8A, F). Importantly, the core relationship between regulatory demand and regulatory complexity also remained: genes with higher tMDL exhibited longer 3′ UTRs, indicating increased regulatory capacity as expression patterns become more complex along the corresponding cellular or tissue hierarchy (Fig. 3H, I). A similar positive scaling was observed for the number of TFs in both species, using the TFlink database^62^ (Supplementary Fig. 8E, I). While regulatory annotations of comparable breadth to those available for humans are more limited in other model organisms, these findings demonstrate that the coupling between expression-pattern complexity and regulatory architecture is conserved across substantial evolutionary distances, supporting the generality of the MDL-based framework.

### Quantitative framework distinguishes knob-like and switch-like regulatory mechanisms across gene expression regimes

Genes with tau values below ~0.5 are broadly expressed across all tissues and thus show variation in their relative expression levels rather than presence or absence in each tissue. These genes are expected to require fine-tuned, quantitative control, which we refer to here as “knob-like” regulation. In contrast, genes with higher tau values are expressed in a more restricted set of tissues and thus involve a greater reliance on selective activation or repression, suggestive of “switch-like” regulation. In this context, “knob-like” and “switch-like” regulation are used as conceptual descriptors of expected regulatory behavior associated with broad versus restricted expression, reflecting a parsimonious regulatory logic: genes active across all cell types are most efficiently controlled through quantitative tuning, whereas genes restricted to few contexts are largely regulated through discrete activation or repression.

**Fig. 4:**
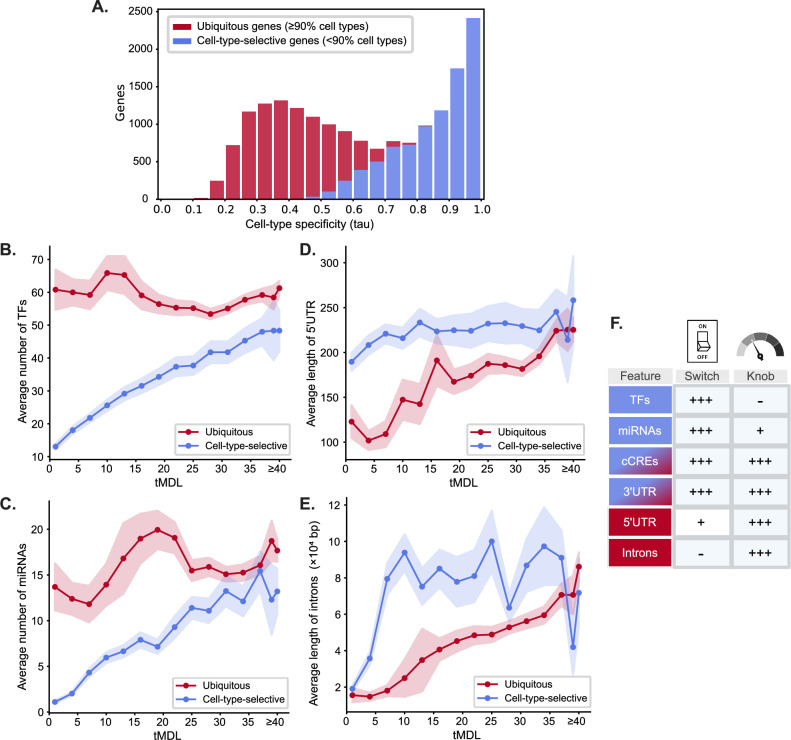
Distinct regulatory features scale with tMDL in cell-typeselective versus ubiquitous genes. **A** Histogram showing the distribution of cell-type specificity tau values, highlighting the two expression regimes used in this analysis: ubiquitous genes ( ≥ 90% of cell types, *n* = 8830 genes; red) and cell-typeselective genes (expressed in < 90% of cell types, *n* = 9404 genes; blue). **B–E** Mean feature values were calculated across fixed-interval bins along the ranked tree-aware minimum description length (tMDL) axis, separately for ubiquitous (red) and cell-type selective (blue) genes. Shaded areas represent 90% confidence interval. To avoid sparse bins, all genes with tMDL ≥ 40 were grouped into a final bin. **B** Number of transcription factors (TFs) per gene. **C** Number of miRNAs per gene. **D** 5′ UTR length. **E** Intron length. **F** Table summarizing regulatory features according to their association with tMDL in tissue-selective versus broadly expressed genes, representing switch-like and knob-like regulatory regimes, respectively. Symbols indicate the strength of the observed trend (based on linear regression slope): ‘+++’ = strong, ‘++’ = moderate, ‘+’ = weak, ‘–’ = none or negligible. Created in BioRender. Dahan, O. (2026) https://BioRender.com/r8lzd9o.

To examine how regulatory mechanisms differ across these expression regimes, we divided genes into two groups (Fig. 4A): those expressed in ≥ 90% of cell types (ubiquitous) and those expressed in fewer than 90% (cell-type-selective). The conclusions drawn from this stratification are not sensitive to the specific threshold used to define these groups; analyses using alternative cutoffs yielded consistent results (Supplementary Fig. 9). We then asked how regulatory features and tMDL scaled within each group, to assess whether different regulatory strategies support distinct expression regimes.

CRE abundance showed similarly robust correlations with tMDL in both broadly and selectively expressed genes (Supplementary Fig. 10), highlighting their universal importance across regulatory strategies, both knob-like and switch-like. However, TF targeting demonstrated strikingly different dynamics in the knob and switch regulated genes (Fig. 4B). In cell-type-selective genes, TF numbers increased greatly with rising tMDL, consistent with their important roles in discrete activation or repression events across specific tissues. Conversely, broadly expressed genes displayed high and relatively stable TF targeting across tMDL. This finding suggests that broadly expressed genes utilize a largely constant set of TFs, indicating that additional fine-tuning regulatory demands in these genes likely arise through alternative mechanisms rather than changes in TF number, potentially involving differential TF activity, co-factor interactions, CREs, or other regulatory inputs.

miRNA regulation patterns paralleled those observed for TFs (Fig. 4C). Cell-type-selective genes exhibited a strong positive relationship between miRNA count and tMDL, reinforcing miRNAs’ critical role as regulatory switches. Broadly expressed genes showed moderately increased miRNA targeting with rising tMDL, though this relationship was less steep. Thus, while miRNAs are broadly employed, they scale particularly strongly with regulatory complexity in cell-type-selective genes, highlighting their dominant role as switch-like regulators.

Gene structural features revealed additional complexity. Lengths of 3′ UTR regions increased consistently with tMDL in both expression regimes, highlighting their universal regulatory role (Supplementary Fig. 10). However, introns and 5′ UTR lengths exhibited regime-specific behavior: both increased significantly with tMDL in broadly expressed genes, but showed weaker trends in cell-type-selective genes (Fig. 4D, E), indicating a specialized role in fine-tuning regulatory complexity. This suggests that introns and 5′ UTRs contribute to fine-tuning in broadly expressed genes, but are not a major component of switch-like regulation in tissue-selective genes. As gene length is known to be constrained by energetic cost, particularly in highly expressed genes, the scaling of intron and 5′ UTR length with tMDL in broadly expressed genes may reflect selective pressure to maintain compactness except where additional regulatory content is required.

Together, these findings support a model in which distinct regulatory strategies underlie broadly versus selectively expressed genes. While some features, such as CREs and 3′ UTRs, scale with regulatory complexity across both regimes, others show regime-specific patterns. These distinctions are summarized in Fig. 4F, which classifies each regulatory feature according to its predominant role in switch-like versus knob-like regulation based on our observed patterns.

### Chromosome X as a potential case of chromosome-level MDL compression

**Fig. 5:**
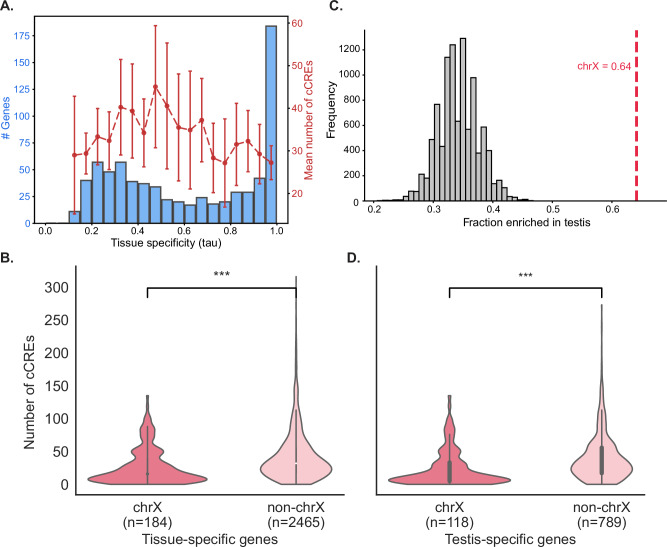
Tissue-specific genes on chrX have reduced cCRE counts and are enriched for testis-specific expression. **A** Histogram showing the number of genes on chrX across bins of tissue specificity (tau) (*n* = 730 genes). Overlaid in red is the mean number of candidate *cis*-regulatory elements (cCREs) per gene in each tau bin. Error bars represent 90% confidence interval of the mean. **B** Violin plot comparing the number of cCREs per gene in tissue-specific genes on chrX (tau ≥ 0.95; *n* = 184 genes, dark pink) versus tissue-specific genes on the rest of the chromosomes (*n* = 2465 genes; light pink), showing significantly lower cCRE counts on chrX (one-sided Mann-Whitney U test, *p* = 2.18 × 10⁻¹², *r* = 0.135). Violin plots show the distribution of values; the inner box indicates the interquartile range and the center indicates the median. **C** Null distribution showing the fraction of testis-specific genes (defined by dominant tissue of highest normalized transcripts per million (nTPM) and tau ≥ 0.95) across 10,000 randomly sampled gene sets of equal size (n = 184 genes) drawn from all tissue-specific genes (tau ≥ 0.95). The observed chrX proportion (118/184 = 0.64) is shown as a red dashed line. Significance was assessed using a one-sided permutation test; none of the 10,000 random gene sets reached or exceeded the observed value (empirical *p* < 1 × 10⁻⁴). **D** Violin plot comparing the number of cCREs for testis-specific genes on chrX (*n* = 118 genes, dark pink) versus testis-specific genes on the rest of the chromosomes (*n* = 789 genes, light pink), showing significantly lower cCRE counts on chrX (one-sided Mann-Whitney U test, *p* = 2.20 × 10⁻¹⁰, *r* = 0.207). Violin plots show the distribution of values; the inner box indicates the interquartile range and the center indicates the median.

Thus far, our analyses have examined the regulatory demands of individual genes across the genome. However, we observed that these principles may also extend to higher-order genomic organization. In agreement with previous studies, we found that genes located on the X chromosome (chrX) exhibit a significant bias toward tissue-specific expression^63,64^. Specifically, 25.3% of chrX genes exhibited high tissue specificity (tau ≥ 0.95), compared to 14.5% genome-wide (Fig. 5A).

Focusing on these tissue-specific chrX genes, we found that they are associated with a significantly lower number of cCREs compared to all tissue-specific genes across the genome (Fig. 5B). This indicates that the regulatory demand for tissue-specific genes on chrX is lower than expected, even given their restricted expression patterns.

To understand this further, we asked whether these genes are enriched for specific tissues. As previously observed^65–67^, we found a strong enrichment for testis-specific expression: approximately 65% of the tissue-specific genes on chrX are testis-specific. To evaluate whether this enrichment exceeded expectations, we generated 10,000 random gene sets matched for tau distribution. These typically included only 30–40% testis-specific genes, confirming a significant overrepresentation of testis-specific genes on chrX (Fig. 5C). Because the majority of chrX tissue-specific genes are testis-specific, we asked whether low cCRE counts are a general feature of testis-specific expression. However, testis-specific genes on chrX had significantly fewer cCREs than those on other chromosomes, suggesting a unique regulatory pattern on chrX (Fig. 5D).

These findings reveal that chrX harbors a disproportionately high number of testis-specific genes with lower-than-expected CRE counts. This pattern supports that MDL principles may apply not only to individual gene expression patterns but also to higher-order genomic organization. From an MDL perspective, when many nearby genes share a simple expression rule, such as “express in testis”, the collective regulatory burden can be reduced, minimizing the description length required. Such compression at the chromosome level could reflect a broader organizational principle, potentially shaping how regulatory programs are spatially arranged across the genome. While this observation aligns with the MDL framework, alternative explanations for the low cCRE count on chrX are possible and warrant further investigation.

### Tissue specificity and regulatory information content vary across evolutionary age

Gene expression and regulation evolve over time, with newly emerged genes integrating into existing networks while older genes are maintained, or lost, across species. Comparative studies have shown that older genes often possess richer regulatory landscapes, including more alternative splice forms and TF binding sites than recently evolved genes^68–70^. However, because gene age is closely tied to tissue specificity (older genes are typically broadly expressed, younger genes more tissue-specific^71,72^), this relationship must be considered when evaluating age effects on regulatory architecture. Accordingly, we examined how evolutionary age relates to tissue specificity, regulatory features, and tMDL.

**Fig. 6:**
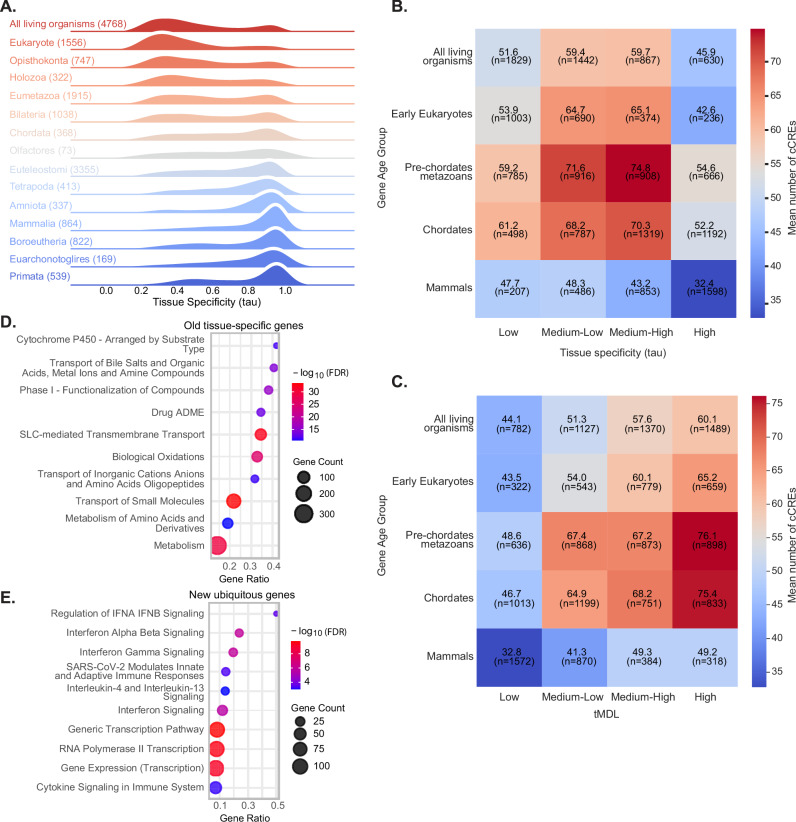
Tissue specificity and regulatory architecture vary across evolutionary gene age. **A** Smoothed density plots showing the distribution of tau values across evolutionary age groups. Genes are stratified by phylostrata, with the evolutionary age of each group indicated on the y-axis. Colors are used to distinguish age groups but carry no additional meaning. **B** Heatmaps displaying the mean number of candidate *cis*-regulatory elements (cCREs) for gene groups stratified by both evolutionary age (y-axis) and tissue specificity (x-axis, binned by tau values). **C** Heatmaps displaying the mean number of cCREs for gene groups stratified by both evolutionary age (y-axis) and tree-aware minimum description length (tMDL) (x-axis). **D**, **E** Bubble plots showing the top 10 significantly enriched Reactome pathways for (**D**) genes with high tissue specificity (tau > 0.8) and ancient evolutionary origin (phylostrata 1–4), *n* = 1145 genes, and **E** genes with low tissue specificity (tau < 0.4) and recent evolutionary origin (phylostrata 12–19), *n* = 1465 genes. The x-axis represents the gene ratio, bubble size indicates gene count, and bubble color shows the –log₁₀ adjusted *p*-value.

Gene ages were defined according to Litman & Stein^73^, who assigned each human gene to one of 19 phylostrata based on the most distantly related species in which an ortholog is detected, ranging from genes shared by all living organisms (phylostratum 1) to primate-specific genes (phylostratum 19). Analysis of tissue specificity across evolutionary age groups revealed a gradual, monotonic pattern (Fig. 6A). Ancient genes are overwhelmingly broadly expressed, genes of intermediate evolutionary age show intermediate tissue specificity, and recently evolved genes are highly tissue-specific. This pattern agrees with previous reports^71,72^.

Building on this, we next asked whether regulatory architecture also changes systematically with gene age, focusing on cCRE abundance across evolutionary groups. To control for the confounding effect of tissue specificity, we divided genes into four tau bins (low, medium-low, medium-high, high specificity) and five evolutionary age categories grouped from the original phylostrata classifications (“All living species”, “Early Eukaryotes”, “Pre-chordate metazoans”, “Chordates”, “Mammals”).

This analysis revealed a consistent age-dependent pattern in cCRE abundance. Across all tau levels, genes of intermediate evolutionary age consistently harbor more cCREs than both older and younger genes (Fig. 6B). This persistence within matched tissue-specificity groups shows that variation in regulatory information content is not simply a byproduct of tau, but is additionally structured by evolutionary age. Within each age group, except for the most recent, cCRE count was highest among genes with medium-low or medium-high tissue specificity, consistent with our previous results.

In addition to stratifying genes by tissue specificity (tau), we next asked how regulatory architecture varies with evolutionary age when genes are grouped by expression-pattern complexity (tMDL) (Fig. 6C). Across all evolutionary age groups, mean cCRE abundance increases with tMDL, indicating that the relationship between expression-pattern complexity and regulatory content captured by the tMDL framework is preserved across evolutionary time. However, the magnitude of this regulatory content differs systematically by gene age: genes of intermediate evolutionary age consistently exhibit the highest cCRE counts across tMDL bins. Together with the tau-stratified analysis in Fig. 6B, this mid-age peak highlights an enrichment of regulatory elements in genes of intermediate evolutionary age. This pattern is consistent with the notion that recently evolved genes have not yet accumulated extensive regulatory architectures, intermediate-age genes have acquired larger cCRE repertoires, and very ancient genes achieve comparable expression-pattern complexity using fewer regulatory elements, consistent with more efficient regulatory architectures over long evolutionary timescales.

While these findings establish a clear relationship between gene age, tissue specificity, and regulatory information content, some genes deviate from this pattern. Specifically, we observed ancient genes with tissue-specific expression and recently evolved genes that are already broadly expressed. To provide biological context for these exceptions, we performed pathway enrichment analysis using Reactome annotations^74^.

Ancient tissue-specific genes were enriched for pathways in xenobiotic metabolism, small molecule transport, and amino acid metabolism (Fig. 6D), suggesting roles in cell-autonomous detoxification and metabolic processing. Such functions have been proposed to reflect cell-autonomous adaptations^75^, processes that were essential in unicellular organisms, have been conserved but are now localized to specialized tissues in multicellular species. Consistent with this interpretation, tissue enrichment analysis revealed overrepresentation in liver, kidney, and testis (Supplementary Fig. 11).

Conversely, recently evolved yet broadly expressed genes were enriched for transcriptional and innate immune pathways, including RNA Polymerase II Transcription, and interferon signaling (Fig. 6E). Many of these genes belong to the rapidly evolving zinc finger family^76,77^. Although classified as young based on sequence divergence, their broad expression and core functions suggest deeper evolutionary origins. Rapid sequence evolution may mask these deeper roots, while ancestral regulatory architecture enables their integration into essential gene networks.

Collectively, these results highlight that the relationship between gene expression complexity and regulatory architecture is modulated across evolutionary history, with genes of intermediate evolutionary age harboring the largest regulatory element repertoires for a given level of expression-pattern complexity.

## Discussion

Our study presents a conceptual framework for understanding regulatory information content in the human genome, grounded in the principle of MDL and maximum parsimony. By treating gene expression as an information compression problem in which regulatory architecture encodes the rules governing tissue-specific expression, we show that regulatory architecture scales predictably with expression-pattern complexity and evolutionary history. We propose that regulatory architecture acts as a decompression program: broadly expressed and strictly tissue-specific genes are encoded through relatively concise regulatory programs, whereas genes with complex spatial patterns require expanded regulatory architectures to resolve their higher informational demand.

A key advance of this work is our maximum parsimony-based measure quantifying the minimal number of regulatory transitions needed for a gene’s expression profile across tissues or cell types. Unlike conventional tissue specificity measures such as tau, tMDL captures the hierarchical relationships between cell types and accounts for expression variation across both closely related and distantly related cell types. One important consideration is that tMDL depends on the choice of cell-type hierarchy. We used an expression-based tree because it captures similarities in transcriptional programs between cell types, and thus their underlying regulatory environments, making it well-suited for addressing questions of regulatory similarity. We further confirmed that our results are robust to this choice by reproducing all key findings using an independently derived cell-type hierarchy based on chromatin accessibility^45^ (Supplementary Fig. 3). While cells from the same lineage are often transcriptionally similar^78^, such trees may not fully reflect developmental or differentiation relationships. Efforts to reconstruct complete developmental lineage trees of cells across the human body are advancing^79–81^ and once available, integrating them with expression-based hierarchies could further strengthen our framework.

While nearly all regulatory features scaled with our predicted regulatory demand (tMDL), only some features showed MDL-consistent trends when analyzed against tissue specificity (tau). *Cis*-regulatory features like cCRE number, intron length, and 3′ UTR length peaked among intermediate tau values, consistent with MDL and with prior reports^82–84^. In contrast, *trans*-acting features such as TFs and miRNAs increased monotonically with decreasing tau, plateauing among broadly expressed genes. This contrast reflects fundamental differences between *cis*- and *trans*-regulation: while *cis*-elements support modular, tissue-specific, and context-specific control, *trans*-regulators often act globally and are reused across many genes and tissues. The divergent patterns seen between tau and tMDL in *trans*-regulators highlight the limitations of using tissue specificity alone to infer regulatory information content. While tau effectively captures expression breadth and variability, it does not account for the hierarchical relationships between tissues or cell types, a key feature incorporated into the tMDL. This alignment with tMDL, even in the absence of a consistent trend with tau, highlights how tMDL more effectively captures regulatory demand encoded in gene expression patterns. Notably, pairwise correlations between the regulatory features investigated in this work were generally moderate to low (Supplementary Fig. 7), indicating that the observed trends are not simply due to dependencies among features.

Further, our analyses provide a quantitative framework for distinguishing “switch-like” versus “knob-like” regulatory strategies. Using our tMDL-based approach, regulatory features can be evaluated across expression regimes to determine their predominant mode. This framework could be extended to other regulatory layers or organisms for similar classification.

The relationship between expression patterns and regulatory information content is also shaped by evolutionary history. Genes of intermediate evolutionary age showed the highest regulatory information content, as reflected in their elevated cCRE content and alignment with MDL predictions, indicating that regulatory architecture follows a dynamic rather than linear trajectory. One possible explanation is that newly emerged genes, often tissue-specific, lack extensive *cis*-regulatory infrastructure and gradually acquire CREs as they integrate into regulatory networks. Over longer timescales, selective pressures on very ancient, often housekeeping genes may streamline regulation and reduce CRE abundance. In this view, intermediate-age genes may occupy a transitional phase: sufficiently evolved to have acquired diverse regulatory mechanisms but not yet constrained by the economizing pressures seen in the oldest genes.

Consistent with this view, we find that the scaling between expression-pattern complexity and regulatory architecture is not unique to humans. The same qualitative relationships between tMDL, tau, and regulatory features are preserved in mouse and extend to the evolutionarily distant *Drosophila*, despite major differences in organismal complexity, tissue composition, and regulatory annotation depth. This cross-species conservation supports the interpretation that MDL-consistent organization reflects a general principle of gene regulation rather than a human- or dataset-specific phenomenon.

In conclusion, our results show that regulatory information content scales with the informational demands of a gene’s expression pattern, consistent with MDL predictions, and is further shaped by evolutionary history. While other factors such as environmental cues, developmental programs, and network context also contribute, expression complexity emerges as a major driver of regulatory burden. By integrating expression patterns, regulatory features, and evolutionary dynamics within an information-theoretic framework, we identify a fundamental organizing principle of gene regulation.

Future work extending these analyses across a broader range of species and developmental time courses could further refine this model and clarify how regulatory complexity evolves. While our results focus on patterns within a species, the regulatory landscape itself evolves as organisms acquire more cell types, and examining how tissue specificity and regulatory information content shift across species with increasing cellular diversity could test whether MDL principles also govern macroevolutionary scaling. Equally compelling is the exploration of temporal dynamics, testing whether MDL principles apply to changes in regulatory information content during development within a tissue or organism, alongside the spatial patterns observed across tissues.

In addition, the framework introduced here motivates functional studies that examine how genes with distinct regulatory architectures respond to perturbation, stress, or regulatory rewiring. Such studies could, for example, test whether regulatory architecture influences expression stability, inducibility, or dynamic responses to environmental or genetic perturbations. More broadly, our approach illustrates how information theory can provide a quantitative framework for understanding the forces shaping genome regulation over evolutionary timescales.

## Methods

All analyses were performed using previously published datasets (see Table 1). No new human or animal subjects were involved, and therefore no additional ethical approval was required.

**Table 1:** External data sources and their usage in this study

Data type	Source	Usage in study
HPA bulk RNA-seq	HPA v24^33^	Used to calculate tissue specificity (tau)
HPA single-cell RNA-seq	HPA v24^33^	Used to calculate cell-type specificity (tau) and tMDL
cCRE-gene links	GeneHancer v5^10^	Annotated number of cCREs per gene
Alternative cCRE-gene links in mouse study	Cusanovich et al. (2018)^46^	Mouse annotated number of cCREs per gene
TF-gene interactions	hTFtarget^47^	Counting TFs per gene
miRNA-gene interactions	TarBase v9^52^	Counting miRNAs per gene
Gene structure annotations	Ensembl BioMart (Release 113)	Extracted CDS, intron, 5′UTR, and 3′UTR lengths
Mouse single-cell RNA-seq	Tabula Muris^85^	Used to calculate mouse cell-type specificity (tau) and tMDL
Fly bulk RNA-seq	Fly Atlas2^86^	Used to calculate fly tissue specificity (tau) and tMDL
Mouse and fly TF-gene interactions	TFlink^62^	Counting TFs per gene for mouse and fly
Gene evolutionary age	Litman & Stein (2019)^73^	Stratified genes into 19 evolutionary age bins

### Expression datasets

Transcript-level RNA-seq data was downloaded from the HPA website (version 24.0) on December 25, 2024^33^. Bulk tissue data includes normalized transcripts per million (nTPM) values for 40 normal human tissues based on Ensembl v109 annotation. Single-cell derived data includes nTPM values across 81 annotated cell types from 31 tissues.

Mouse single-cell RNA-seq data were obtained from Tabula Muris database^85^. Expression values were normalized per cell to total read depth and log-transformed using Seurat’s *LogNormalize* procedure. The cell identities were set to the annotated cell type, and the average expression per cell type was calculated using the *AverageExpression* function in Seurat.

*Drosophila melanogaster* expression data were obtained from FlyAtlas2 bulk RNA-seq measurements^86^. Adult tissues were selected, and where both male and female samples were available, expression values were averaged across sexes.

### Calculation of tissue specificity (tau) indices

We calculated the tau index separately for bulk and single-cell datasets to quantify the tissue specificity of each gene. The tau index ranges from 0 (ubiquitous expression) to 1 (highly specific expression) and was computed following the formula by Yanai et al.^4^.:

$$\tau=\frac{\mathop{\sum }\limits_{i=1}^{n}(1-{\hat{x}}_{i})}{n-1}$$

In this formula, *n* is the number of tissues or cell types, and *xᵢ* is the expression value in tissue or cell type *i*, normalized by the gene’s maximum expression across all *n* samples.

Expression values were set to zero if < 1 and subsequently log-transformed (log(x + 1)). After excluding genes that were not detected across any tissue or cell type, the tau index was computed for 18,234 protein-coding genes.

### Gene enrichment analyses

The GO term enrichment was performed with the GOrilla web tool^35^. Separate ranked-list analyses were run for each tissue specificity class. For the high tau set, genes were ranked in descending order of tau; for the low tau set, in ascending order; for the intermediate tau set, by absolute deviation from tau = 0.5 ( | tau–0.5 | , smallest to largest). Default parameters were used in single-ranked-list mode, and enrichment *p*-values were adjusted by the tool’s built-in Benjamini-Hochberg procedure.

The Reactome pathway enrichment was performed with the Enrichr web interface (Reactome 2024 library)^87^. Ancient tissue-specific gene sets were defined as those with tau ≥ 0.8 and assigned to the Holozoa or earlier evolutionary age groups (666 genes total). New ubiquitous genes were defined as those with tau ≤ 0.4 and assigned to the Euteleostomi or more recent age groups (1,316 genes total). *P*-values were corrected using the Benjamini-Hochberg method. Tissue-level enrichment for these same gene sets was assessed using the TissueEnrich web tool (https://tissueenrich.gdcb.iastate.edu/) with default settings^88^.

### cCRE annotations and gene-cCRE links

cCRE annotations and gene-cCRE links were obtained from GeneHancer^10^, which integrates data from multiple sources and assigns confidence scores to predicted gene targets. We performed all analyses using both the full dataset (419,020 cCREs) and the high-confidence “Double Elite” subset (122,815 cCREs), in which both the enhancer itself and its link to the target gene are supported by at least two independent sources, providing stronger evidence for the regulatory association. In the full dataset, each gene was linked to an average of ~55 enhancers, and each enhancer to ~7 genes; in the Double Elite subset, each gene was linked to ~7 enhancers, and each enhancer to ~6 genes. Results were consistent across both sets; primary analyses used the full set, with Double Elite results shown in Supplementary Fig. 2.

The orthogonal single-study cCRE analysis (supplementary Fig. 6) was derived from a mouse single-cell ATAC-seq atlas^46^. Enhancer-gene associations were defined using the authors’ Cicero co-accessibility links. Distal accessible regions were linked to genes if co-accessibility with a proximal (TSS-associated) site was ≥ 0.2. For distal regions lacking such links, the strongest remaining association with co-accessibility ≥ 0.1 was retained. cCRE counts were computed as the number of unique distal regions linked to each gene.

### Construction of cell-type similarity tree and calculation of tMDL scores

Gene expression profiles (values < 1 set to zero and log-transformed) across 81 cell types were used to construct a cell-type similarity dendrogram. Pairwise correlations between cell types were computed using Spearman’s correlation coefficient, and a hierarchical clustering (Ward’s method) was performed on the correlation-based distance matrix (distance = 1−Spearman correlation). The resulting linkage matrix was converted into a rooted dendrogram.

To quantify the tMDL for each gene, expression levels across cell types were discretized into six bins based on the global distribution of expression values, with zero-expressed values assigned to the lowest bin. Using this discretization, Fitch’s parsimony algorithm^44^ was applied to the dendrogram, computing the minimal number of state transitions required to explain each gene’s binned expression pattern across cell types. The algorithm assigns possible expression states to internal nodes by working recursively from the leaves toward the root. At each internal node, the intersection of expression states from its descendant nodes is taken; if this intersection contains at least one common state, it is assigned to the node without incrementing the count. However, if there is no common state (i.e., the intersection is empty), the node is assigned the union of descendant states, and the count of required state transitions is incremented by one. The final sum of these increments across the dendrogram was reported as the tMDL score for each gene.

The implementation of the tMDL framework is provided in Supplementary Code 1.

tMDL was additionally computed using an externally defined cell-type hierarchy derived from a published chromatin accessibility-based tree^45^, constructed from genome-wide single-cell ATAC-seq profiles across human tissues. Cell types overlapping between this hierarchy and the HPA single-cell dataset were identified based on matching or near-matching annotation names (34 cell types), and tMDL was recomputed.

Unless otherwise specified, tau values were derived from bulk RNA-seq tissue samples from the HPA, to maintain consistency with prior large-scale studies and enable comparisons. In contrast, tMDL requires a hierarchy based on expression similarity, which at the tissue level can be confounded by shared or contaminating cell populations. For example, tissues with high blood or immune cell infiltration may appear transcriptionally similar due to expression from circulating cells rather than true similarity between the tissues. We therefore calculated tMDL using single-cell RNA-seq data aggregated by cell type, which mitigates these issues and produces a more biologically meaningful hierarchy. Gene expression patterns were highly concordant between bulk and single-cell datasets (Supplementary Fig. 1A), and complementary analyses using tau from cell-type data and tMDL from tissue data produced overall similar trends (Supplementary Fig. 12), supporting the robustness of our conclusions.

### Trend analyses across ranked tau and tMDL values

For analyses examining trends across ranked tau or tMDL values, genes were ordered by the metric of interest and grouped into non-overlapping bins defined by fixed intervals of the metric. Summary statistics (mean or median, as indicated) were calculated within each bin.

### Stratified analysis of intermediate-tau genes by tMDL

Genes were ranked by tau, and an intermediate-tau subset was defined using a rank-based approach. Unless otherwise specified, intermediate tau was defined as the middle 30% of genes by tau rank; an additional, more stringent analysis was performed using the middle 10% of tau-ranked genes (Supplementary Fig. 5). Within each intermediate-tau subset, genes were ranked by tMDL, and split into low- and high-tMDL groups as the bottom and top 20% of tMDL values, respectively. Distributions of regulatory features between low- and high-tMDL groups were compared using two-sided Mann-Whitney U tests. Effect sizes (r) for Mann-Whitney U tests were calculated as Z / √N.

All genomic analyses were conducted using the GRCh38 (hg38) human genome assembly.

### Reporting summary

Further information on research design is available in the Nature Portfolio Reporting Summary linked to this article.

## Supplementary information

Supplementary Information

Description of Additional Supplementary Files

Supplementary Data 1

Supplementary Data 2

Supplementary Code 1

Reporting Summary

Transparent Peer Review file

## Data Availability

All data used in this study are publicly available from previously published sources. The datasets and their usage are summarized in Table 1. Complete gene-level annotations and computed features underlying the analyses presented in this study, including tau, tMDL, and regulatory feature annotations, are provided in Supplementary Data 1 (human) and Supplementary Data 2 (mouse).

## Code availability

The code used to compute the tMDL scores using Fitch’s parsimony algorithm is provided as Supplementary Code 1.
