# Supplementary material for: An information content principle explains regulatory patterns of gene expression across human tissues
Source: Nat Commun. 2026 Apr 11;17:5064. doi: 10.1038/s41467-026-71279-1 (PMC13243617; doi:10.1038/s41467-026-71279-1)
Supplement: Supplementary file 1 — Supplementary Information [file 41467_2026_71279_MOESM1_ESM.pdf]

Supplementary information

An information content principle explains regulatory patterns of gene expression across human tissues

Ruthie Golomb[1], Maayan Yoles[1^], Simon Fishilevich[1^], Bar Cohen[1], Sapir Savariego Peled[2], Dvir Dahary[1], David Gokhman[1], Yitzhak Pilpel[1*]

Author Affiliations

[1] Department of Molecular Genetics, Weizmann Institute of Science, Rehovot 7600001, Israel

[2] Department of Molecular Cell Biology, Weizmann Institute of Science, Rehovot 76100001, Israel

*corresponding author: pilpel@weizmann.ac.il

Supplementary items:

Supplementary Figures 1-12

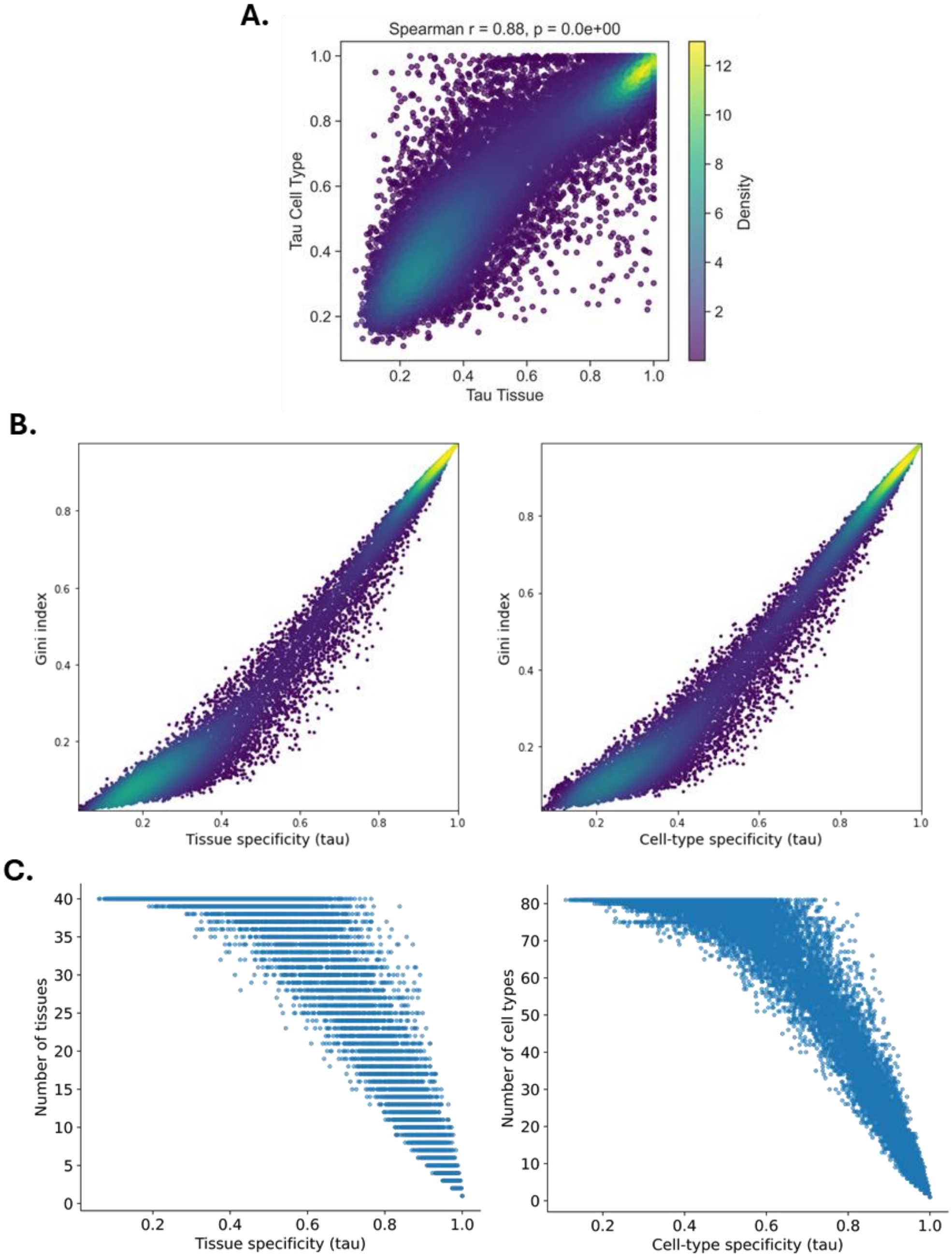

Supplementary Figure 1. Comparison of tissue specificity metrics and expression breadth across bulk and single-cell datasets. (A) Scatterplot showing the correlation between tissue-level tau (derived from bulk RNA-seq) and cell type-level tau (calculated from single-cell RNA-seq aggregated by cell type) for 18,324 human protein-coding genes. Points are colored by local density. **(B)** Scatterplots showing the relationship between tau and the Gini index, a complementary measure of expression inequality. Left: tissue-level; Spearman $\rho = 0.98$, $p < 1 \times 10^{-300}$, $n = 18,324$ genes. Right: cell type-level; Spearman $\rho = 0.98$, $p < 1 \times 10^{-300}$, $n = 18,324$ genes. **(C)** Scatterplots comparing tau values to expression breadth, measured as the number of tissues or cell types in which each gene is expressed. Left: tissue-level tau vs. number of tissues. Right: cell type-level tau vs. number of cell types.

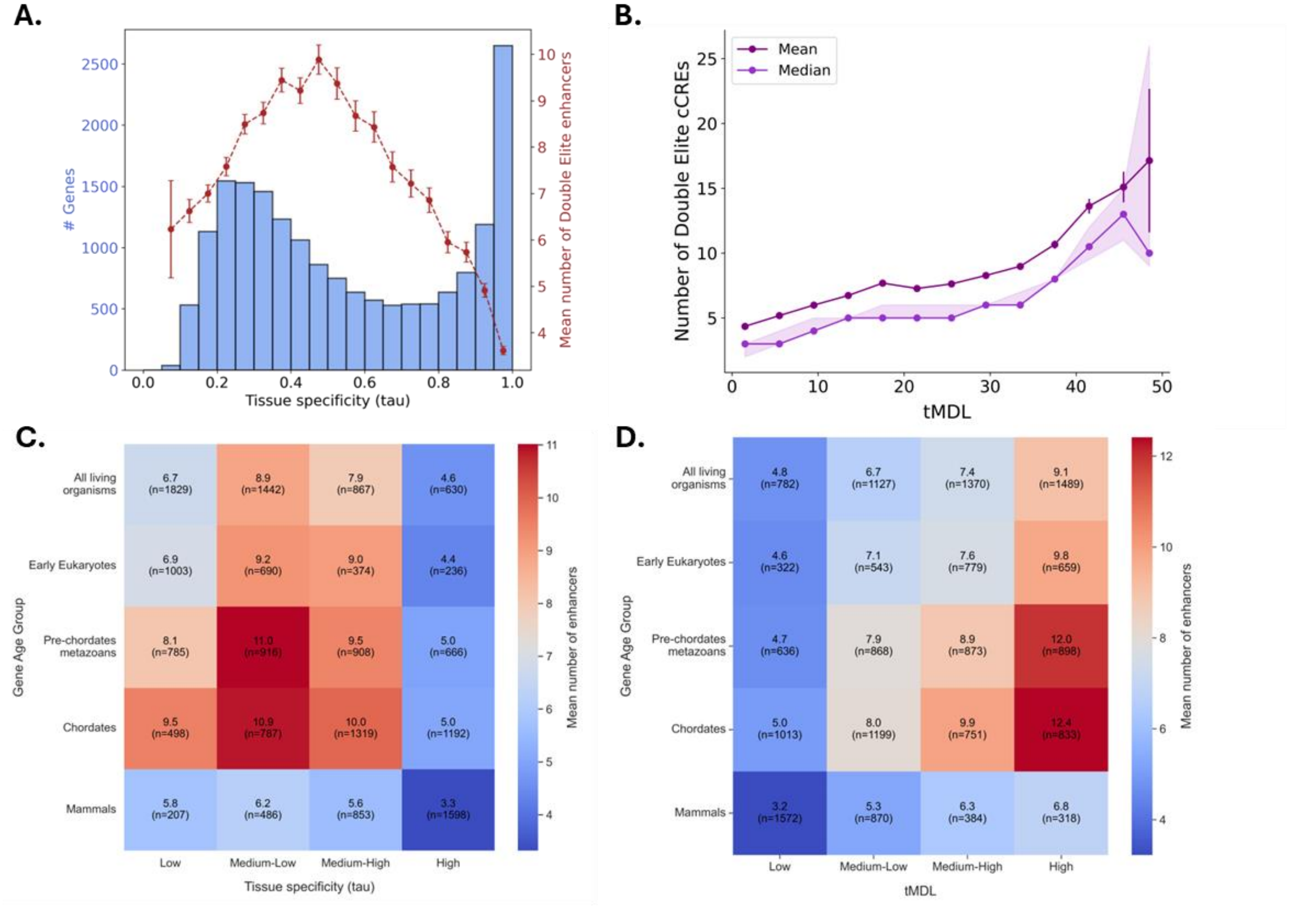

Supplementary Figure 2. cCRE-based analyses performed on the more stringent "Double Elite" subset. All panels replicate the analyses shown in the main text using only Double Elite candidate cis-regulatory elements (cCREs), as defined by GeneHancer (see Methods), revealing patterns consistent with those observed using the full cCRE set. **(A)** Histogram of tau scores calculated from bulk RNA-seq data (gray bars, left y-axis), with overlaid line plot (red dashed line, right y-axis) indicating the mean number of linked Double Elite cCREs per gene within each tau bin. Error bars represent the 90% confidence interval (CI) of the mean. **(B)** Line plot showing the mean (purple) and median (violet) number of Double Elite cCREs per gene across non-overlapping fixed-interval bins of ranked tree-aware minimum description length (tMDL). Error bars and shaded areas represent the 90% confidence interval (CI) of the mean. **(C)** Heatmaps displaying the mean number of Double Elite CREs per gene, stratified by evolutionary age (y-axis) and tissue specificity (x-axis, binned by tau values). **(D)** Heatmaps displaying the mean number of Double Elite CREs per gene, stratified by evolutionary age (y-axis) and tMDL (x-axis). All analyses were performed on n = 18,234 human protein-coding genes.

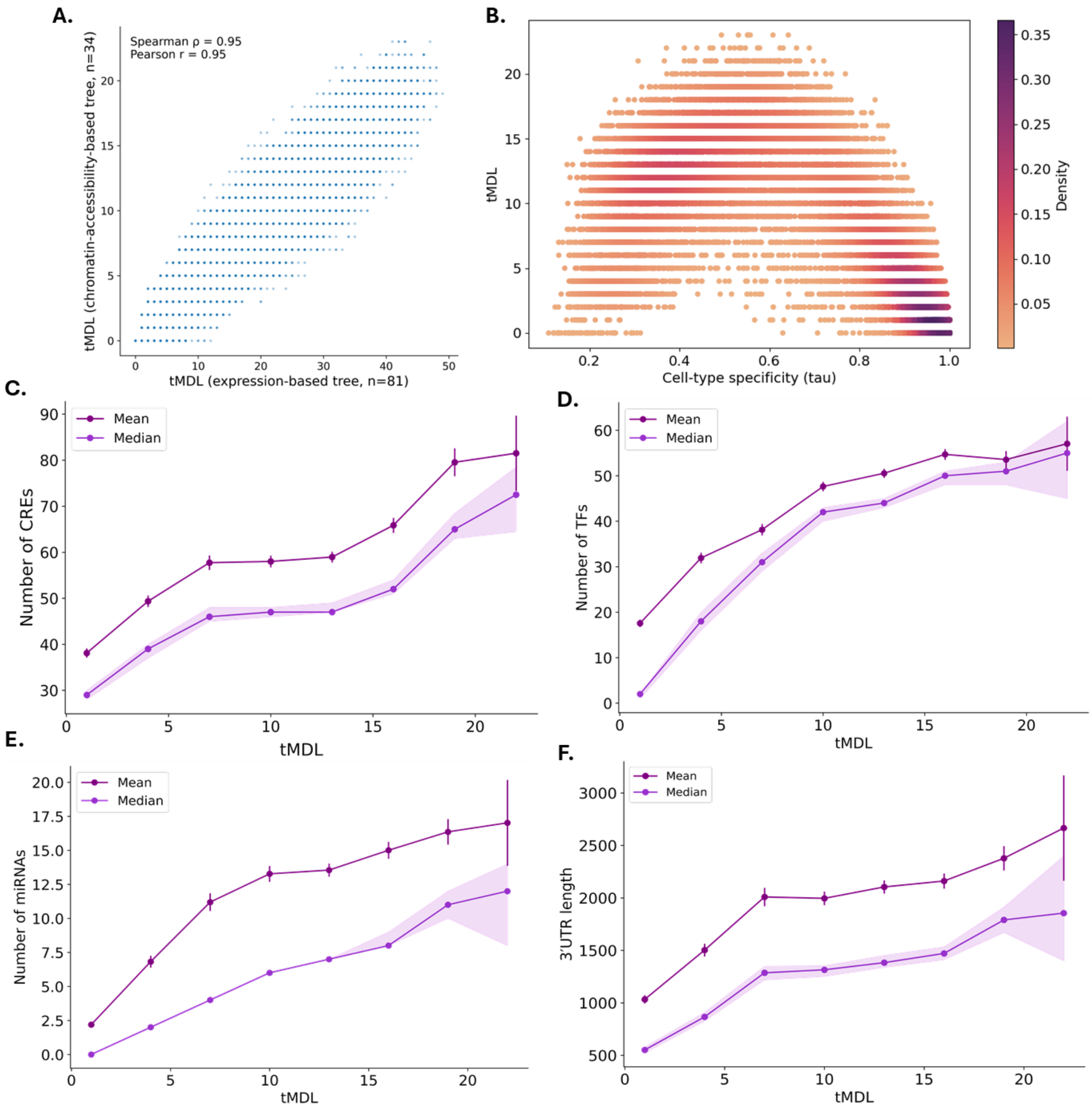

Supplementary Figure 3. Robustness of tMDL to cell-type hierarchy construction. The computation of tree-aware minimum description length (tMDL) depends on the structure of the underlying cell-type hierarchy, which can be defined using different biological data modalities. To assess the robustness of tMDL to this choice, we recomputed tMDL using an independent, chromatin accessibility-based cell-type hierarchy (Zhang et al., 2021) and repeated all downstream analyses using this alternative topology. **(A)** Scatterplot comparing tMDL values computed using the expression-based and chromatin-based hierarchies. **(B)** Scatterplot showing the relationship between cell-type specificity (tau) and tMDL using the chromatin-based hierarchy. Each dot represents a gene, with color indicating local density. **(C–F)** Line plots showing the mean (purple) and median (violet) values of regulatory features per gene, computed in non-overlapping fixed-interval bins along ranked tMDL values. Error bars and shaded areas represent 90% confidence intervals for the mean and median, respectively. **(C)** Number of *cis*-regulatory elements (CREs) per gene. **(D)** Number of transcription factors (TFs) per gene. **(E)** Number of miRNAs per gene. **(F)** 3′UTR length. All analyses were performed on n = 18,234 human protein-coding genes.

A. Number of bins = 3

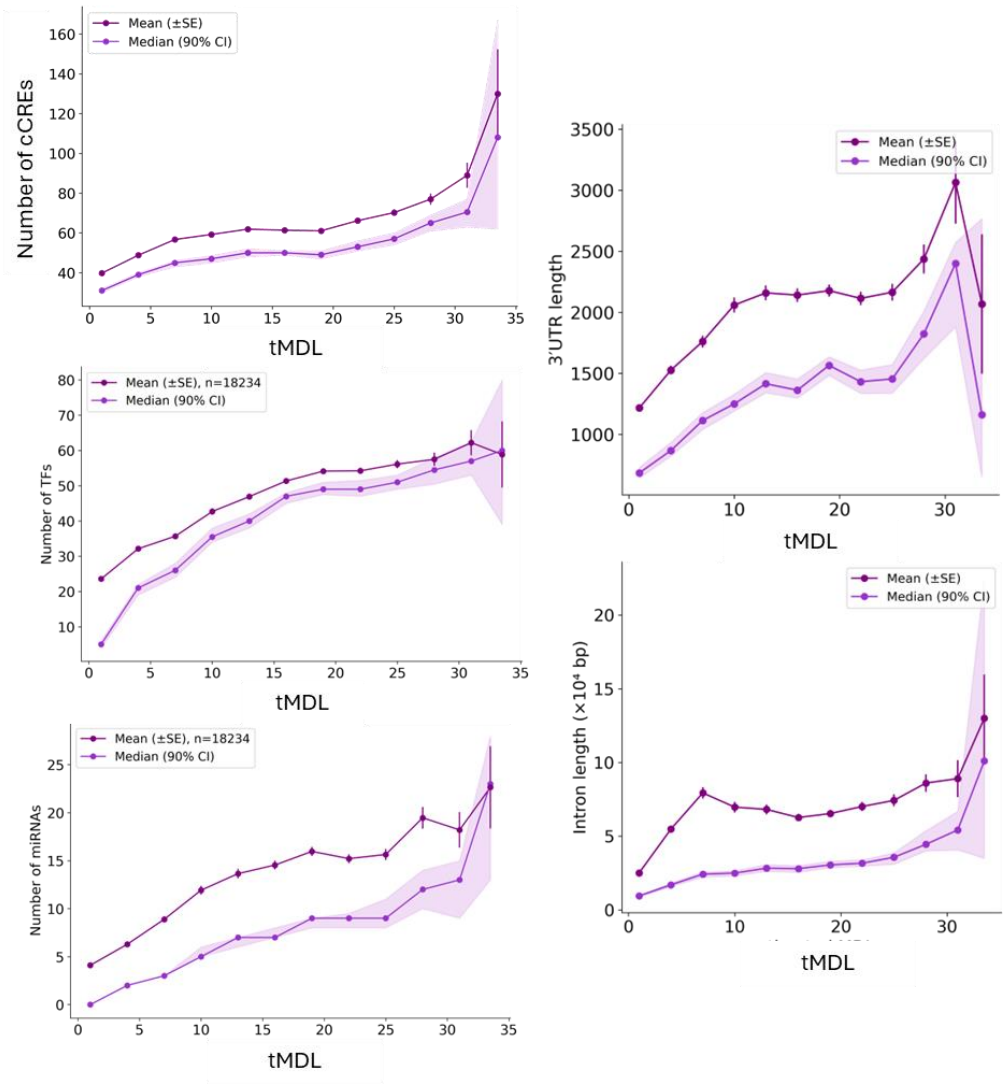

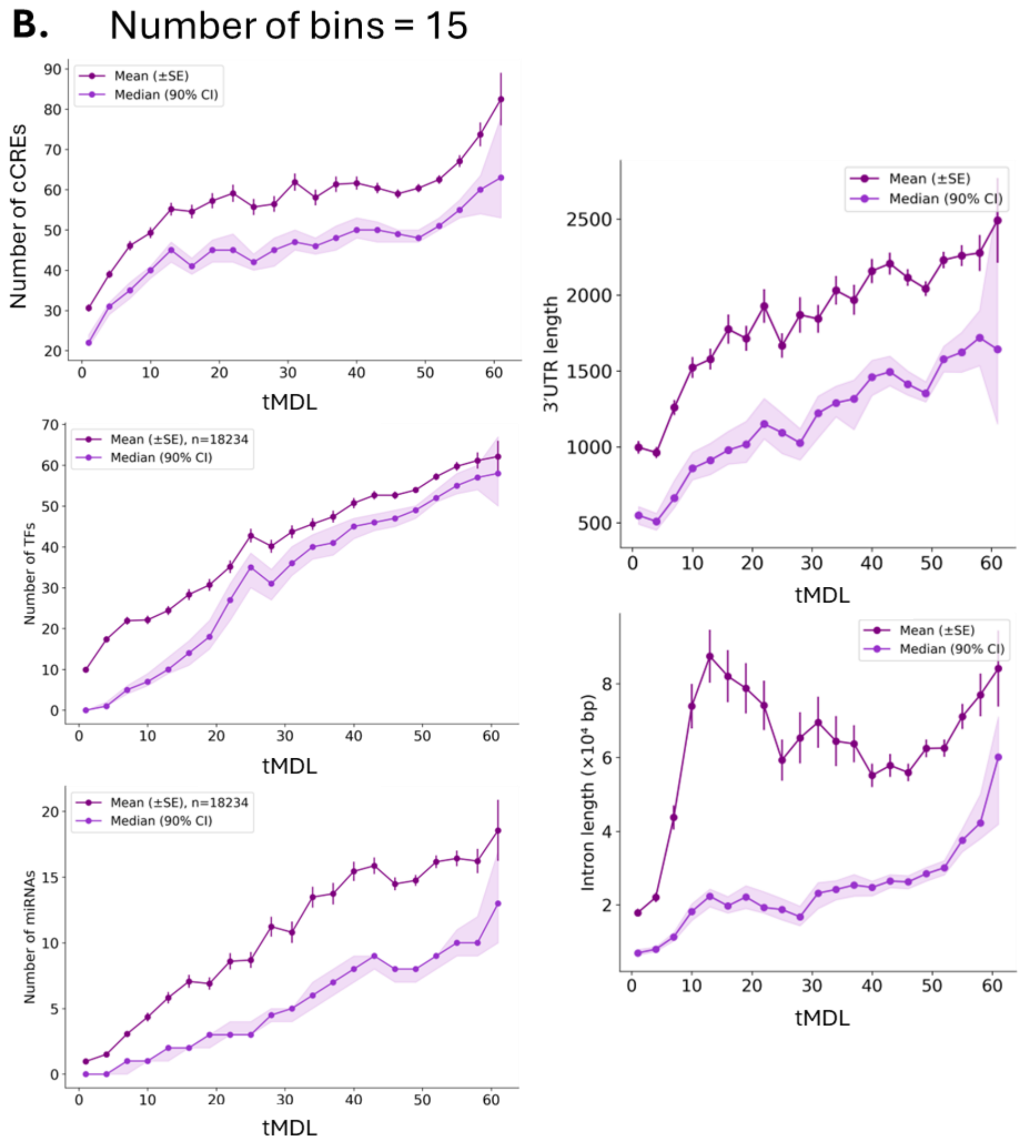

Supplementary Figure 4. Robustness of tMDL trends to expression binning parameters. The tMDL calculation requires discretizing continuous gene expression levels into bins, which can influence the number of inferred regulatory transitions. To test the robustness of downstream results to this choice, we repeated the tMDL-based analysis using alternative expression binning strategies. Line plots show the mean (purple) and median (violet) values of key regulatory features across non-overlapping fixed-interval bins of ranked tMDL. **(A)** Results using 3 expression bins. **(B)** Results using 15 expression bins. Features shown (left to right): number of linked candidate *cis*-regulatory elements (cCREs), number of transcription factors (TFs), number of miRNAs, 3′ UTR length, and intron length. In all cases, trends were overall consistent with the main analysis (six bins; see Figure 4), supporting the robustness of the tMDL framework to binning choice. Error bars and shaded areas represent 90% confidence intervals (CI). All analyses were performed on n = 18,234 human protein-coding genes.

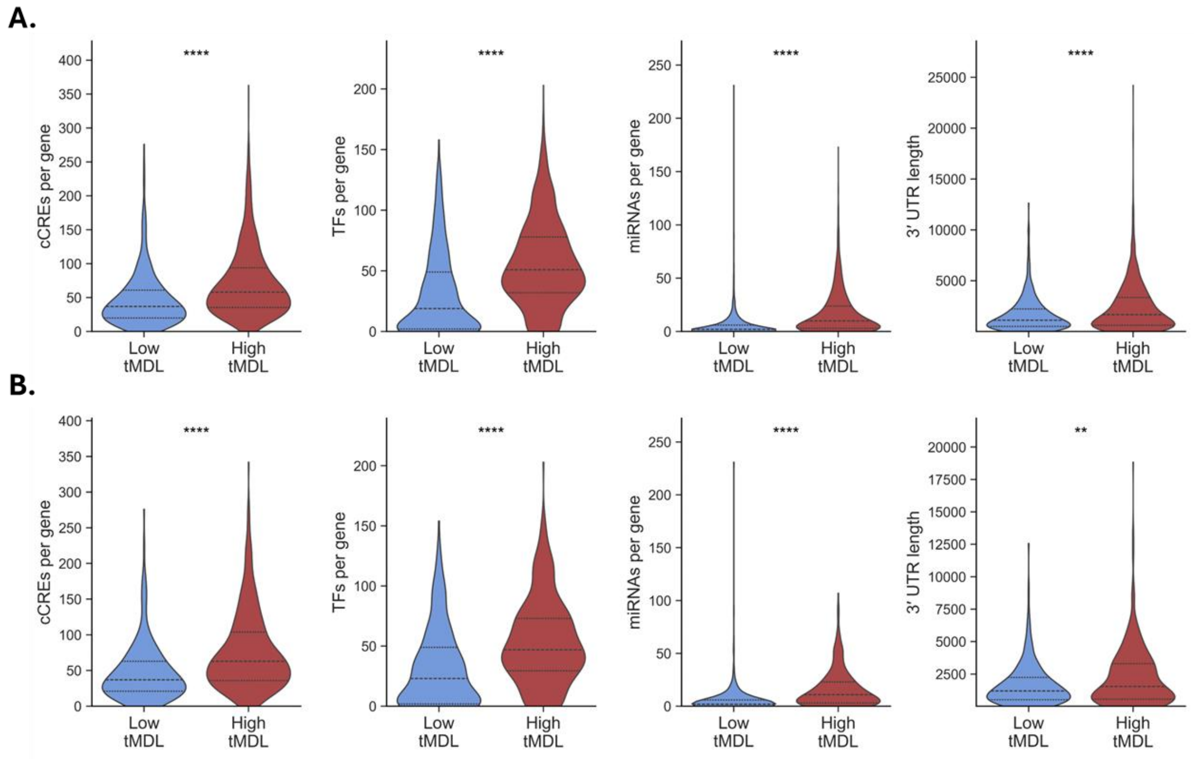

Supplementary Figure 5. Comparison of regulatory features between low- and high-tMDL genes within intermediate tau. Genes with intermediate tau values were divided into low- and high-tMDL groups (bottom and top 20%), and regulatory features were compared between these groups. Violin plots show the distributions of regulatory features. Significance was assessed using two-sided Mann–Whitney U tests. Asterisks denote $p < 0.01$ (**), < 0.001 (***), and < 0.0001 (****) **(A) I**ntermediate tau defined as the middle 30% of genes by tau (n = 5,470 genes; low tMDL n = 1,185; high tMDL n = 1,215). **(B) I**ntermediate tau defined as the middle 10% of genes by tau (n = 1,824 genes; low tMDL n = 367; high tMDL n = 387). Features shown (left to right): number of candidate *cis*-regulatory elements (cCREs), number of transcription factors (TFs), number of miRNAs, and 3′ UTR length.

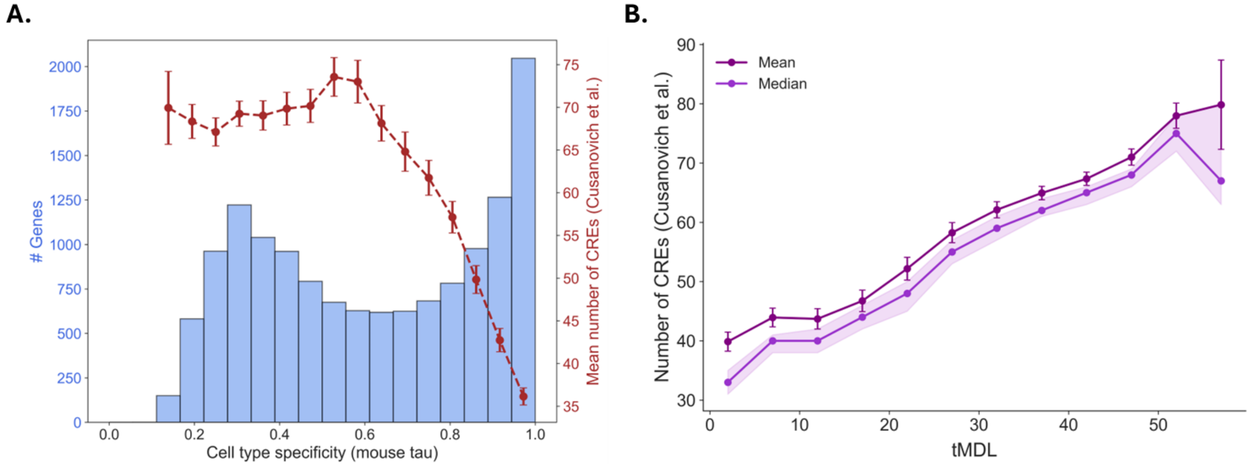

Supplementary Figure 6. cCRE-based analyses performed using a single-study regulatory map. To assess the robustness of candidate cis-regulatory elements (cCREs)-based results to the source of the regulatory map, cCRE-gene associations were derived from a single-study mouse scATAC-seq atlas (Cusanovich et al., 2018) using Cicero co-accessibility links, and downstream analyses were repeated using this alternative regulatory baseline. **(A)** Histogram of tau scores (blue bars, left y-axis), with an overlaid line plot (red dashed line, right y-axis) indicating the mean number of cCREs linked to genes within each tau bin; error bars represent the 90% confidence interval (CI) of the mean. **(B)** Line plot showing the mean (purple) and median (violet) number of cCREs per gene, computed in fixed-interval non-overlapping bins along ranked tMDL values. Error bars and shaded areas represent 90% CIs for the mean and median, respectively. Both analyses were performed on n = 14,008 mouse genes

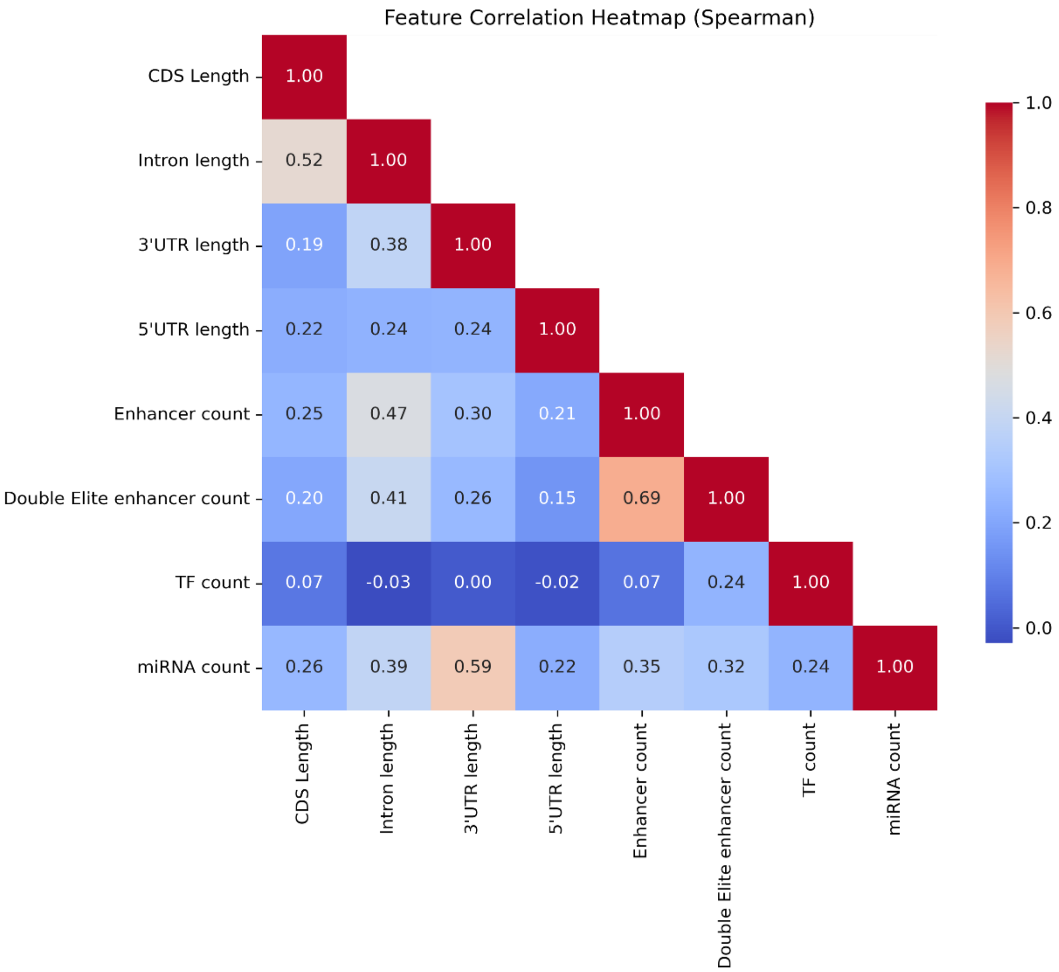

Supplementary Figure 7. Correlation structure among gene regulatory and structural features. Heatmap showing pairwise Spearman correlations (n = 18,234 genes) between key gene features, including coding sequence (CDS) length, intron length, 3′ UTR length, 5′ UTR length, number of linked candidate cis-regulatory elements (cCREs) (full and Double Elite subsets), and the number of TFs and miRNAs per gene. The color scale reflects the correlation coefficient (ρ).

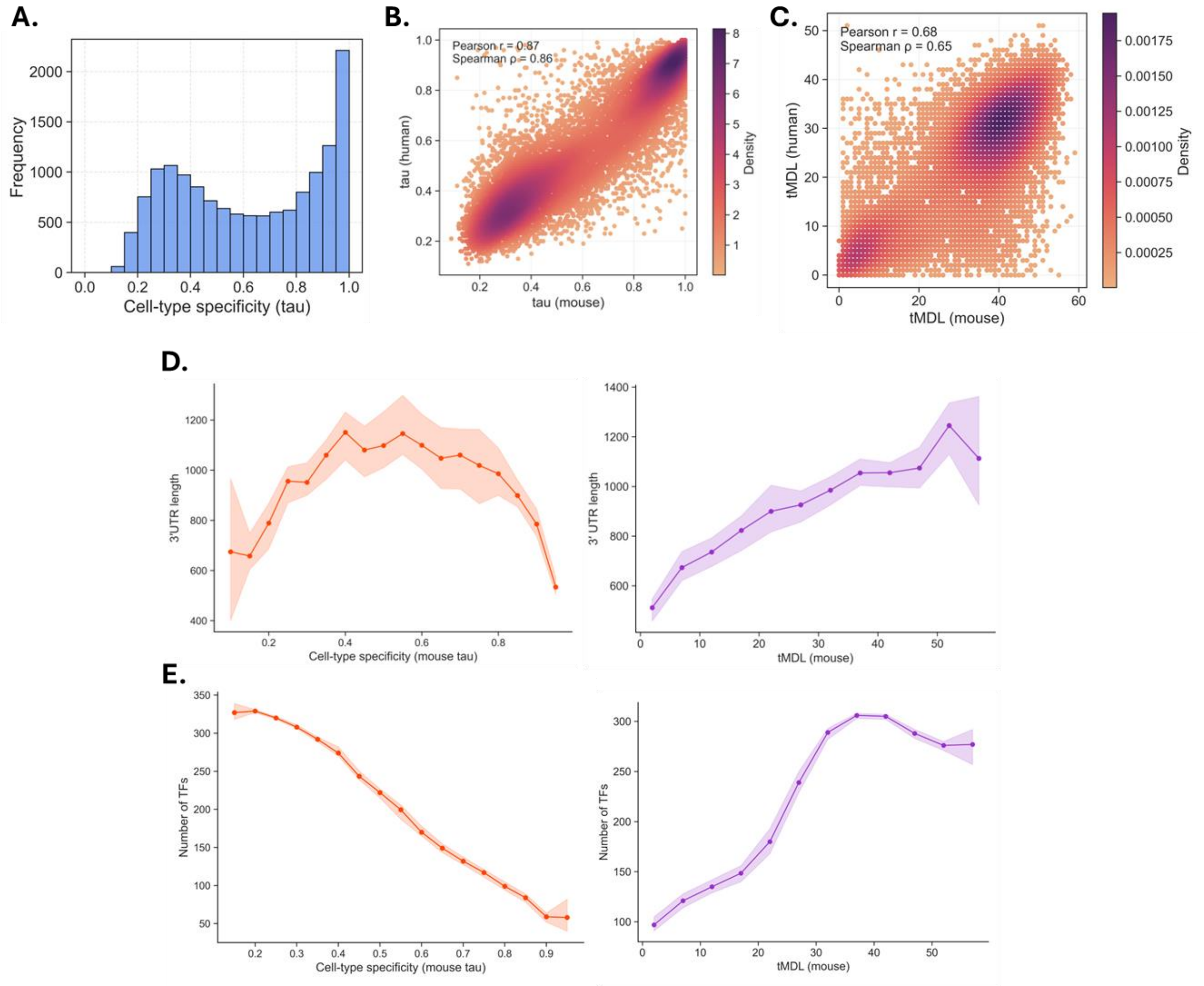

A.
Frequency
Cell-type specificity (tau)
B.
Pearson r = 0.87
Spearman ρ = 0.86
tau (human)
tau (mouse)
Density
C.
Pearson r = 0.68
Spearman ρ = 0.65
tMDL (human)
tMDL (mouse)
Density
D.
3'UTR length
Cell-type specificity (mouse tau)
3' UTR length
tMDL (mouse)
E.
Number of TFs
Cell-type specificity (mouse tau)
Number of TFs
tMDL (mouse)

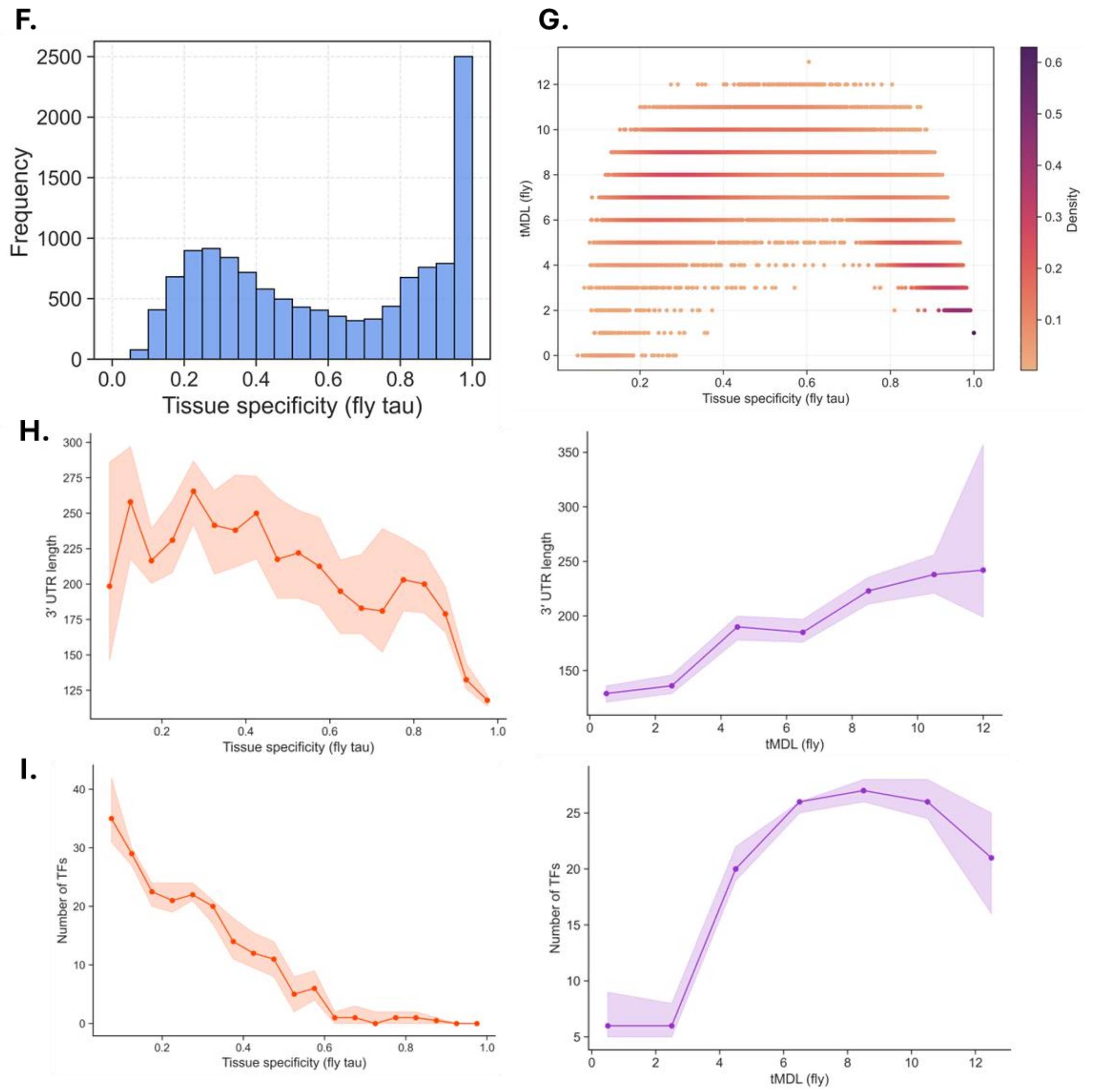

Supplementary Figure 8. Cross-species robustness of the relationships between tau, tMDL, and regulatory features. To assess whether the coupling between tree-aware minimum description length (tMDL), expression specificity (tau), and regulatory features extends beyond humans, analogous analyses were performed in mouse and *Drosophila* using available transcriptomic and regulatory annotations. **(A)** Distribution of cell-type specificity (tau) scores for mouse protein-coding genes, calculated from single-cell RNA-seq data aggregated by cell type (Tabula Muris; n = 14,696 genes). **(B)** Cross-species comparison of cell-type specificity. Scatterplot showing mouse tau versus human tau for one-to-one orthologous genes (n= 10,713 genes); points are colored by local density (Pearson r = 0.87, $p < 1 \times 10^{-300}$; Spearman ρ = 0.88, $p < 1 \times 10^{-300}$). **(C)** Cross-species comparison of tMDL. Scatterplot showing mouse tMDL versus human tMDL for the same set of orthologous genes, colored by local density (Pearson r = 0.68, $p < 1 \times 10^{-300}$; Spearman ρ = 0.65, $p < 1 \times 10^{-300}$). **(D–E)** In mouse, median 3′ UTR length (D) and transcription factor (TF) count (E) per gene across non-overlapping fixed-interval bins of ranked tau (left) or tMDL (right); shaded regions indicate 90% confidence intervals (CI). **(F)** Distribution of tissue specificity (tau) scores for *Drosophila melanogaster* protein-coding genes, calculated from bulk RNA-seq across 15 tissues (n =

12,631 genes). **(G)** Scatterplot showing the relationship between tau and tMDL across all fly genes; each point represents a gene and is colored by local density, revealing the characteristic horseshoe-shaped pattern (n = 12,631 genes). **(H–I)** In fly, median 3′ UTR length (H) and TF count (I) across non-overlapping fixed-interval bins of ranked tau (left) or tMDL (right); shaded regions indicate 90% CI.

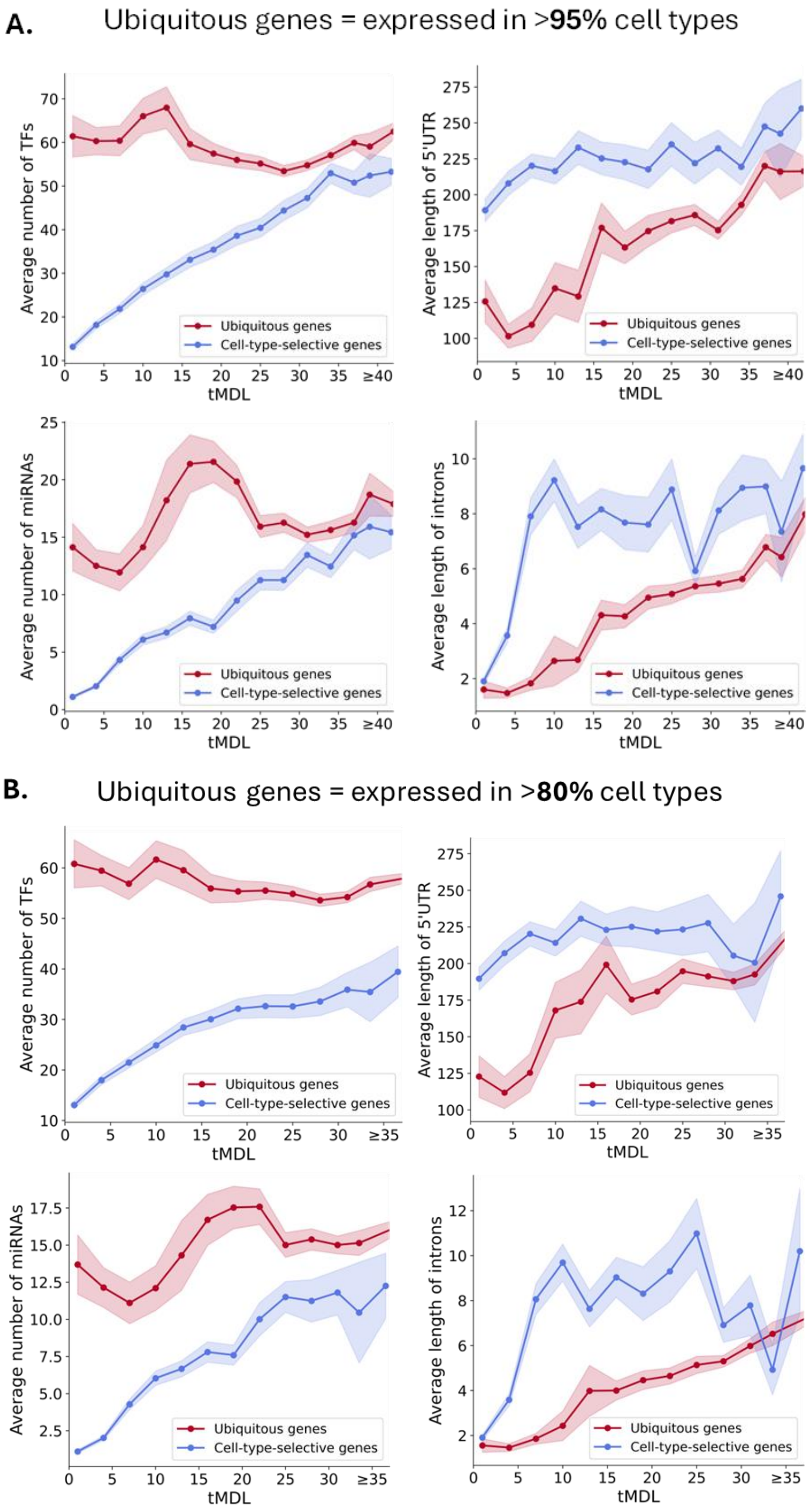

Supplementary Figure 9. Robustness of knob- and switch-like regulatory regimes to the definition of ubiquitous expression. To assess whether the results in Figure 4 depend on the cutoff used to define ubiquitous versus cell-type-selective genes, the analysis was repeated using alternative thresholds for this division. Line plots show mean feature values calculated across non-overlapping fixed-interval bins of

ranked tMDL, separately for ubiquitous (red) and cell-type-selective (blue) genes. Shaded areas represent 90% confidence intervals. To avoid very sparse windows, all genes with tMDL ≥ 35 or ≥ 40 (depending on the cutoff) were grouped into a final bin. Analyses were performed on n = 18,234 genes total. **(A)** Ubiquitous genes defined as those expressed in ≥ 95% of tissues (n=7,808 genes). **(B)** Ubiquitous genes defined as those expressed in ≥ 80% of tissues (n=9,961 genes). Regulatory features are shown in the following order: number of transcription factors (TFs) (top left), number of miRNAs (bottom left), 5′ UTR length (top right), and intron length (bottom right).

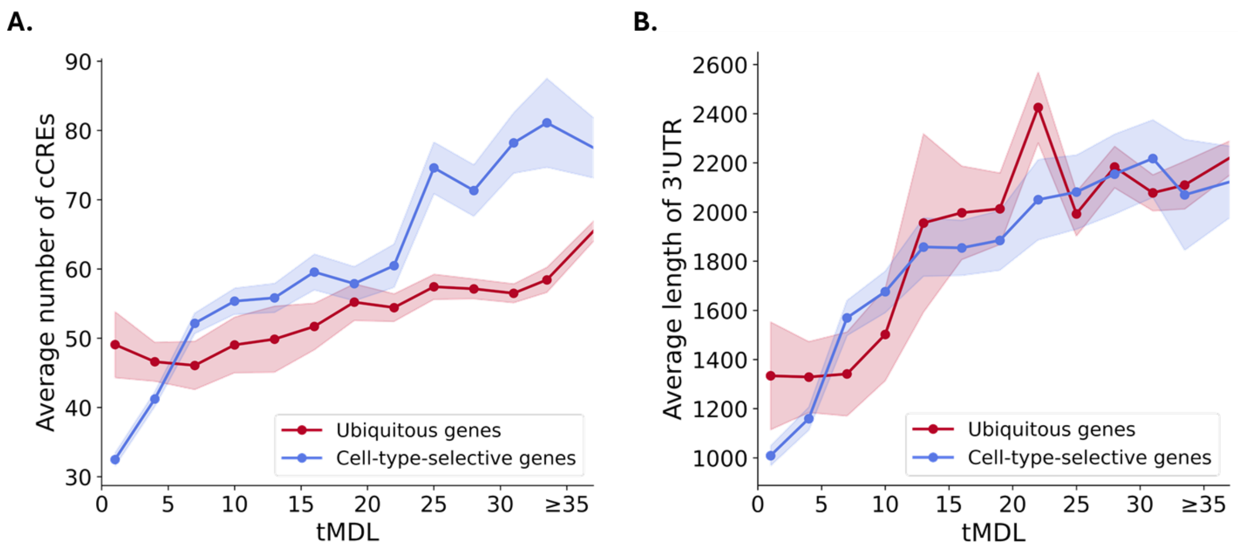

Supplementary Figure 10. Regulatory features that scale with tMDL independently of expression breadth. Line plots showing the relationship between tree-aware minimum description length (tMDL) and two key regulatory features, candidate *cis*-regulatory elements (cCREs) abundance and 3′ UTR length, stratified by expression breadth. Mean feature values were calculated across non-overlapping fixed-interval bins of ranked tMDL, separately for ubiquitous (red) and cell-type selective (blue) genes. Shaded areas represent 90% confidence intervals (CI). To avoid sparse windows, all genes with tMDL ≥ 40 were grouped into a final bin. Both cCRE count and 3′ UTR length increased with tMDL in each regime, highlighting their universal contribution to regulatory complexity across diverse expression strategies.

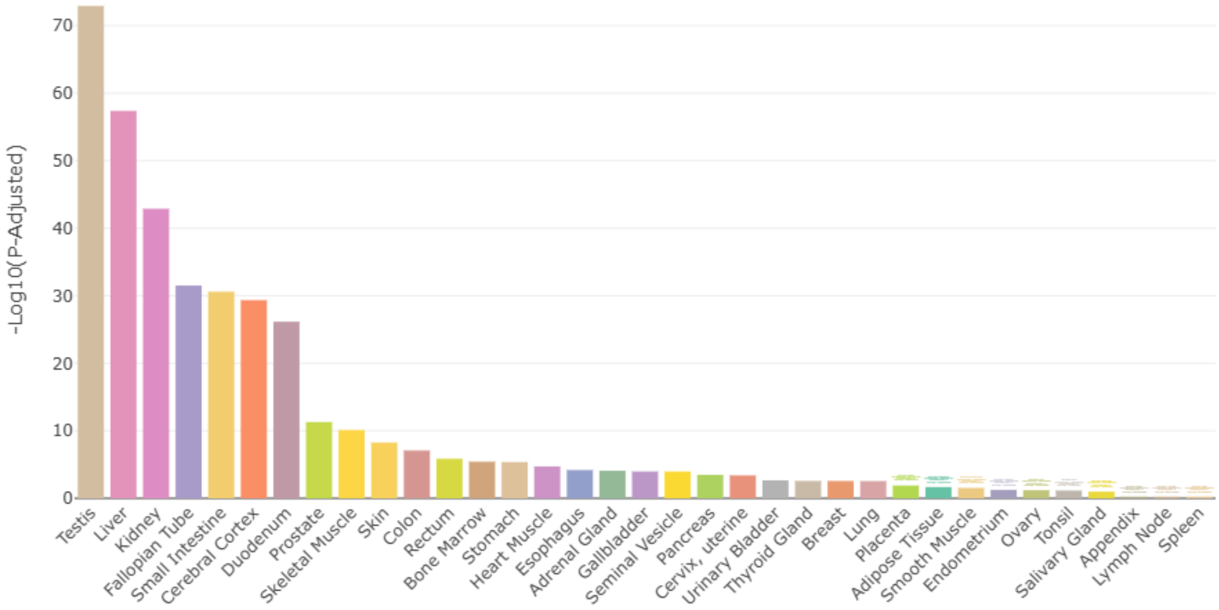

Supplementary Figure 11. Tissue enrichment of ancient tissue-specific genes. Bar plot showing tissue enrichment analysis results for ancient genes with tissue-specific expression patterns (n=1,145 genes). Enrichment was assessed using TissueEnrich, which tests for overrepresentation of input genes among tissue-enriched gene sets defined in the Human Protein Atlas. The three top enriched tissues are testis, liver, and kidney.

A.

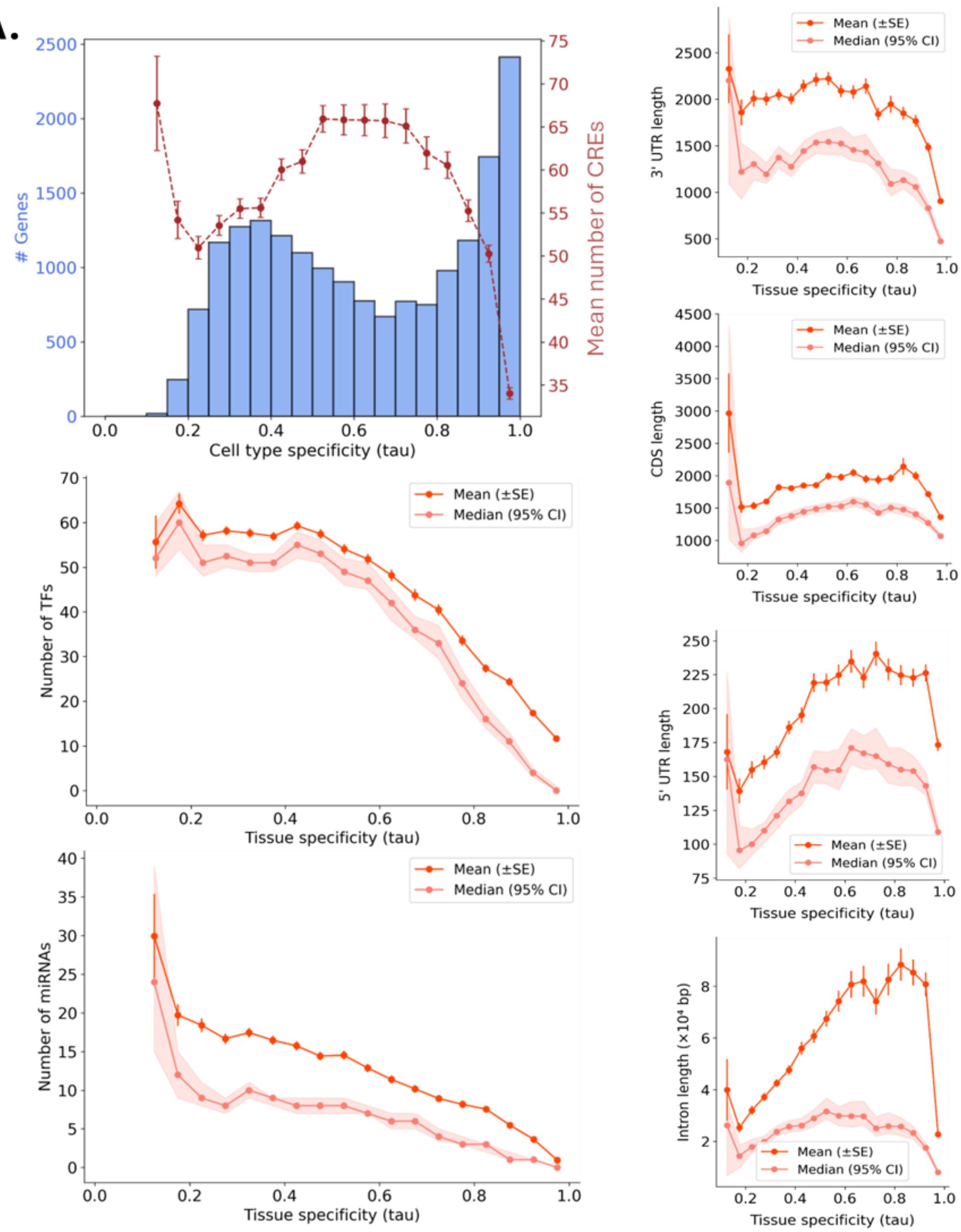

Genes
Mean number of CREs
Cell type specificity (tau)
Number of TFs
Mean (±SE)
Median (95% CI)
Tissue specificity (tau)
Number of miRNAs
3' UTR length
CDS length
5' UTR length
Intron length (×10⁴ bp)

B.

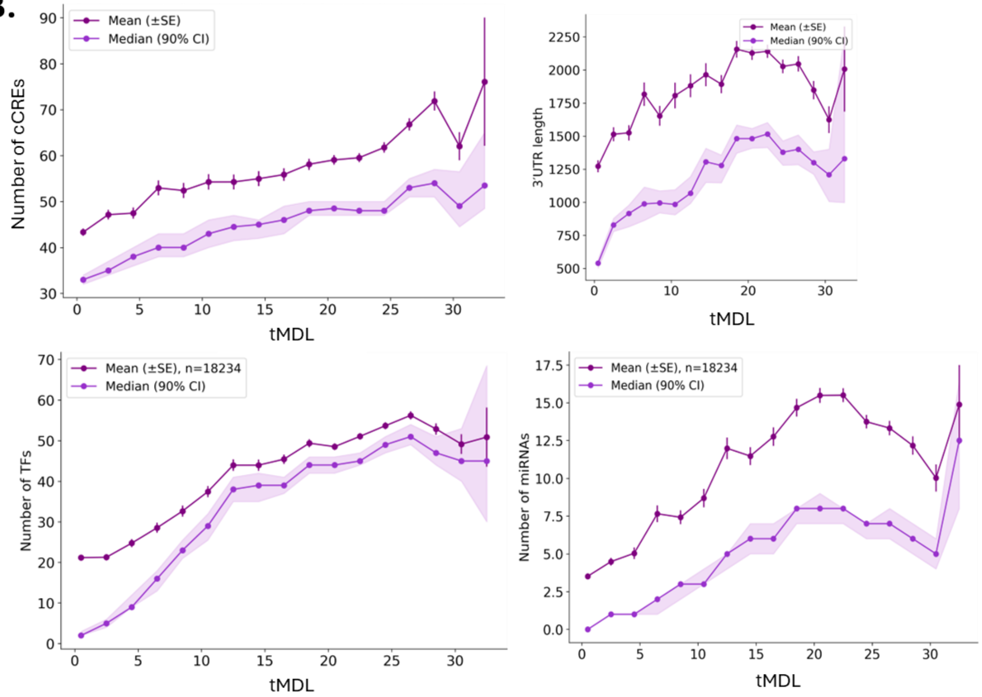

Supplementary Figure 12. Robustness of regulatory and structural feature trends across tissue-level and cell type-level definitions of tau and tMDL. (A) Regulatory and structural feature trends across tau calculated from single-cell RNA-seq data aggregated by cell type, showing overall agreement with results using tissue-level tau. The first panel shows a histogram of tau values (blue bars, left y-axis) with an overlaid line plot (red dashed line, right y-axis) indicating the mean number of linked candidate cis-regulatory elements (cCREs) per gene within each tau bin. Subsequent panels show the mean (purple) and median (violet) values of transcription factor (TF) count, miRNA count, 3′ UTR length, CDS length, 5′ UTR length, and intron length across fixed-interval bins of tau. **(B)** Regulatory feature trends across tMDL calculated from bulk RNA-seq tissue data, showing overall agreement with the main analyses based on cell-type-derived tMDL. Line plots display the mean and median number of linked cCREs, TFs, miRNAs, and 3′ UTR length across fixed-interval bins of tMDL. No consistent signal was observed for intron length in this tissue-level analysis. Error bars and shaded areas represent 90% confidence intervals.
