# Supplementary material for: An information content principle explains regulatory patterns of gene expression across human tissues
Source: Nat Commun. 2026 Apr 11;17:5064. doi: 10.1038/s41467-026-71279-1 (PMC13243617; doi:10.1038/s41467-026-71279-1)
Supplement: Supplementary file 2 — Description of Additional Supplementary Files [file 41467_2026_71279_MOESM2_ESM.pdf]

Description of Additional Supplementary Files

Supplementary Data 1: Human gene-level annotations and derived features used in this study.

Supplementary Data 2: Mouse gene-level annotations and derived features used in this study.

Supplementary Code 1: Custom python code to calculate the tMDL score
