# Supplementary material for: An information content principle explains regulatory patterns of gene expression across human tissues
Source: Nat Commun. 2026 Apr 11;17:5064. doi: 10.1038/s41467-026-71279-1 (PMC13243617; doi:10.1038/s41467-026-71279-1)
Supplement: Supplementary file 6 — Reporting Summary [file 41467_2026_71279_MOESM6_ESM.pdf]

nature portfolio

Corresponding author(s): Yitzhak Pilpel

Last updated by author(s): March 08, 2026

Reporting Summary

Nature Portfolio wishes to improve the reproducibility of the work that we publish. This form provides structure for consistency and transparency in reporting. For further information on Nature Portfolio policies, see our Editorial Policies and the Editorial Policy Checklist.

Statistics

For all statistical analyses, confirm that the following items are present in the figure legend, table legend, main text, or Methods section.

n/a	Confirmed	
☐	☒	The exact sample size (*n*) for each experimental group/condition, given as a discrete number and unit of measurement
☐	☒	A statement on whether measurements were taken from distinct samples or whether the same sample was measured repeatedly
☐	☒	The statistical test(s) used AND whether they are one- or two-sided<br>*Only common tests should be described solely by name; describe more complex techniques in the Methods section.*
☒	☐	A description of all covariates tested
☐	☒	A description of any assumptions or corrections, such as tests of normality and adjustment for multiple comparisons
☐	☒	A full description of the statistical parameters including central tendency (e.g. means) or other basic estimates (e.g. regression coefficient) AND variation (e.g. standard deviation) or associated estimates of uncertainty (e.g. confidence intervals)
☐	☒	For null hypothesis testing, the test statistic (e.g. *F*, *t*, *r*) with confidence intervals, effect sizes, degrees of freedom and *P* value noted<br>*Give P values as exact values whenever suitable.*
☒	☐	For Bayesian analysis, information on the choice of priors and Markov chain Monte Carlo settings
☒	☐	For hierarchical and complex designs, identification of the appropriate level for tests and full reporting of outcomes
☐	☒	Estimates of effect sizes (e.g. Cohen's *d*, Pearson's *r*), indicating how they were calculated

Our web collection on statistics for biologists contains articles on many of the points above.

Software and code

Policy information about availability of computer code

Data collection	No software was used for data collection.
Data analysis	Analyses were performed using in-house Python (version 3.12.8) and R (version 4.3.1) scripts. Core Python packages included numpy, pandas, scipy, matplotlib, seaborn, and Biopython. For the enrichment analyses, publicly available GOrilla and Enrichr software were used. The tMDL framework is implemented in a custom Python script provided as Supplementary Code 1.

For manuscripts utilizing custom algorithms or software that are central to the research but not yet described in published literature, software must be made available to editors and reviewers. We strongly encourage code deposition in a community repository (e.g. GitHub). See the Nature Portfolio guidelines for submitting code & software for further information.

Data

Policy information about availability of data

All manuscripts must include a data availability statement. This statement should provide the following information, where applicable:

- Accession codes, unique identifiers, or web links for publicly available datasets
- A description of any restrictions on data availability
- For clinical datasets or third party data, please ensure that the statement adheres to our policy

This study analyzed publicly available datasets from the following sources: Human Protein Atlas v24 (https://www.proteinatlas.org/about/download), hTFtarget (https://guolab.wchscu.cn/hTFtarget#!/), TarBase v9 (https://dianalab.e-ce.uth.gr/tarbasev9), Ensembl Biomart (Release 113), Tabula Muris (https://biohub.org/sf/tabula-muris/), Fly Atlas2 (http://flyatlas.org/atlas.cgi), and TFlink (https://tflink.net/). GeneHancer v24 data was provided by the GeneCards team upon request.

Additional datasets were sourced from published literature, including evolutionary gene age assignments from Litman & Stein (2019) and mouse cCRE-gene links from Cusanovich et al. (2018).

Research involving human participants, their data, or biological material

Policy information about studies with human participants or human data. See also policy information about sex, gender (identity/presentation), and sexual orientation and race, ethnicity and racism.

Reporting on sex and gender	N/A
Reporting on race, ethnicity, or other socially relevant groupings	N/A
Population characteristics	N/A
Recruitment	N/A
Ethics oversight	N/A

Note that full information on the approval of the study protocol must also be provided in the manuscript.

Field-specific reporting

Please select the one below that is the best fit for your research. If you are not sure, read the appropriate sections before making your selection.

☒ Life sciences ☐ Behavioural & social sciences ☐ Ecological, evolutionary & environmental sciences

For a reference copy of the document with all sections, see nature.com/documents/nr-reporting-summary-flat.pdf

Life sciences study design

All studies must disclose on these points even when the disclosure is negative.

Sample size	Analyses were performed on publicly available datasets. The sample sizes reflect the number of genes, gene features, and regulatory elements present in these resources and were determined by the availability of data in these public repositories.
Data exclusions	Genes that had no expression detected in any of the tissues or cell types in the Human Protein Atlas data were not included in the analysis.
Replication	Patterns described in the manuscript (e.g., scaling of regulatory features with tissue specificity and tMDL) were consistently observed across independent datasets, alternative regulatory maps, and different cell-type hierarchies, supporting reproducibility. No experimental replication was applicable to this computational study.
Randomization	No experimental samples were allocated in this study. Analyses were performed on publicly available datasets, and gene groupings (e.g., tissue specificity bins, evolutionary age categories) were defined by analytical or external criteria rather than random assignment. Randomization was therefore not applicable.
Blinding	Blinding is not relevant to this study.

Reporting for specific materials, systems and methods

We require information from authors about some types of materials, experimental systems and methods used in many studies. Here, indicate whether each material, system or method listed is relevant to your study. If you are not sure if a list item applies to your research, read the appropriate section before selecting a response.

Materials & experimental systems

n/a	Involved in the study	
☒	☐	Antibodies
☒	☐	Eukaryotic cell lines
☒	☐	Palaeontology and archaeology
☒	☐	Animals and other organisms
☒	☐	Clinical data
☒	☐	Dual use research of concern
☒	☐	Plants

Methods

n/a	Involved in the study	
☒	☐	ChIP-seq
☒	☐	Flow cytometry
☒	☐	MRI-based neuroimaging

Plants

Seed stocks	Not relevant.
Novel plant genotypes	Not relevant.
Authentication	Not relevant.
