# Supplementary material for: An information content principle explains regulatory patterns of gene expression across human tissues
Source: Nat Commun. 2026 Apr 11;17:5064. doi: 10.1038/s41467-026-71279-1 (PMC13243617; doi:10.1038/s41467-026-71279-1)
Supplement: Supplementary file 7 — Transparent Peer Review file [file 41467_2026_71279_MOESM7_ESM.pdf]

nature portfolio

Peer Review File

An information content principle explains regulatory patterns of gene expression across human tissues

Corresponding Author: Professor Yitzhak Pilpel

This file contains all reviewer reports in order by version, followed by all author rebuttals in order by version.

Version 0:

Reviewer comments:

Reviewer #1

(Remarks to the Author)
The Golomb et al. manuscript presents a novel computational framework that combines the Minimum Description Length principle from information theory with phylogenetic parsimony to characterize how CREs, transcription factors, microRNAs, scale with tissue specificity. They calculate tMDL scores, which quantifies the minimal transitions required for each gene's expression pattern. They identify distinct patterns in tissue-specific versus housekeeping genes. Their analysis reveals that these patterns correlate to gene age, with alignment between tMDL and CRE counts peaking in intermediate-aged genes.

Expertise:
I cannot assess the information theory aspects.
Comments:

The MDL framework addresses a fundamental question of why gene regulatory strategies vary across genes. Their finding of switch-like and knob-like regulation is interesting and can guide design of regulatory systems and lends more questions for future studies. Overall, the manuscript is well-written, and the methodologies presented are sound and clearly described. The major concern however is that the study lacks functional validation. Their study is observational, finding that tMDL scores are correlated to the number of regulatory elements. This study would greatly benefit from a screen for CREs of genes with different tMDL scores.

Their use of expression-based cell-type tree is novel. How would other methods of creating a hierarchy of cell types affect the measurement of tMDL?

Does their suggested principle hold across species? Testing this could establish whether MDL is universal in gene regulation.

Minor comments:
Would the authors publish code or a tool to calculate tMDL?

Reviewer #2

(Remarks to the Author)
I co-reviewed this manuscript with one of the reviewers who provided the listed reports. This is part of the Nature Communications initiative to facilitate training in peer review and to provide appropriate recognition for Early Career Researchers who co-review manuscripts.

Reviewer #3

(Remarks to the Author)
This is an important manuscript about fundamental aspects of gene regulation. I believe it is a very important work that will be foundational to many future studies. Golomb, Pilpel and colleagues decipher the breadth of gene expression according to cis-regulatory elements using principles from information theory. This is a power lens and so the manuscript will represent an important contribution to the literature.

I just have a few critics that may help the authors in improving the clarity of the work. A main claim of the paper is that (from the abstract) “Two patterns emerged: features scaling with MDL in selectively expressed genes tend to act as on/off switches, whereas those in ubiquitous genes serve as fine-tuning knobs” However it’s not clear if the “knob” versus “switch” dichotomy is seen as a result or an assumption by the authors. In line 409, for example it is stated as an assumption: “These genes are expected to require fine-tuned, quantitative control, hence refer to here as “knob-like” regulation.” I suggest that the authors better define this dichotomy and what evidence tests would be able to falsify the assertion.

Moreover, in this analysis the grouping in the Figure 4 analysis is based on expression in <90% of tissues as being selective. But a gene expressed in say 80% of the tissues still sounds as pretty ubiquitous. To make a stronger analysis the authors may choose to examine how their results depend on this cutoff.

In Line 330 the authors write: “Interestingly, this increase in TF count plateaued around tau ≈ 0.4, suggesting that once a gene is widely expressed across all tissues, additional TFs provide diminishing returns.” It’s not clear how the graph in Figure 3A supports this assertion since there are not significantly more TFs in genes with tau<0.4.

Also the authors should explain what would explain why the more broadly expressed genes would be targeted by the most TFs.

Figure 4 is a bit difficult to understand and I’m wondering if the authors can show the analysis in a better way. For example, does it really make sense to talk about slope in this analysis?

One minor point; Figure 1B could also indicate the tMDL score.

Reviewer #4

(Remarks to the Author)
Golomb et al. analyze tissue specificity (T) and a tree-aware MDL metric (tMDL), relating both to regulatory features (CREs, TFs, miRNAs, gene age). The core message, that regulatory demand peaks for genes with intermediate specificity, is interesting and worth publishing. But key analyses lean heavily on aggregate resources and author-defined hierarchies. Several conclusions could be artifacts of dataset choice or construction of the cell-type tree. Below are concrete ways to make this paper truly robust.

Major concerns

1. Dependence on aggregate databases → request: validate with single-cell atlases (mouse + human).
The tissue-specificity results hinge on HPA (bulk and aggregated scRNA-seq) and compiled cCRE sets. Please replicate the T and tMDL findings using one or more internally consistent single-study atlases with many cell types, ideally both species. For example, you could use the Qiu et al. mouse time series data, and pseudobulk RNA-seq profiles (or other equivalently diverse datasets that are based on a single single cell study w/ very diverse cell types rather than aggregate). Is T similarly distributed? Are gene ranks stable by HPA vs. other datasets? Are specificity classifications of individual genes stable? Are these values and classifications also preserved across mouse vs. human?

2. cCRE counts may mirror sampling/aggregation bias → request: add orthogonal CRE baselines based on single studies.
The inverted-U in CRE counts could reflect what’s been profiled, e.g. aggregation artifact. Please add either or both of two orthogonal validations: 1) Single-study regulatory maps (similar point, you don’t have to use these particular studies but they are just examples -- human: e.g., Domcke-style; mouse: Cusanovich-style or equivalent). Ideally redo CRE-per-gene vs τ and vs tMDL from scratch with the CREs/links defined within one study. 2) Conservation-only enhancer prior (no biochemical data): use noncoding conservation “pockets” matching enhancer-like sequence features and link by nearest-gene/ABC-style proximity as a sensitivity check.

3. tMDL may depend on how you built the cell-type hierarchy → request: also try to use external hierarchies.
tMDL is only as credible as the tree, which you built. Can you try using at least one external, pre-defined cell-type ontology/hierarchy (e.g., from the atlas itself, e.g. Qiu 2024 of mouse embryogenesis) to recompute tMDL and show the main results are stable?

4. I may have missed this, but a more nuanced analysis of the gene age in relation to “very high” vs. “very low’ vs. “intermediate” Tau would be helpful. I might expect ancient genes to be overwhelmingly housekeeping, while genes universal to metazoans might have high Tau/low MDL, and then mammalian specific genes (e.g. TFs with complex roles) to be more intermediate and have larger regulatory footprints. I think that this section could be presented in a much more interesting way than the current GO analyses, etc. For example, pointing out specific developmental TFs that have large (or small) footprints and complex (or simple) expression patterns, relating that to their age, etc. This is listed as a major comment but I might categorize it more as a ‘missed opportunity’...

Additional points

1. The study applies analyses to two datasets—one tissue-based and one cell-type-based—but it is not always clear which dataset is used in each analysis and figure (e.g., Fig. 2A, 2C).

2. The tau score treats each tissue independently when measuring tissue specificity. A more systematic approach would be to construct a hierarchical structure, recognizing that some tissues are more closely related to each other than to others. For example, two genes with similar tau scores may have very different implications if one is expressed in closely related cell types and the other in distantly related ones. While the author acknowledges this concern in the manuscript, they do not provide an optimized approach to address it and still rely on tau to measure tissue or cell-type specificity.

3. Although the authors state that the tau score is the most robust metric for measuring tissue specificity of gene expression, tissue specificity is a complex question—particularly since it is the central focus of this study—and may require more careful consideration. Is a single, simple metric sufficient to capture such complexity? Can genes with very low expression lead to misleading tau scores (e.g., a scatter plot of gene expression vs. tau)? How well does it perform across datasets with different numbers of cell types, varying profiling depths (i.e., data sparsity), and diverse count distributions? Could different normalization or scaling methods affect the robustness of tau? Might complementing tau with additional metrics provide more informative insights? It would also be useful to examine how tau correlates with other measures on the same datasets and whether they identify the same sets of cell-type-specific genes. All these questions require careful benchmarking.

4. Some cutoff selections appear arbitrary and require further justification. For example, the author uses 0.5 as a rough cutoff —genes with tau < 0.5 are considered broadly expressed across tissues, while genes with tau > 0.5 are thought to have progressively restricted expression. However, from Fig. 1A and S1B, there does not appear to be a clear cutoff at 0.5 that separates genes into distinct expression patterns. Genes with tau = 0.6 or even 0.7 can still be expressed across a broad range of tissues, making the choice of 0.5 questionable. Therefore, in Fig. 1C–E, dividing genes into low, tau = 0.5, and high groups is not convincing. Another unclear example is the division of genes into two groups—expressed in >90% versus <90% of cell types—as shown in Fig. 4A.

5. The relationship between tau and tMDL is not surprising given how they are calculated. As shown in Fig. 2B–C, genes with low or high tau scores have relatively uniform expression across cell types, while genes with intermediate tau scores show more heterogeneous expression, which explains their higher tMDL. A more interesting approach for subsequent analysis would be to split genes into four groups: low tau; intermediate tau with low tMDL; intermediate tau with high tMDL; and high tau.

6. The tMDL calculation seems somewhat circular, as the cell-type tree is built based on gene expression similarity, and then genes correlated with this structure are further evaluated. In addition, the author could consider using alternative methods to benchmark tMDL, e.g., https://github.com/landau-lab/PATH.

7. When calculating tMDL, the methods indicate that expression levels across cell types for each gene were discretized into six bins. I am not sure how this approach handles genes with roughly uniform expression across cell types, such as DHX15.

8. The second half of the paper conducts multiple systematic analyses to investigate regulatory features related to tissue specificity and regulatory demand, which are way more interesting. However, none of these analyses go very deep, either in terms of methodology or in providing additional validation or benchmarking, which makes the results less convincing. I would suggest that the author streamline the first half of the manuscript—quickly introducing the concepts of cell-type specificity and regulatory demand (tMDL)—and instead place greater emphasis on how regulatory features correlate with specificity.

9. Can the authors provide (sorry if we missed this) clean lists of human gene names/IDs along w/ associated T, MDL and tMDL values? Ideally, e.g. if you follow some of the suggestions above, it would also be reportable as a single clean file for mouse genes as well. The field would benefit from such a table that avoids the dichotomization of housekeeping vs. developmental genes and instead presents a more nuanced, continuous view.

Version 1:

Reviewer comments:

Reviewer #1

(Remarks to the Author)
The authors have addressed my concerns. However, code for the new analysis performed in the revisions are missing.

(Remarks on code availability)

Reviewer #2

(Remarks to the Author)
I co-reviewed this manuscript with one of the reviewers who provided the listed reports. This is part of the Nature Communications initiative to facilitate training in peer review and to provide appropriate recognition for Early Career Researchers who co-review manuscripts.

(Remarks on code availability)
The code submitted in Supplementary Code 1 is only for tMDL score calculation (with binned gene expression values).

While the Methods section explain their analysis in detail, the code for other analyses and plotting is severely lacking. In its current state, this data is not reproducible.

Reviewer #3

(Remarks to the Author)
The authors have satisfyingly addressed all my comments and suggestions.
This is an exciting piece of work.

(Remarks on code availability)

Reviewer #4

(Remarks to the Author)
The authors have done a terrific job, in our view, of addressing the comments of us and the other reviewers, and we wholeheartedly recommend publication.

(Remarks on code availability)

Open Access This Peer Review File is licensed under a Creative Commons Attribution 4.0 International License, which permits use, sharing, adaptation, distribution and reproduction in any medium or format, as long as you give appropriate credit to the original author(s) and the source, provide a link to the Creative Commons license, and indicate if changes were made.
In cases where reviewers are anonymous, credit should be given to 'Anonymous Referee' and the source.
The images or other third party material in this Peer Review File are included in the article's Creative Commons license, unless indicated otherwise in a credit line to the material. If material is not included in the article's Creative Commons license and your intended use is not permitted by statutory regulation or exceeds the permitted use, you will need to obtain permission directly from the copyright holder.
To view a copy of this license, visit https://creativecommons.org/licenses/by/4.0/

Response to Reviewers

Manuscript title: An information content principle explains regulatory patterns of gene expression across human tissues

Reviewer #1

The Golomb et al. manuscript presents a novel computational framework that combines the Minimum Description Length principle from information theory with phylogenetic parsimony to characterize how CREs, transcription factors, microRNAs, scale with tissue specificity. They calculate tMDL scores, which quantifies the minimal transitions required for each gene's expression pattern. They identify distinct patterns in tissue-specific versus housekeeping genes. Their analysis reveals that these patterns correlate to gene age, with alignment between tMDL and CRE counts peaking in intermediate-aged genes.

Expertise:
I cannot assess the information theory aspects.

Comments:
The MDL framework addresses a fundamental question of why gene regulatory strategies vary across genes. Their finding of switch-like and knob-like regulation is interesting and can guide design of regulatory systems and lends more questions for future studies. Overall, the manuscript is well-written, and the methodologies presented are sound and clearly described.

We thank the reviewer for their positive assessment of our work. We are gratified that the reviewer found our computational framework novel and our findings regarding "switch-like" and "knob-like" regulation interesting and relevant for the design of future regulatory studies.

The major concern however is that the study lacks functional validation. Their study is observational, finding that tMDL scores are correlated to the number of regulatory elements. This study would greatly benefit from a screen for CREs of genes with different tMDL scores.

We thank the reviewer for this thoughtful comment and agree that functional perturbation of regulatory elements is a natural and important next step for testing how regulatory architecture implements expression-pattern complexity. We have carefully considered what such validation would entail in the context of our framework.

Importantly, the predictions made by the tMDL framework concern aggregate regulatory architecture - that is, how regulatory information is distributed across many CREs and across a hierarchy of cell types - rather than the effect of individual CREs in a single cellular context. As a result, a direct functional test of these predictions would require perturbing multiple CREs per gene, across multiple cell types, to meaningfully alter expression-pattern complexity rather than simply modulating expression level in one condition. Because many enhancers are cell-type-specific, perturbation in a single context would be insufficient to test the core claims of the framework.

Such experiments are in principle feasible, but would constitute a substantial independent study involving large-scale, multi-perturbation and multi-context designs (for example, systematic perturbation of multiple enhancers per gene across diverse cell types). This scope goes beyond what can reasonably be included in the present work, and we estimate that it will take years of work.

A major contribution of this work is the establishment, validation, and benchmarking of a novel quantitative framework that identifies which genes and regulatory regimes are expected to behave differently under such perturbations. In the revised manuscript, we further strengthen this framework through orthogonal validation analyses, including alternative cell-type hierarchy (Supplementary Fig. 3; lines 256-260), cross-species comparisons (Figure 3H,I; Supplementary Fig. 8; lines 432-446), and independent regulatory annotations (Supplementary Fig. 6; lines 339-344), demonstrating that these relationships are robust and reproducible. Together, these analyses provide a solid foundation for future functional studies aimed at testing how regulatory architectures implement complex expression programs.

We now address this perspective in the Discussion (lines 725-729), framing functional perturbation of regulatory elements as an interesting future direction enabled by the framework introduced here.

Their use of expression-based cell-type tree is novel. How would other methods of creating a hierarchy of cell types affect the measurement of tMDL?

We agree that the choice of cell-type hierarchy is central to the interpretation of tMDL, and we appreciate the reviewer for raising this important point. Expression-based cell-type trees have been used in prior work to study relationships among cell types (Kin et al., 2015; Karlsson et al., 2021; Church et al., 2024), and we now cite these precedents in the manuscript (line 255). At the same time, it is important to verify that tMDL does not depend too critically on this specific method used to construct the hierarchy.

To address this point directly, we recomputed tMDL using an independently derived, externally defined cell-type hierarchy that is not based on gene expression. Specifically, we used a chromatin accessibility-based cell-type tree from Zhang et al. (Cell, 2021), constructed from genome-wide single-cell ATAC-seq profiles across human tissues. Because this hierarchy is derived from epigenomic data that are independent of the HPA expression dataset used in our primary analysis, it provides an orthogonal opportunity for testing our original computation and framework.

We performed the analysis on the 34 cell types common to both datasets and recomputed tMDL using this alternative tree. The resulting tMDL values were highly concordant with those obtained using the expression-based hierarchy (Spearman $\rho = 0.95$; Pearson $r = 0.96$; Response Fig. 1A; Supplementary Fig. 3A), indicating that tMDL is largely insensitive to the specific method used to infer the cell-type tree. Notably, because the chromatin-based hierarchy spans fewer cell types than our original tree (34 vs. 81), the absolute scale of tMDL values is reduced, as expected; nonetheless, the strong concordance demonstrates that relative regulatory complexity is preserved across hierarchies.

Importantly, all key downstream relationships were preserved when using the chromatin-based hierarchy, including the “horseshoe” relationship between tau and tMDL, as well as the positive associations between tMDL and multiple independent measures of regulatory complexity including CREs, TFs, miRNAs, and 3' UTR length (Response Fig. 1B-F; Supplementary Fig. 3C-F). Together, these results indicate that tMDL captures stable, biologically meaningful aspects of regulatory demand that are robust to alternative, independently constructed cell-type hierarchies. This analysis is now presented in Supplementary Fig. 3, with corresponding revisions in the main text (lines 256–260).

We thank the referee for this comment, the independent means of calculation increases confidence in the deduced results

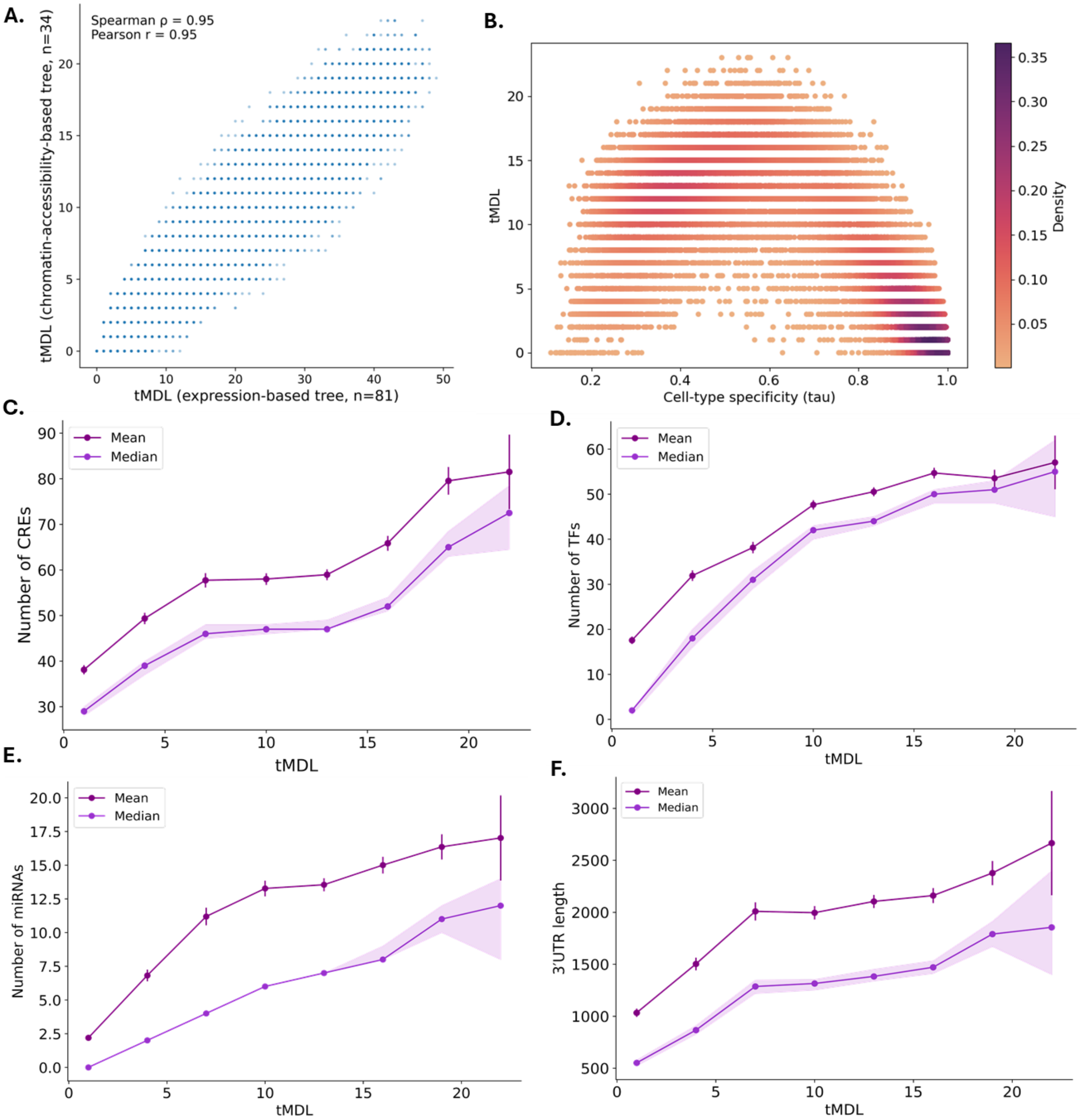

Response Figure 1. Robustness of tMDL to alternative cell-type hierarchies. (Supplementary Fig. 3 in the revised manuscript) (A) Scatterplot comparing gene-level tMDL values computed using expression-based and chromatin accessibility–based cell-type hierarchies. **(B)** Relationship between cell-type specificity (tau) and tMDL using the chromatin-based hierarchy. **(C–F)** Mean and median values of regulatory features per gene plotted across ranked tMDL values, including cis-regulatory element count, TF count, miRNA count, and 3'UTR length.

Does their suggested principle hold across species? Testing this could establish whether MDL is universal in gene regulation.

This is an excellent point, and we share the reviewer's interest in whether the principles captured by tMDL generalize across species. We therefore tested this explicitly, starting with an evolutionarily close system and then extending to a more distant one.

First, we evaluated conservation between human and mouse using a single-study mouse single-cell atlas. Cell-type specificity (tau) exhibited a similar bimodal distribution in mouse and human, with peaks at low and high tau values, indicating comparable separation between broadly expressed and tissue-specific genes across species (Response Fig. 2A-B; Supplementary Fig. 8A). Consistent with this, direct comparison across 10,785 human-mouse orthologs revealed strong correlations (Pearson $r = 0.87$; Spearman $\rho = 0.86$; Response Fig. 2C; Supplementary Fig. 8B). Tissue-specific genes were particularly conserved, with 89% of genes classified as tissue-specific in human (tau > 0.8) also classified as such in mouse. These results indicate that relative expression patterns are stable across species despite differences in tissue coverage and cell-type resolution.

We next examined tMDL. Human and mouse tMDL values across orthologs were positively correlated (Pearson $r = 0.68$; Spearman $\rho = 0.65$; Response Fig. 2D; Supplementary Fig. 8C), indicating that a substantial component of expression-pattern complexity is conserved, albeit with greater variability than tau, as expected given that tMDL captures hierarchical structure rather than expression breadth alone.

Importantly, despite some variability, the same biological relationships were preserved in mice. The tau score exhibited the characteristic inverted U-shaped relationship with 3' UTR length, as well as the monotonic relationship with TF count. In addition, genes with higher tMDL showed increased regulatory complexity, reflected by both longer 3' UTRs and greater numbers of TFs (Response Fig. 2E-F; Supplementary Fig. 8D-E). These patterns closely recapitulate those observed in human data despite differences in species, atlas composition, and regulatory annotations.

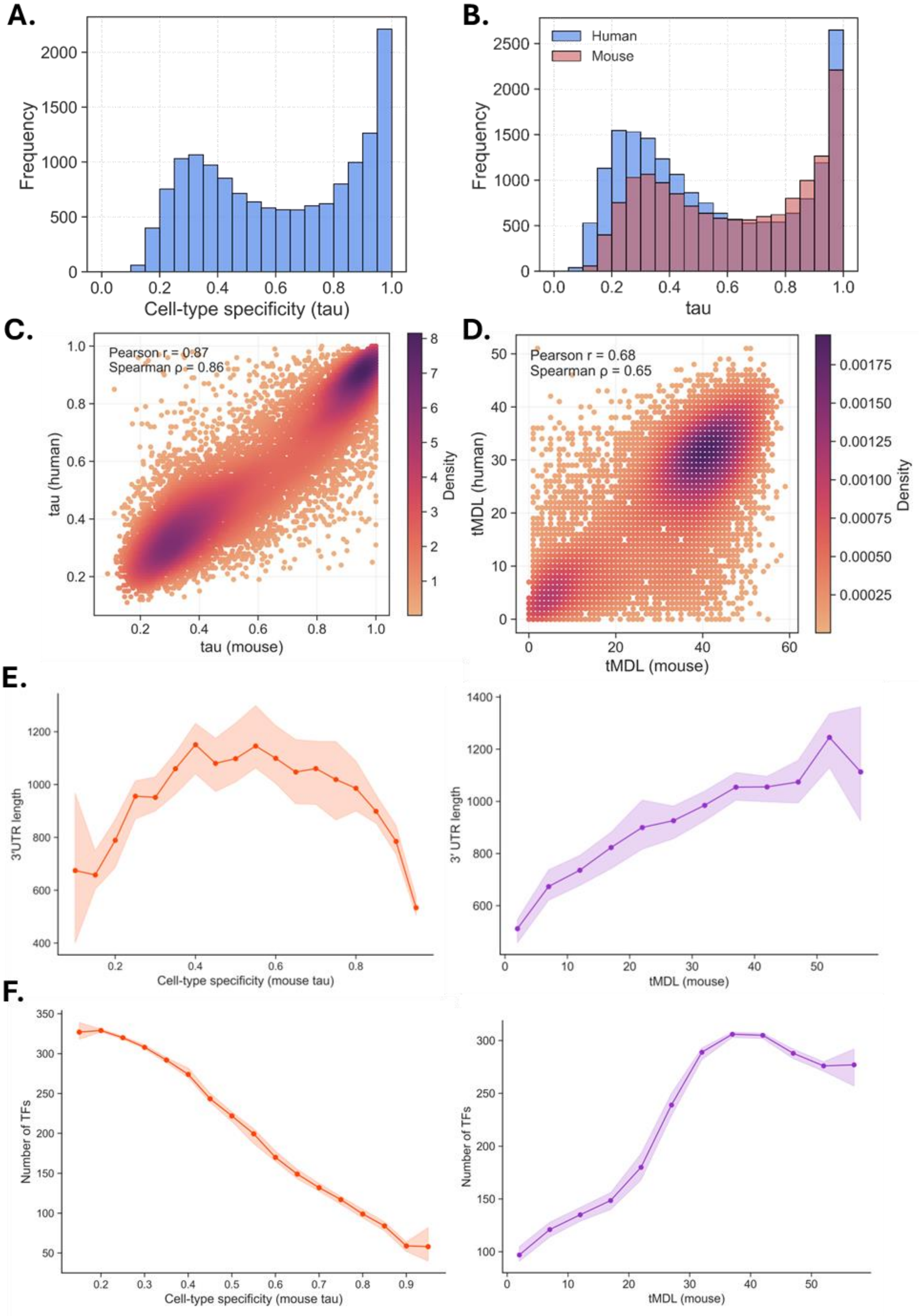
A.
Frequency
Cell-type specificity (tau)
B.
Human
Mouse
Frequency
tau
C.
Pearson r = 0.87
Spearman ρ = 0.86
tau (human)
tau (mouse)
Density
D.
Pearson r = 0.68
Spearman ρ = 0.65
tMDL (human)
tMDL (mouse)
Density
E.
3'UTR length
Cell-type specificity (mouse tau)
3' UTR length
tMDL (mouse)
F.
Number of TFs
Cell-type specificity (mouse tau)
Number of TFs
tMDL (mouse)

127

Response Figure 2. Cross-species robustness of tau, tMDL, and regulatory features assessed in mouse. (Figure 3H and Supplementary Fig. 8 in the revised manuscript) (A) Distribution of tau values for mouse protein-coding genes. **(B)** Overlapping histograms of tau for one-to-one human (blue) and mouse (pink) orthologs. **(C)** Scatterplot comparing mouse and human tau values for orthologous genes. **(D)** Scatterplot comparing mouse and human tMDL values for the same genes. **(E)** Median 3' UTR length and **(F)** median number of regulating TFs per gene plotted as a function of ranked tau (left) or tMDL (right) in mouse.

To test whether these principles extend across larger evolutionary distances, we further analyzed bulk RNA-seq data from 13 tissues in *Drosophila melanogaster* (FlyAtlas). Despite substantial differences in organism, tissue composition, and data modality, we observed the same qualitative patterns: tau showed a bimodal distribution, the characteristic horseshoe-shaped relationship between tau and tMDL was retained, and increasing tMDL was associated with greater regulatory complexity. This complexity was reflected by longer 3' UTRs and higher TF counts, the latter based on regulatory interactions from TFLink (Liska et al., 2022), reproducing the trends observed in mammals (Response Fig. 3A-D; Supplementary Fig. 8F-I). While regulatory annotations in flies are more limited, these results overall mirror the trends observed in mammals.

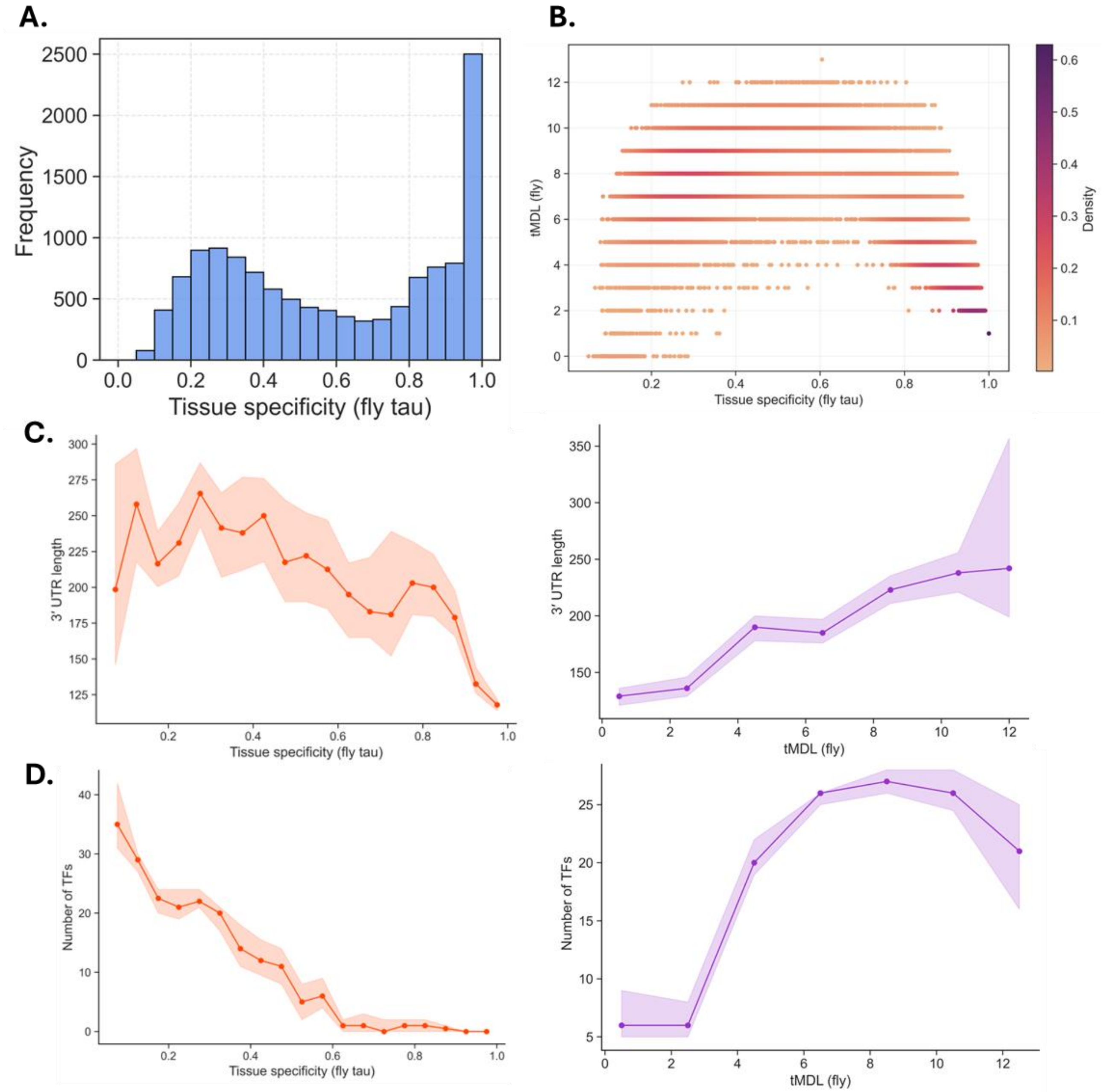

Response Figure 3. Cross-species robustness of tau, tMDL, and regulatory features in *Drosophila*. (Figure 3I and Supplementary Fig. 8 in the revised manuscript) (A) Distribution of tau values for *Drosophila melanogaster* protein-coding genes. **(B)** Scatterplot showing the relationship between tau and tMDL across fly genes, with points colored by local density. **(C)** Median 3' UTR length per gene plotted as a function of ranked tau (left) or tMDL (right). **(D)** Median number of regulating TFs per gene plotted as a function of ranked tau (left) or tMDL (right).

Together, these analyses indicate that the relationship between expression-pattern complexity, tissue specificity, and regulatory architecture captured by tMDL generalizes

from humans to mice and extends to flies, a more evolutionarily distant system. This supports the view that the MDL-consistent organization we describe reflects broad organizing principles of gene regulation rather than species- or dataset-specific effects.

The cross-species validation analyses are now included in the Supplementary Information (Supplementary Fig. 8), with the results regarding 3'UTR scaling in mouse and *Drosophila* incorporated into the main text as Figure 3H–I. The corresponding text describing these results has been added to the main Results section (lines 432-446).

Minor comments:

Would the authors publish code or a tool to calculate tMDL?

We are happy to provide the code used to calculate tMDL. As part of the revised manuscript, we provide Supplementary tables containing the precomputed tMDL values used throughout this study, calculated using the HPA expression data and the expression-derived hierarchical tree described in the Methods. These tables provide readily accessible tMDL values for all human (Supplementary Table 1) and mouse genes (Supplementary Table 2) analyzed here, which we hope will serve as a useful resource.

In addition, we made available a Python script implementing the tMDL calculation as used in this work (Supplementary Code 1). The script operates on an expression matrix together with a hierarchical tree provided in Newick format and computes tMDL via expression discretization followed by Fitch's parsimony algorithm, as described in the Methods. This enables readers to reproduce our analyses using the published inputs or to apply the tMDL framework to alternative datasets, organisms, or hierarchical trees, should they wish to do so.

Reviewer #3

This is an important manuscript about fundamental aspects of gene regulation. I believe it is a very important work that will be foundational to many future studies. Golomb, Pilpel and colleagues decipher the breadth of gene expression according to cis-regulatory elements using principles from information theory. This is a power lens and so the manuscript will represent an important contribution to the literature.

We are very grateful to the reviewer for their encouraging assessment of our work and for recognizing the potential of the tMDL framework to serve as a foundation for future studies in gene regulation.

I just have a few critics that may help the authors in improving the clarity of the work. A main claim of the paper is that (from the abstract) “Two patterns emerged: features scaling with MDL in selectively expressed genes tend to act as on/off switches, whereas those in ubiquitous genes serve as fine-tuning knobs” However it’s not clear if the “knob” versus “switch” dichotomy is seen as a result or an assumption by the authors. In line 409, for example it is stated as an assumption: “These genes are expected to require fine-tuned, quantitative control, hence refer to here as “knob-like” regulation.” I suggest that the authors better define this dichotomy and what evidence tests would be able to falsify the assertion.

This is an important conceptual point, and we agree that the distinction between “knob-like” and “switch-like” regulation should be clearly framed as an interpretive framework rather than as a result.

In the revised manuscript, we clarify that these terms are used as conceptual descriptors of expected regulatory behavior associated with broad versus restricted expression, motivated by a parsimonious regulatory logic. Genes active across all cell types are most efficiently controlled through quantitative tuning of expression levels, whereas genes restricted to fewer tissues and organs are likely more efficiently regulated through discrete activation or repression. Importantly, we do not imply that these modes are mutually exclusive; cell-type-selective genes may still employ some quantitative fine-tuning in addition to switch-like regulation. We have made this framing explicit in the text to prevent the dichotomy from being interpreted as a proven result (lines 455-462).

While we do not functionally validate this distinction here, it is empirically anchored in the sense that its interpretive value depends on whether regulatory features behave differently across expression regimes. If broadly and selectively expressed genes exhibited indistinguishable relationships between regulatory features and expression-pattern complexity, this framework would not be informative. Instead, we observe consistent regime-specific patterns across multiple independent regulatory layers, which motivate, though do not prove, the proposed interpretation.

Moreover, in this analysis the grouping in the Figure 4 analysis is based on expression in <90% of tissues as being selective. But a gene expressed in say 80% of the tissues still sounds as pretty ubiquitous. To make a stronger analysis the authors may choose to examine how their results depend on this cutoff.

We appreciate the reviewer’s point and agree that it is important to assess whether our conclusions depend on the specific cutoff used to define “ubiquitous” versus “cell-type-selective” genes. To address this concern and to avoid reliance on an arbitrary threshold, we tested the sensitivity of our results to alternative thresholds. In addition to the 90% cutoff

used in the main analysis, we repeated the analysis using both a more stringent threshold (≥95% of cell types) and a more permissive threshold (≥80% of cell types) to define ubiquitous expression.

The qualitative results were highly consistent across all tested cutoffs (Response Fig. 4; Supplementary Fig. 9), indicating that the observed differences between regulatory regimes are not driven by the specific threshold chosen. We have added these sensitivity analyses to the Supplementary material (Supplementary Fig. 9) and note this robustness in the main text (lines 465-468).

A. Ubiquitous genes = expressed in >**95%** cell types

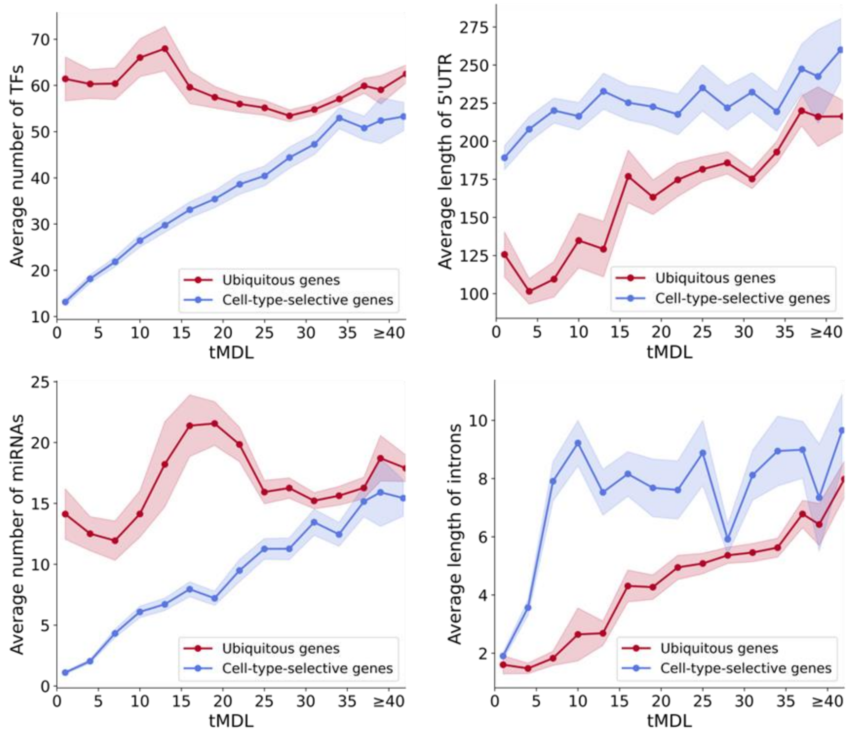

B. Ubiquitous genes = expressed in >**90%** cell types

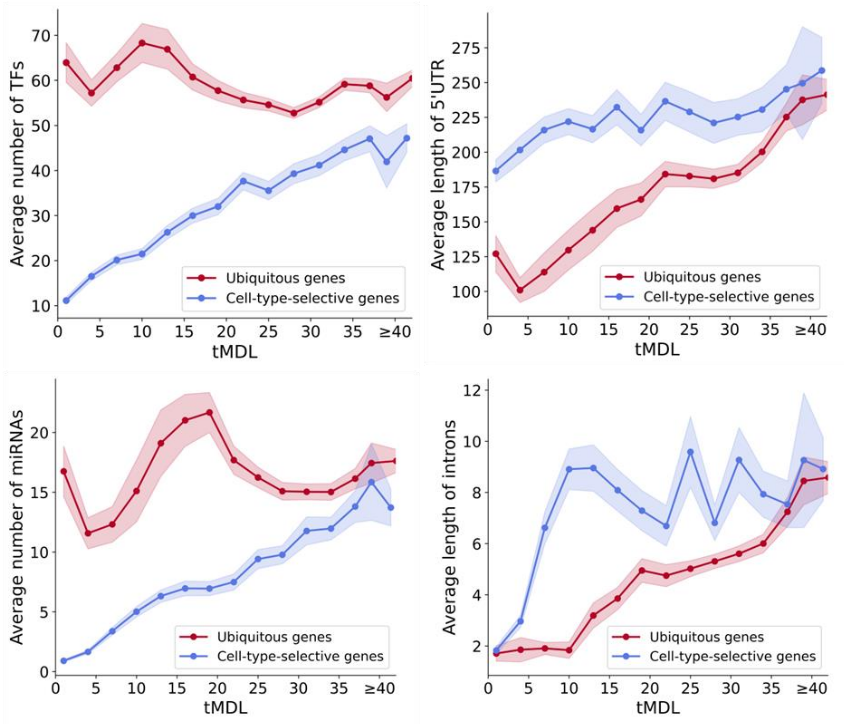

C. Ubiquitous genes = expressed in >**80%** cell types

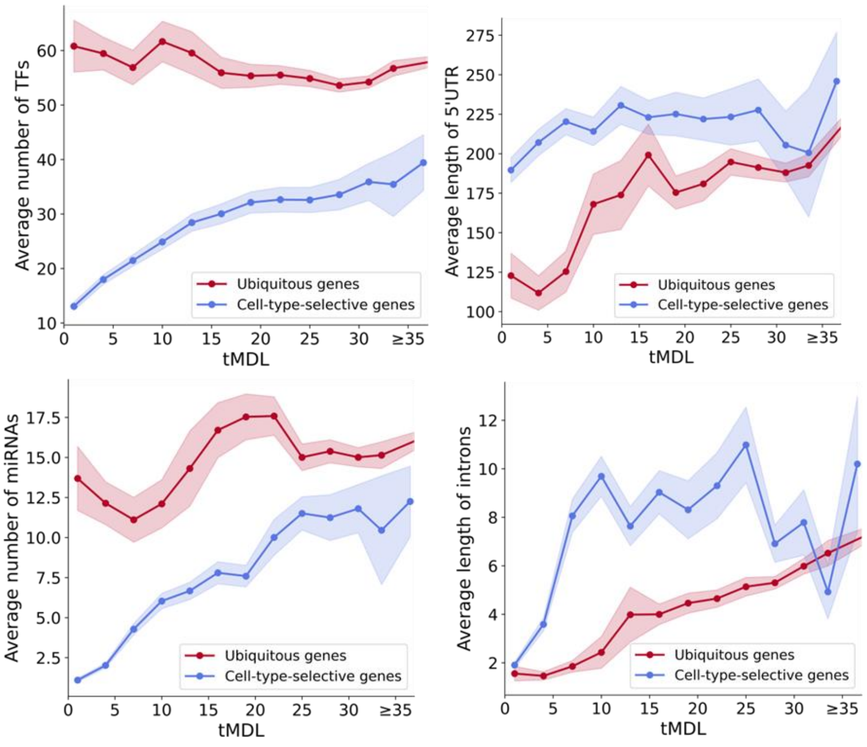

Response Figure 4. Robustness of knob- and switch-like regulatory regimes to the definition of ubiquitous expression. (Supplementary Fig. 9 in the revised manuscript) Mean regulatory feature values plotted across

ranked tMDL for ubiquitous (red) and cell-type-selective (blue) genes using alternative thresholds to define ubiquitous expression. **(A)** ≥95% of tissues, **(B)** ≥90% of tissues (used in the main text), and **(C)** ≥80% of tissues. Features shown: number of TFs, number of miRNAs, 5' UTR length, and intron length.

In Line 330 the authors write: “Interestingly, this increase in TF count plateaued around tau ≈ 0.4, suggesting that once a gene is widely expressed across all tissues, additional TFs provide diminishing returns.” It’s not clear how the graph in Figure 3A supports this assertion since there are not significantly more TFs in genes with tau<0.4.

We thank the reviewer for pointing out this ambiguity. The original phrasing was unclear and could be misinterpreted when reading Figure 3A from low to high tau values. We have revised the text to more accurately describe the observed pattern: TF counts are high and relatively constant among broadly expressed genes (tau < ~0.4) and decline progressively as tau increases beyond this point. This indicates that TF number primarily scales with expression breadth, and that once genes are broadly expressed across tissues, variation in expression levels is not accompanied by further increases in TF count. This clarification avoids implying an increase in TF number within the broadly expressed regime and more accurately reflects the trend shown in Figure 3A (lines 353-357).

Also the authors should explain what would explain why the more broadly expressed genes would be targeted by the most TFs.

We have expanded the text to more clearly explain why, in our understanding, broadly expressed genes are targeted by a larger number of transcription factors. Specifically, we now clarify that because different tissues deploy distinct transcription factor repertoires, genes that are expressed across many tissues are expected to be compatible with a broader range of TF environments, resulting in a larger set of observed TF-target interactions. This explanation has been added to the Results section to provide clearer biological intuition for the observed pattern (lines 357-361).

Figure 4 is a bit difficult to understand and I’m wondering if the authors can show the analysis in a better way. For example, does it really make sense to talk about slope in this analysis?

We thank the reviewer for this comment, which drew our attention to aspects of Figure 4 that were not as intuitive as they should be. We agree that the slope-based representation did not add substantial insight in this context, and we have therefore removed it in the revised figure.

In addition, we realized that the intent of Figure 4A, namely, to define and visualize the two gene groups (ubiquitous versus cell-type-selective), was not sufficiently clear. We have updated this panel to more explicitly convey the group definitions and to ensure that the

color coding used in subsequent panels (blue and red) is unambiguous and clearly tied to these categories.

We believe these changes improve the clarity and interpretability of Figure 4 (Response Fig. 5), and we thank the reviewer for helping us refine its presentation.

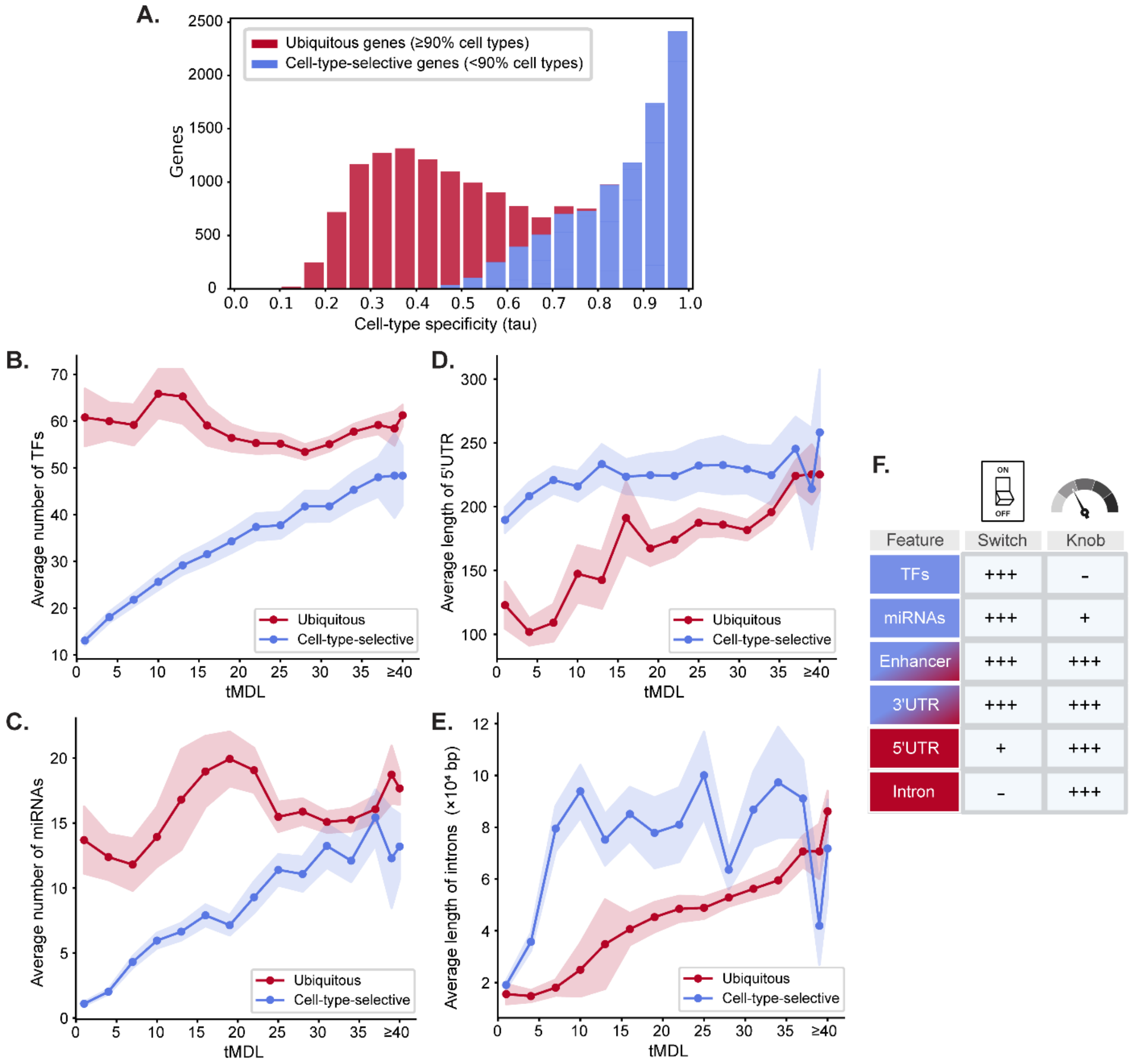

Response Figure 5. Clarified presentation of regulatory regimes in ubiquitous versus cell-type-selective genes. (Figure 4 in the revised manuscript) (A) Distribution of cell-type specificity (tau), highlighting the definition of ubiquitous (≥90% of cell types; red) and cell-type-selective (<90%; blue) gene groups used throughout the analysis. **(B–E)** Mean regulatory feature values plotted across ranked tMDL for ubiquitous (red) and cell-type-selective (blue) genes, computed in fixed-sized windows; shaded regions indicate 90% confidence intervals. Features shown: number of TFs, number of miRNAs, 5' UTR length, and intron length. **(F)**

Summary table indicating whether each feature exhibits switch-like or knob-like scaling with tMDL in the two gene groups.

One minor point; Figure 1B could also indicate the tMDL score.

We agree that indicating the tMDL score in Figure 1B would be informative. However, Figure 1 is designed to introduce tissue/cell-type specificity (tau) prior to the formal definition of tMDL, and including tMDL at this stage would require introducing the concept earlier than intended. We therefore kept Figure 1 focused on tau and present tMDL explicitly starting in Figure 2, where it is defined and interpreted in detail. To address the reviewer's point, we have now highlighted all four example genes from Figure 1B in Figure 2C, allowing readers to see where these genes fall along the tMDL axis once the measure has been introduced.

Reviewer #4

Golomb et al. analyze tissue specificity (T) and a tree-aware MDL metric (tMDL), relating both to regulatory features (CREs, TFs, miRNAs, gene age). The core message, that regulatory demand peaks for genes with intermediate specificity, is interesting and worth publishing. But key analyses lean heavily on aggregate resources and author-defined hierarchies. Several conclusions could be artifacts of dataset choice or construction of the cell-type tree. Below are concrete ways to make this paper truly robust.

We thank the reviewer for recognizing the value of our core message and for stating that it is worth publishing. We appreciate the constructive suggestions for additional robustness tests; we believe these analyses have helped us further strengthen the manuscript and demonstrate the stability of the tMDL framework.

Major concerns

1. Dependence on aggregate databases → request: validate with single-cell atlases (mouse + human).

The tissue-specificity results hinge on HPA (bulk and aggregated scRNA-seq) and compiled cCRE sets. Please replicate the T and tMDL findings using one or more internally consistent single-study atlases with many cell types, ideally both species. For example, you could use the Qiu et al. mouse time series data, and pseudobulk RNA-seq profiles (or other equivalently diverse datasets that are based on a single single cell study w/ very diverse cell types rather than aggregate). Is T similarly distributed? Are gene ranks stable by HPA vs. other datasets? Are specificity classifications of individual genes stable? Are these values and classifications also preserved across mouse vs. human?

In response to the reviewer's request to assess whether reliance on aggregated expression resources influences measures of cell-type specificity or tMDL, we performed an independent validation using Tabula Muris, a high-quality single-study mouse single-cell atlas profiling a broad range of tissues and cell types under a consistent experimental and computational pipeline (The Tabula Muris Consortium, 2018). This dataset provides an internally coherent reference for testing whether the patterns we observe persist outside the HPA dataset and extend across species.

We first asked whether cell-type specificity behaves similarly in the single-study mouse atlas to our original human data. The overall distributions of tau in mouse and human show strong overlap (Response Fig. 2A-B; Supplementary Fig. 8A). While the mouse distribution exhibits a modest reduction in extremely low tau values, the overall shape and dynamic range are highly similar.

Direct comparison of tau values across 10,785 human-mouse orthologs reveals a strong correlation (Pearson's r=0.87; Spearman ρ = 0.86; Response Fig. 2C; Supplementary Fig. 8B), indicating that relative cell-type specificity is highly conserved across species and robust to dataset origin. Tissue-specific genes show particularly high concordance: 89% of genes classified as tissue-specific in human (tau > 0.8) are also tissue-specific in mouse. Together, these results demonstrate that tau is stable across species even when computed from a single-study single-cell atlas with different tissue coverage and resolution.

We next evaluated tMDL. Across orthologs, human and mouse tMDL values are positively correlated (Pearson's r=0.68, Spearman ρ = 0.65; Response Fig. 2D; Supplementary Fig. 8C), indicating that a substantial component of expression-pattern complexity is conserved across datasets, though with greater variability than observed for tau. This difference is not unexpected, as tMDL captures hierarchical structure in expression patterns rather than expression breadth alone.

As we aim to avoid reliance on arbitrary classification thresholds, we additionally tested conservation of tMDL using a rank-based approach focused on the extremes of the distribution. Genes were ranked by scaled tMDL within each species, and the lowest and highest 20% were defined as low and high tMDL tails, respectively. We then tested whether genes classified in the lowest and highest tMDL groups in human were enriched among the corresponding low and high tMDL groups in mouse using Fisher's exact tests. Genes in the lowest tMDL tail in human were strongly enriched among the lowest tMDL tail in mouse (odds ratio = 14.7, $P<10^{-300}$), indicating robust conservation of minimal regulatory complexity. Genes in the highest tMDL tail also showed significant enrichment (odds ratio = 5.5, $P < 10^{-220}$), demonstrating that genes with highly complex expression programs are preferentially retained at the upper end of the tMDL spectrum across species.

While there is general agreement, the observed differences may be influenced by known distinctions between the datasets. The mouse atlas differs from the HPA in both tissue coverage and cell-type resolution: for example, it does not include reproductive tissues such as testis, which exhibit highly distinctive expression programs, while providing finer subdivision of immune populations, including multiple T-cell subtypes where the HPA represents a single T-cell cell type. Such differences inevitably lead to modest shifts in gene-level scores. Nevertheless, despite differences in species, tissues profiled, cell-type definitions, and experimental origin, we observe clear overall agreement in both tau and tMDL across datasets.

Not only do we consistently observe similar gene rankings and classification structure, we find the same biological relationships between tau/tMDL and regulatory features. Strikingly, analyses of the single-study mouse atlas recapitulate the same regulatory relationships observed in human data: tau exhibits the characteristic inverted U-shaped relationship with 3'UTR length, and the number of TFs linked to genes increases monotonically as tau decreases (Response Fig. E-F; Supplementary Fig. 8D-E). In parallel, tMDL shows the same associations as in human, with higher tMDL corresponding to increased regulatory complexity, reflected by longer 3'UTRs and a greater number of TFs (Response Fig. E-F; Supplementary Fig. 8D-E). These concordant patterns are observed despite differences in species, data modality, and regulatory annotations.

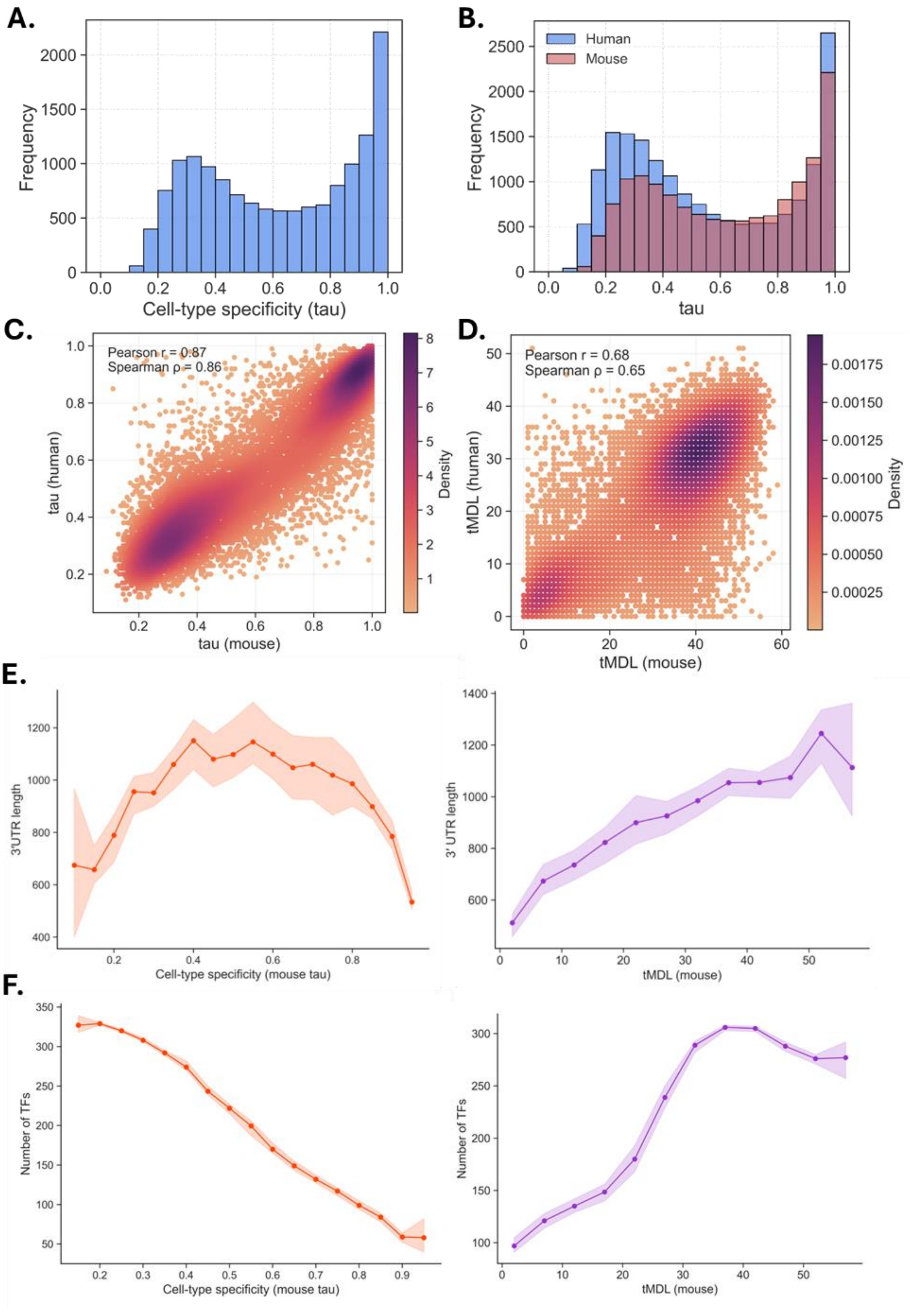
A.
Frequency
Cell-type specificity (tau)
B.
Human
Mouse
Frequency
tau
C.
Pearson r = 0.87
Spearman ρ = 0.86
tau (human)
tau (mouse)
Density
D.
Pearson r = 0.68
Spearman ρ = 0.65
tMDL (human)
tMDL (mouse)
Density
E.
3'UTR length
Cell-type specificity (mouse tau)
3' UTR length
tMDL (mouse)
F.
Number of TFs
Cell-type specificity (mouse tau)
Number of TFs
tMDL (mouse)

Response Figure 2. Cross-species robustness of tau, tMDL, and regulatory features assessed in mouse. (Figure 3H and Supplementary Fig. 8 in the revised manuscript) *Repeated from the analysis presented in response to Reviewer 1 (page 6).* **(A)** Distribution of tau values for mouse protein-coding genes. **(B)** Overlapping histograms of tau for one-to-one human (blue) and mouse (pink) orthologs. **(C)** Scatterplot comparing mouse and human tau values for orthologous genes. **(D)** Scatterplot comparing mouse and human tMDL values for the same genes. **(E)** Median 3' UTR length and **(F)** median number of regulating TFs per gene plotted as a function of ranked tau (left) or tMDL (right) in mouse.

In response to a related comment from another reviewer regarding the generality of these relationships across evolutionary distance, we further asked whether the same patterns are observed in a more distantly related organism (full analysis on pages 7-8 of this response). Using bulk RNA-seq data from 13 tissues in *Drosophila melanogaster* (FlyAtlas), we computed tau and tMDL scores using the same framework. Despite substantial differences in organism, tissue composition, and data modality, we observed the same qualitative patterns: tau exhibited a bimodal distribution of tissue specificity (Response Fig. 6A; Supplementary Fig. 8F), and increasing tMDL was associated with greater regulatory complexity, reflected by longer 3'UTRs and a higher number of linked TFs (Response Fig 6B-C; Supplementary Fig. 8H-I).

The recurrence of these relationships in an evolutionarily distant system like *D. melanogaster* supports the idea that the links between expression-pattern complexity, tissue specificity, and regulatory architecture are shared across species. We note that while gene regulatory data of similar breadth and quality to human resources are naturally more limited in other model species, the emergence of the same trends indicates that the tMDL framework reflects a general principle of gene regulation rather than a species- or dataset-specific phenomenon.

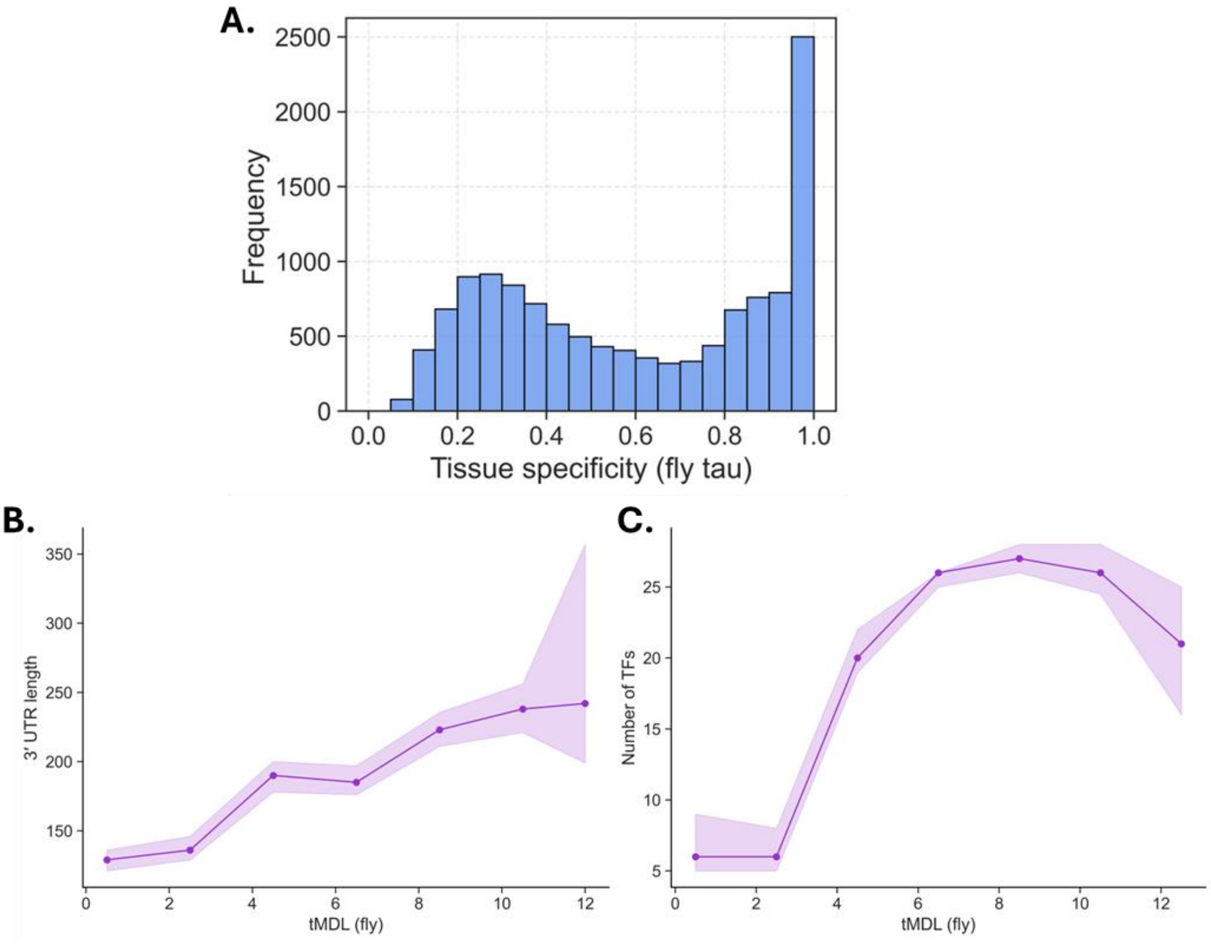

Response Figure 6. Robustness of regulatory scaling in *Drosophila melanogaster*. (Figure 3I and Supplementary Fig. 8 in the revised manuscript; Extracted from the full cross-species analysis in Response Figure 3). (A) Distribution of tau values for fly protein-coding genes. **(B)** Median 3' UTR length and **(C)** median number of regulating TFs per gene plotted as a function of ranked tMDL in flies.

Thus, while exact gene-level values are not fully identical across datasets, as perhaps expected given differences in species and atlas composition, the relative organization of genes along the tau and tMDL axes, and their relationship to regulatory features, is preserved. This concordance across independent datasets indicates that tau and tMDL capture robust, biologically meaningful properties of gene regulation rather than dataset-specific artifacts.

These validation analyses are now included in the Supplementary Information (Supplementary Fig. 8), with the results regarding 3'UTR scaling in mouse and *Drosophila* incorporated into the main text as Figure 3H–I. The corresponding text describing these results has been added to the main Results section (lines 432-446).

2. cCRE counts may mirror sampling/aggregation bias → request: add orthogonal CRE baselines based on single studies. The inverted-U in CRE counts could reflect what's been profiled, e.g. aggregation artifact. Please add either or both of two orthogonal validations: 1) Single-study regulatory maps (similar point, you don't have to use these particular studies but they are just examples -- human: e.g., Domcke-style; mouse: Cusanovich-style or equivalent). Ideally redo CRE-per-gene vs τ and vs tMDL from scratch with the CREs/links defined within one study. 2) Conservation-only enhancer prior (no biochemical data): use noncoding conservation "pockets" matching enhancer-like sequence features and link by nearest-gene/ABC-style proximity as a sensitivity check.

We thank the reviewer for this insightful suggestion. We agree that ensuring findings remain robust across different data aggregation strategies is a critical step in large-scale genomic analyses. To address this, we performed both suggested orthogonal validation analyses to ensure our findings reflect a robust biological signal. The first test we did was using a landmark single-study regulatory atlas (mouse scATAC-seq from Cusanovich et al., 2018) and the second was using a conservation-only enhancer baseline (PhastCons).

1. Single-study validation: Mouse scATAC-seq (Cusanovich et al., 2018)

To assess whether our findings are robust to the source of the regulatory map, we utilized the comprehensive mouse single-cell chromatin accessibility atlas (Cusanovich et al., 2018). We focused on the Cicero-derived co-accessibility maps, which provide an empirically defined link between distal regulatory elements and target genes based on chromatin dynamics across ~100,000 cells and 13 tissues.

We relied on the Cicero-derived co-accessibility framework to define enhancer-gene associations, following the authors' thresholds: (i) distal sites with co-accessibility > 0.2 to a proximal site, and (ii) for remaining unlinked sites, the strongest link with co-accessibility > 0.1.

Results and Observations:

The relationship between CRE count and tMDL is strongly recapitulated in this single-study atlas: genes with higher tMDL values are consistently associated with a greater number of linked CREs, in agreement with our primary analysis (Response Fig. 7A; Supplementary Fig. 6B). This indicates that the association between regulatory burden and expression-pattern complexity is robust across species and independent of the specific regulatory map used.

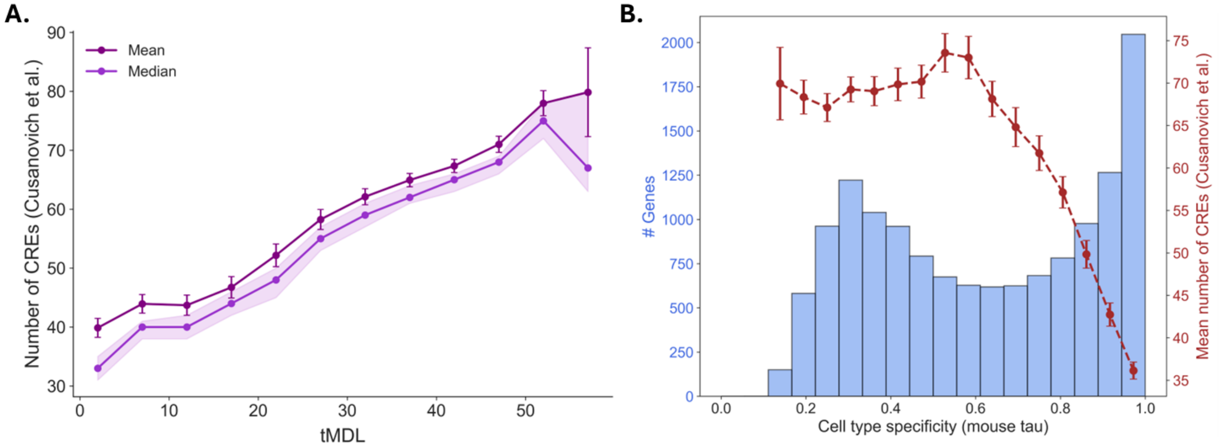

Response Figure 7. Robustness of CRE-based scaling using a single-study regulatory atlas. (Supplementary Fig. 6 in the revised manuscript). CRE–gene associations were derived from a single scATAC-seq study (Cusanovich et al., 2018) using Cicero co-accessibility links. **(A)** Mean (purple) and median (violet) number of CREs per gene plotted as a function of ranked tMDL values. **(B)** Distribution of tau scores (blue bars) overlaid with the mean number of CREs per gene within each tau bin (red dashed line).

The relationship between CRE count and tau shows an inverted-U shape, similar to the one in human, although more asymmetrical and attenuated compared to our main analysis (Response Fig. 7B; Supplementary Fig. 6A). Genes with intermediate tau still exhibit the highest CRE counts and tissue-specific genes (high tau) consistently show the lowest regulatory burden, as seen in our previous results. However, the decrease in CRE counts for broadly expressed genes (low tau) is less pronounced in the scATAC-seq–based analysis.

We believe this difference may reflect inherent properties of co-accessibility-based regulatory inference in single-cell ATAC-seq data. Single-cell ATAC-seq based co-accessibility models are sensitive to the prevalence of chromatin accessible regions across cells. Regulatory elements associated with broadly expressed genes are observed in many cells and thus benefit from higher statistical power for detecting co-accessibility, whereas regulatory links for genes with restricted, variable, or context-specific activity are more likely to be missed. As a result, CRE counts derived from scATAC atlases may partially reflect detection frequency in addition to regulatory demand. Notably, despite this sensitivity at the low-tau end, the relationship between CRE count and tMDL is fully preserved. This is consistent with tMDL quantifying the complexity of a gene's expression pattern across the expression hierarchy, rather than expression breadth, and indicates that the tMDL-CRE association is not primarily driven by biases related to methodology, the data source or expression prevalence.

The results of this single-study validation are now included in the Supplementary Information (Supplementary Fig. 6), with corresponding discussion added to the main Results section (lines 339–344).

2. Conservation-only CRE baseline (PhastCons)

As an additional sensitivity analysis, we constructed a conservation-only enhancer prior that is completely independent of biochemical assays. We identified Conserved Noncoding Elements (CNEEs) by extracting PhastCons "pockets" of high vertebrate conservation, explicitly excluding all coding sequences (exons). These CNEEs were then linked to all transcription start sites within a 100kb regulatory domain; this 'ABC-style' proximity approach can capture distal interactions that are often missed by nearest-gene assignments while remaining independent of biochemical sampling bias.

Results and Observations:

Even under this biochemically blind definition of regulatory elements which captures only a deeply conserved subset of regulatory sequences, we retain a positive association between CRE count and tMDL: genes with higher expression-pattern complexity are linked to a greater number of conserved noncoding elements (Response Fig. 8A). The relationship between CRE count and tau again exhibits a peak at intermediate tau values, with tissue-specific genes showing the lowest CRE counts (Response Fig. 8B). As in the scATAC-seq analysis, differences relative to the aggregated data are most evident for broadly expressed genes, although the decrease at low tau is moderately more pronounced than in the single-study atlas.

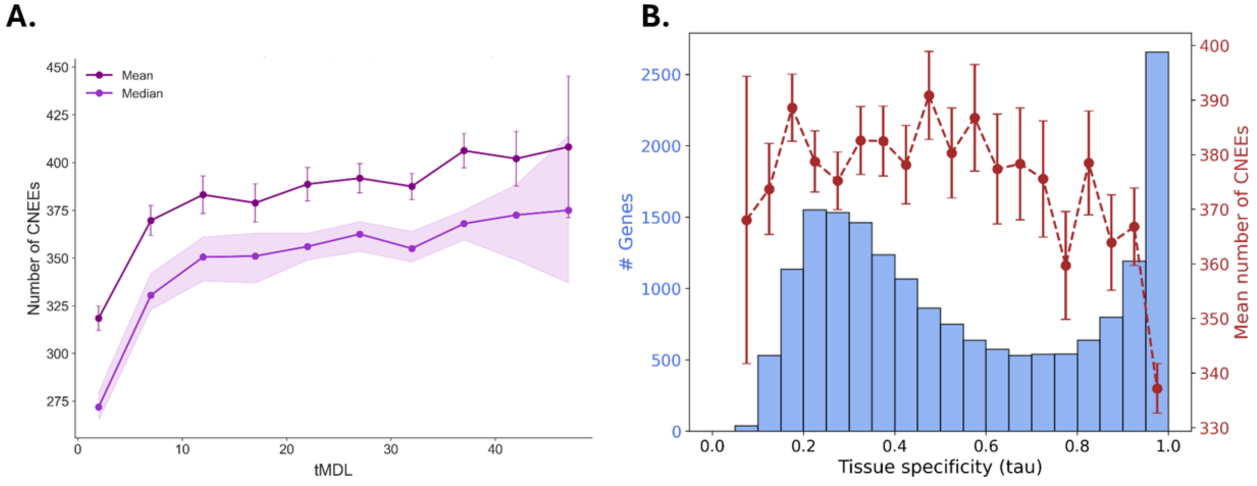

Response Figure 8. Robustness of CRE-based scaling using a using a conservation-only (biochemically blind) CRE baseline. We identified Conserved Noncoding Elements (CNEEs) using vertebrate PhastCons scores, excluding all coding exons. CNEEs were linked to genes within a 100kb regulatory domain. **(A)** Mean

(purple) and median (violet) number of CNEEs per gene plotted as a function of ranked tMDL values. **(B)** Distribution of tau scores (blue bars) overlaid with the mean number of CNEEs per gene within each tau bin (red dashed line).

It is worth noting that CNEEs derived from PhastCons represent any functionally constrained noncoding sequence and are therefore not limited to regulatory elements, but may also include other conserved features such as noncoding RNAs and other structural regions. These conserved elements are often fragmented into small pockets, such that multiple CNEEs can fall within a single regulatory region, inflating the apparent number of elements per gene. In addition, because gene assignment in this analysis relies on genomic proximity rather than biochemical evidence, CNEEs are simply linked to nearby genes, further increasing variance in the estimates and introducing uncertainty in target assignment. Consistent with these properties, this analysis exhibits substantially larger error bars than CRE counts derived from biochemical annotations. Nevertheless, despite these sources of noise, the overall structure of the signal is preserved, supporting the robustness of our main conclusions.

Our primary cCRE analysis intentionally leverages an aggregated regulatory resource that integrates multiple experimental modalities and evidence types. While aggregation can raise concerns regarding technical bias, it also mitigates biases intrinsic to any single assay or inference strategy by reducing reliance on one measurement modality that may preferentially detect regulatory elements in specific regimes. In this sense, aggregation provides a more balanced estimate of regulatory burden across genes with different expression patterns. Supporting this approach, in our original analysis we found that our results are fully recapitulated when restricting the analysis to 'Double Elite' GeneHancer elements, a high-confidence subset requiring at least 2 independent sources for both the element’s existence and its gene linkage (Response Fig. 9; Supplementary Fig. 2). Although this significantly reduces the number of analyzed elements, the retention of the core scaling relationship emphasizes that the signal is driven by high-confidence regulatory architecture. Incorporating the orthogonal validations suggested by the reviewer was valuable and strengthened the analysis by explicitly testing whether our conclusions depend on this aggregation.

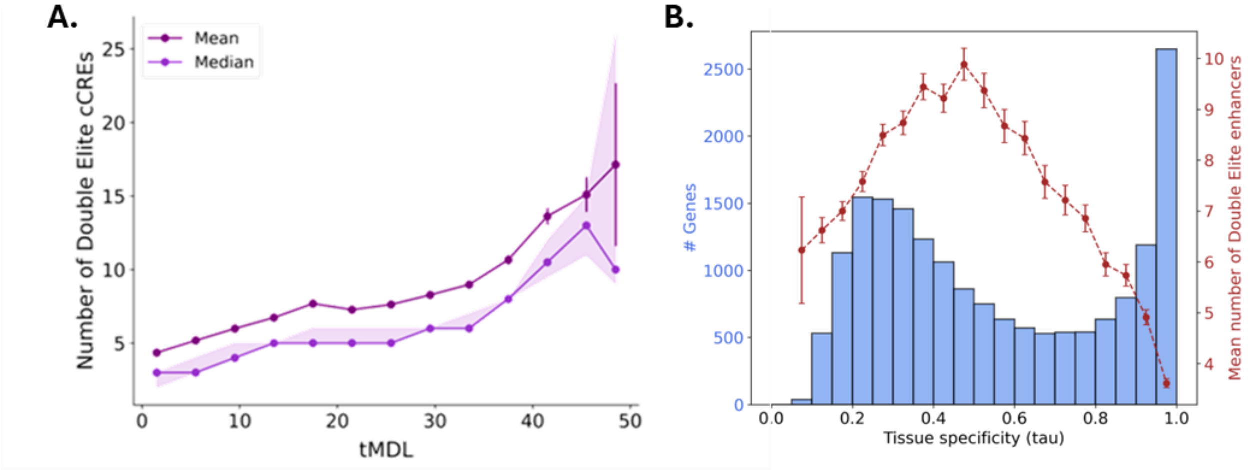

Response Figure 9. Robustness of CRE-based scaling using the high-confidence “Double Elite” GeneHancer subset. (Extracted from Supplementary Fig. 2). (A) Mean (purple) and median (violet) number of Double Elite cCREs per gene plotted as a function of ranked tMDL values. **(B)** Distribution of tau scores (blue bars) overlaid with the mean number of Double Elite cCREs per gene within each tau bin (red dashed line).

Taken together, these analyses show that the overall structure of the relationship between CRE count and tMDL is preserved across fundamentally different regulatory definitions. Across both a single-study scATAC-seq atlas, a conservation-only enhancer prior, and a high-stringency "Double Elite" aggregate, genes with intermediate tau consistently exhibit the highest CRE counts, while tissue-specific genes (high tau) show the lowest regulatory burden. While the exact shape of the tau-based relationship is somewhat sensitive to the underlying detection and linkage framework (particularly for broadly expressed genes), the association between CRE count and tMDL remains remarkably consistent across all baselines. These results reinforce our central conclusion that tMDL captures a fundamental aspect of regulatory demand related to expression-pattern complexity rather than expression breadth.

3. tMDL may depend on how you built the cell-type hierarchy → request: also try to use external hierarchies.

tMDL is only as credible as the tree, which you built. Can you try using at least one external, pre-defined cell-type ontology/hierarchy (e.g., from the atlas itself, e.g. Qiu 2024 of mouse embryogenesis) to recompute tMDL and show the main results are stable?

We agree with the reviewer that the interpretability of tMDL depends on the robustness of the underlying cell-type hierarchy. To address this concern, we recomputed tMDL using an

independent, externally defined cell-type hierarchy, rather than one inferred from expression similarity.

Specifically, we used the chromatin accessibility-based cell-type tree from Zhang et al. (Cell, 2021), which was constructed using genome-wide scATAC-seq profiles across human tissues. This hierarchy is derived from epigenomic data that are entirely independent of the HPA expression dataset, and therefore provide an orthogonal validation of our framework. From this tree, we selected the subset of 34 cell types that overlapped with those profiled in our HPA expression dataset. Using the same gene expression data as in our primary analysis, we then recomputed tMDL for all genes using this alternative, externally defined topology.

Reassuringly, the resulting tMDL values were highly consistent with those obtained using our expression-based hierarchy: the two tMDL estimates showed a Spearman correlation of 0.95 and a Pearson correlation of 0.95 across genes (Response Fig. 1A; Supplementary Fig. 3A). Notably, because the chromatin-based hierarchy spans fewer cell types than our original tree (34 vs. 81), the absolute range of tMDL values is correspondingly reduced, as expected; nevertheless, the strong agreement indicates that relative regulatory complexity is preserved across hierarchies. This strong concordance indicates that tMDL is largely insensitive to the specific method used to construct the hierarchy, and instead reflects stable, underlying patterns of regulatory demand across cell types. Because chromatin accessibility and gene expression capture related, though distinct, layers of gene regulation, such agreement across independently derived cell-type trees is expected if both hierarchies reflect true underlying biology; the observed concordance therefore supports the biological validity of tMDL.

Importantly, all key downstream results were preserved when using the chromatin-based tree. In particular, (i) the characteristic “horseshoe-shaped” relationship between tau expression specificity and tMDL remained unchanged (Response Fig. 1B; Supplementary Fig. 3B), and (ii) the positive trend between tMDL and multiple independent measures of regulatory complexity, including the number of CREs, TFs, and miRNAs, and 3'UTR length, were fully retained (Response Fig. 1C-F; Supplementary Fig. 3C-F).

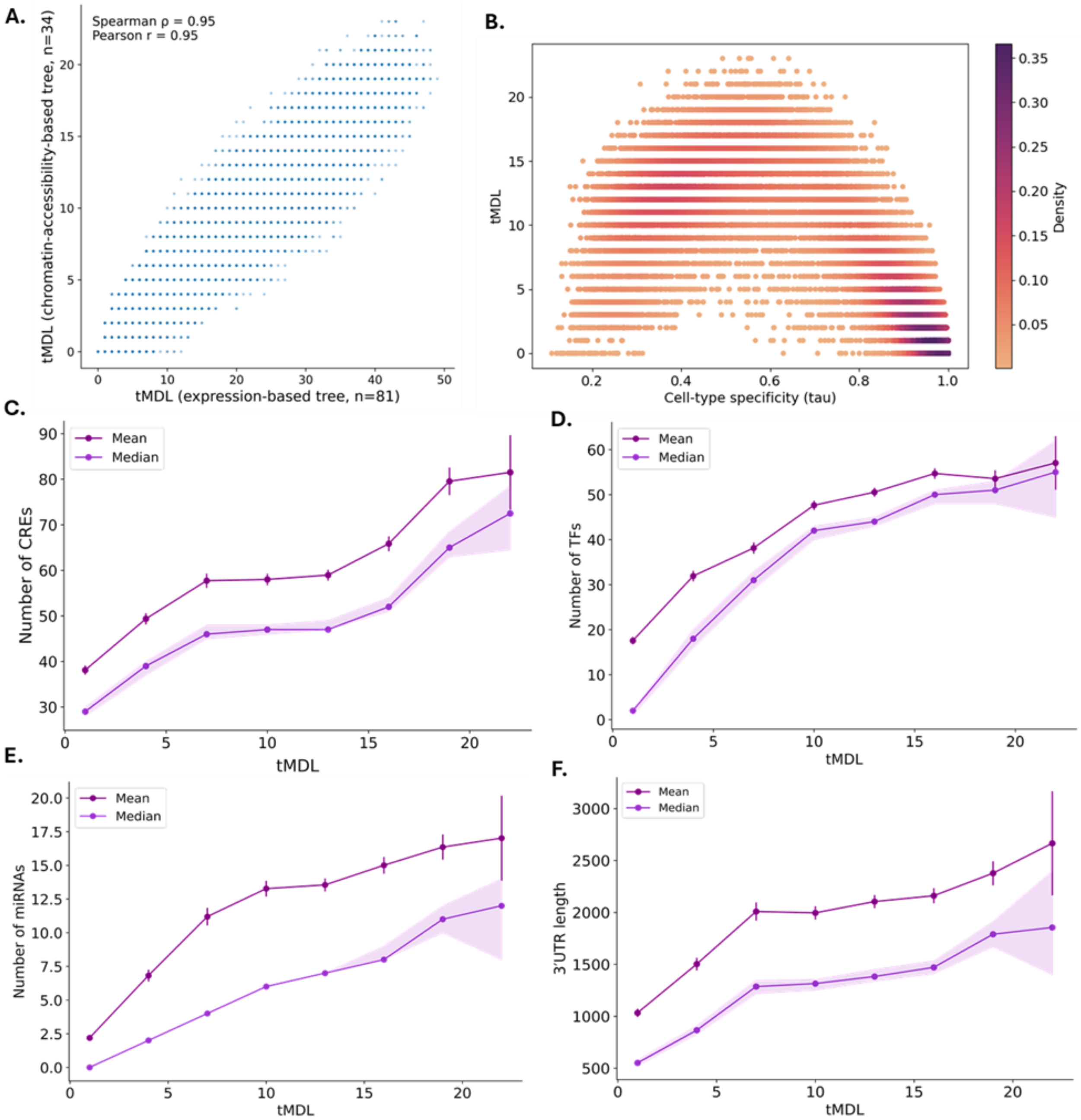

Response Figure 1. Robustness of tMDL to alternative cell-type hierarchies. (Supplementary Fig. 3 in the revised manuscript) *Repeated from the analysis presented in response to Reviewer 1 (page 4)*. **(A)** Scatterplot comparing gene-level tMDL values computed using expression-based and chromatin accessibility–based cell-type hierarchies. **(B)** Relationship between cell-type specificity (tau) and tMDL using the chromatin-based hierarchy. **(C–F)** Mean and median values of regulatory features per gene plotted across ranked tMDL values, including cis-regulatory element count, TF count, miRNA count, and 3'UTR length.

Together, these analyses demonstrate that our conclusions do not depend on a particular hierarchy construction strategy. Instead, tMDL captures a robust property of gene regulation that is consistent across independently derived, biologically meaningful cell-type hierarchies.

The figures corresponding to this alternative hierarchy analysis are now included in the Supplementary Information (Supplementary Fig. 3; lines 256-259).

4. I may have missed this, but a more nuanced analysis of the gene age in relation to "very high" vs. "very low' vs. "intermediate" Tau would be helpful. I might expect ancient genes to be overwhelmingly housekeeping, while genes universal to metazoans might have high Tau/low MDL, and then mammalian specific genes (e.g. TFs with complex roles) to be more intermediate and have larger regulatory footprints. I think that this section could be presented in a much more interesting way than the current GO analyses, etc. For example, pointing out specific developmental TFs that have large (or small) footprints and complex (or simple) expression patterns, relating that to their age, etc. This is listed as a major comment but I might categorize it more as a 'missed opportunity'...

We appreciate the reviewer's suggestion to further develop the evolutionary age analysis and to more clearly articulate how gene age relates to tissue specificity (tau), tMDL, and regulatory architecture. We agree that this section was not presented clearly enough in the original manuscript, and have revised the text and the main figure to sharpen the central message that the relationship between tau, tMDL, and regulatory architecture depends on evolutionary age in a structured manner.

The reviewer raises an intuitive expectation that evolutionary age should structure gene expression, such that ancient genes are predominantly housekeeping, genes arising in early metazoans show increased tissue specificity with comparatively simple regulatory programs, and younger genes exhibit more intermediate expression patterns. In our original Fig. 6A, we examine this by asking how tissue specificity (tau) varies across evolutionary age. Consistent with this intuition, ancient genes are overwhelmingly broadly expressed and exhibit low tau values characteristic of housekeeping functions. Beyond this, however, the data reveal a more monotonic pattern: genes of intermediate evolutionary age tend to exhibit more intermediate tau values, whereas the most recent genes are highly tissue-specific (high tau). We have now made this result clearer in the revised text (lines 577-580).

To better highlight how evolutionary age modulates regulatory demand, we also revised the primary analysis presented in Figure 6C (Response Fig. 10). In the original version, this panel showed the slope of the relationship between tMDL and CRE number across tau bins, with

the aim of conveying that higher expression-pattern complexity is associated with increased regulatory content, particularly among genes with intermediate tau and age. We recognize that this representation was not sufficiently intuitive or direct. We therefore replaced it with a heatmap that bins genes explicitly by tMDL (rather than tau), using the same five evolutionary age groups and four tMDL bins, and colors each bin by the mean number of CREs.

This revised visualization clarifies two related points. First, across all evolutionary age groups, mean cCRE abundance increases with tMDL, indicating that the scaling between expression-pattern complexity and regulatory content captured by tMDL is preserved across evolutionary time. In this sense, tMDL tracks regulatory demand in a manner that is broadly preserved across gene ages. Second, the magnitude of this regulatory content differs systematically by evolutionary age: genes of intermediate evolutionary age consistently exhibit the highest cCRE counts across tMDL bins. Thus, even among genes with comparable tMDL, regulatory architectures vary with evolutionary history. Together with the tau-stratified analysis in Figure 6B, we have revised the text to more clearly highlight the biological implications suggested by this mid-age enrichment. One plausible interpretation is that recently evolved genes have not yet accumulated extensive regulatory architectures, whereas very ancient genes achieve comparable expression-pattern complexity using fewer regulatory elements, consistent with more efficient regulatory implementations over long evolutionary timescales. While this age-dependent pattern was present in the original analyses, the revised figure and accompanying text make this relationship more explicit.

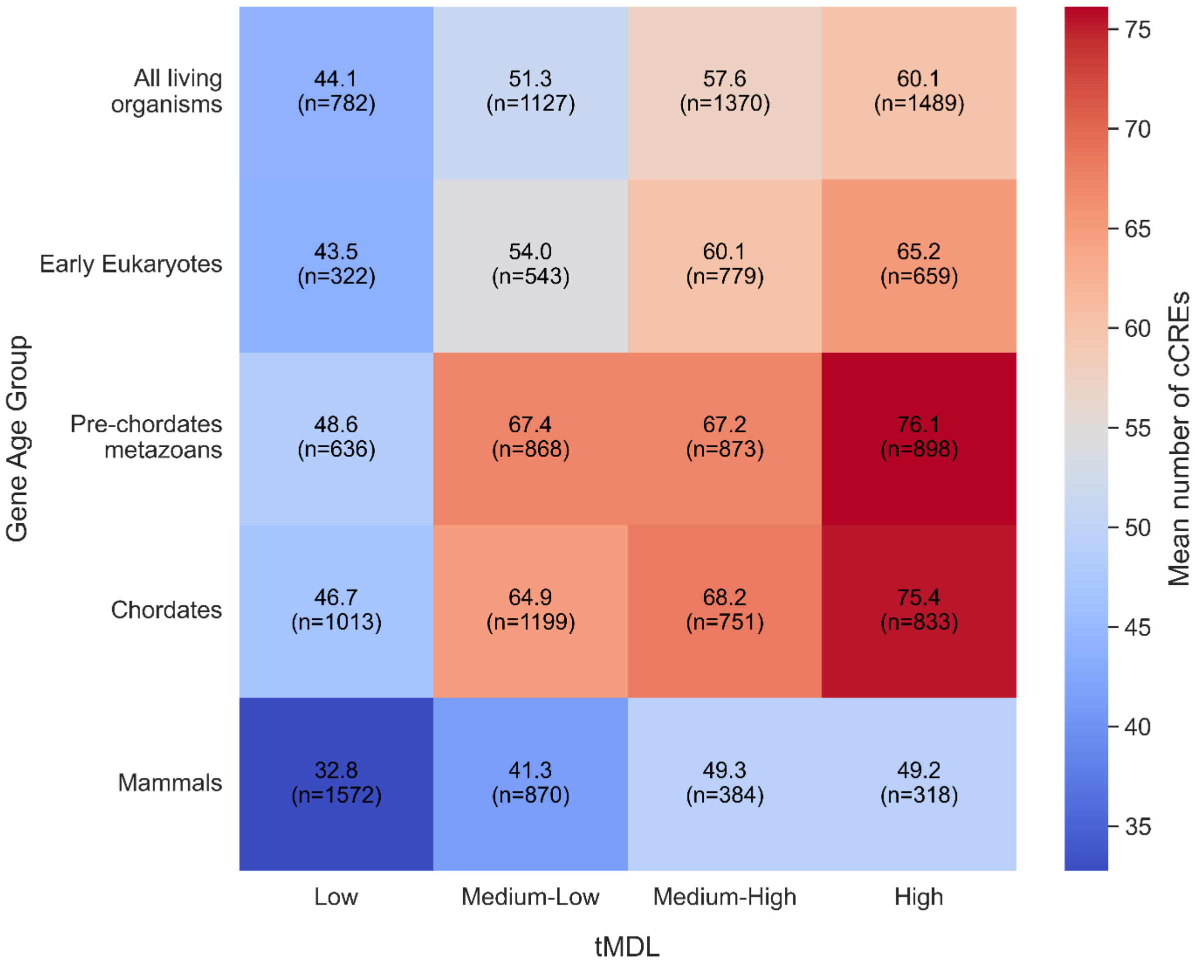

Response Figure 10. Revised analysis of regulatory architecture across evolutionary gene age and tMDL. (Figure 6C in the revised manuscript) Heatmap showing the mean number of linked cCREs for genes stratified by evolutionary age (y-axis; phylostrata) and binned by tMDL (x-axis). This representation replaces the previous slope-based analysis

Motivated by the reviewer's suggestion regarding transcription factors (TFs), we further explored whether TFs exhibit distinct age-dependent behavior. We find that TFs differ from the full gene set in a manner broadly consistent with the reviewer's intuition: older TFs tend to be broadly expressed, TFs of intermediate age are more cell-type/tissue specific, and the most recent TFs exhibit intermediate specificity (Response Fig. 11A). In addition, TFs as a class are skewed toward younger evolutionary ages compared to all genes (Response Fig. 11B). Developmental TFs, in particular, tend to originate at intermediate evolutionary stages, beginning in early metazoans and expanding during later chordate evolution, consistent with the emergence and elaboration of tissue differentiation programs.

Using the four-category framework suggested by the reviewer in a later comment, we further find that TFs are significantly enriched among genes with intermediate tau but low tMDL

(odds ratio = 2.3, Fisher's exact P = 3×10^{-42}) (Response Fig. 11C). This enrichment suggests that TFs often achieve cell-type specificity with comparatively efficient expression-pattern complexity, consistent with their high-impact regulatory roles across multiple cellular contexts.

We next examined TF regulatory footprint, defined here as the number of target genes per TF, across evolutionary age groups. Younger TFs (late vertebrate lineages) show a modestly larger footprint than ancient TFs (premetazoan lineages) (p-value ≈ 0.01), aligning with the reviewer's expectation (Response Fig. 11D). However, this increase in footprint size is not accompanied by a corresponding increase in tMDL (Response Fig. 11E). This indicates that a TF's regulatory footprint is likely decoupled from the regulatory demand required to encode the TF's own expression pattern. In other words, while younger TFs may act on more targets, this does not imply greater expression-pattern complexity of the TF itself.

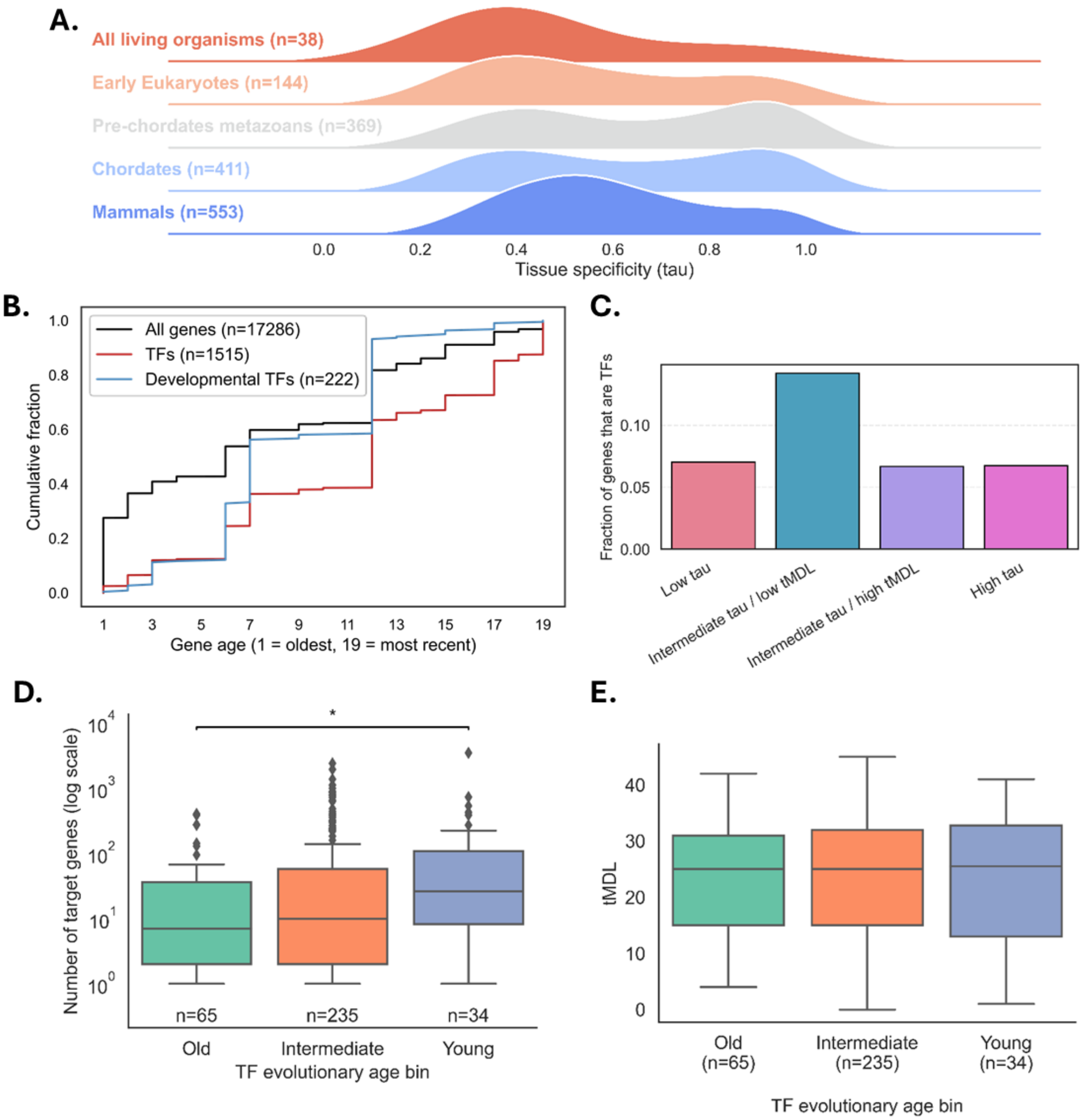

Response Figure 11. Evolutionary and regulatory characteristics of TFs. (A) Smoothed density plots showing the distribution of tissue specificity (tau) for TFs stratified by evolutionary age (grouped phylostrata). **(B)** Empirical cumulative distribution functions (ECDFs) of evolutionary age for all protein-coding genes (black), TFs (red), and developmental TFs (blue), with 1 representing the oldest and 19 the most recent lineages. **(C)** Fraction of genes that are TFs across four specificity and complexity regimes. Gene groups were defined by tissue specificity (tau; low ≤ 0.4, high ≥ 0.8), with the intermediate tau group further subdivided by median tMDL. **(D)** Distribution of TF regulatory footprints (number of target genes) across three evolutionary age bins (Old, Intermediate, Young). Footprints are shown on a log scale; p-values represent Bonferroni-corrected Mann-Whitney U tests. **(E)** Distribution of TF tMDL across the same evolutionary age bins.

Together, these analyses reinforce a central conclusion of the gene-age section: tissue specificity and regulatory architecture vary systematically with evolutionary age. Intermediate-age genes tend to rely on larger cCRE repertoires to achieve complex expression patterns, whereas older genes exhibit more compact regulatory implementations, consistent with greater regulatory efficiency. While the TF-focused analyses suggest additional interesting structure, they do not alter the core conclusions of the study and point instead to promising directions for future work.

These revisions are reflected throughout the revised gene-age section of the main manuscript and in the updated Figure 6C. The additional TF-focused analyses described above were performed in response to the reviewer's suggestion and are provided here for the reviewer's reference (Response Fig. 11).

Additional points

1. The study applies analyses to two datasets—one tissue-based and one cell-type-based—but it is not always clear which dataset is used in each analysis and figure (e.g., Fig. 2A, 2C).

Thank you for this constructive comment. We agree that clearly distinguishing between tissue-level and cell-type-level analyses is important, and your feedback highlighted places where this distinction was not sufficiently clear. To address this, we revised our descriptions throughout the results section and figure legends (including Fig. 2A and 2C) to ensure that the data source for the tau index is explicitly stated in each context. In addition, we have clarified in the Methods (lines 822-823) when tissue-level or cell-type-level data are used for the tau index and the rationale underlying these choices. We appreciate this comment, which improved the clarity of the manuscript.

2. The tau score treats each tissue independently when measuring tissue specificity. A more systematic approach would be to construct a hierarchical structure, recognizing that some tissues are more closely related to each other than to others. For example, two genes with similar tau scores may have very different implications if one is expressed in closely related cell types and the other in distantly related ones. While the author acknowledges this concern in the manuscript, they do not provide an optimized approach to address it and still rely on tau to measure tissue or cell-type specificity.

This comment raises an important conceptual point. The limitation described, namely that tau treats tissues or cell types independently and ignores their hierarchical relationships, is precisely what motivated us to develop the tree-aware MDL (tMDL). Rather than modifying

tau itself, we address this limitation by introducing tMDL as a hierarchy-aware measure that captures the complexity of expression patterns across cell types. As shown throughout the manuscript (e.g., Fig. 2C), tMDL resolves cases where genes have similar cell-type specificity (tau values) but markedly different regulatory demands.

We retain tau as a complementary measure because it provides a well-established, intuitive summary of expression breadth that facilitates comparison with prior studies, while tMDL captures our new, orthogonal dimension of regulatory complexity that tau is not designed to encode. Used together, the two measures disentangle expression-specificity from hierarchy-aware regulatory demand.

3. Although the authors state that the tau score is the most robust metric for measuring tissue specificity of gene expression, tissue specificity is a complex question—particularly since it is the central focus of this study—and may require more careful consideration. Is a single, simple metric sufficient to capture such complexity? Can genes with very low expression lead to misleading tau scores (e.g., a scatter plot of gene expression vs. tau)? How well does it perform across datasets with different numbers of cell types, varying profiling depths (i.e., data sparsity), and diverse count distributions? Could different normalization or scaling methods affect the robustness of tau? Might complementing tau with additional metrics provide more informative insights? It would also be useful to examine how tau correlates with other measures on the same datasets and whether they identify the same sets of cell-type-specific genes. All these questions require careful benchmarking.

We agree that tissue and cell-type specificity are inherently complex properties and warrant careful consideration, particularly given their central role in this study. We do not claim that a single scalar metric can fully capture all aspects of expression specificity; rather, tau is used here as a well-established and robust summary measure of expression breadth and variation that has been extensively benchmarked in prior work.

In particular, a comprehensive evaluation of tissue-specificity metrics demonstrated that tau performs robustly across tissue subsampling, normalization schemes, and cross-species analyses, and consistently ranks among the most stable measures for identifying tissue-specific genes (Kryuchkova-Mostacci & Robinson-Rechavi, 2017). Additionally, in that benchmarking study, the Gini index emerged as one of the closest alternative measures to tau, showing highly similar behavior across datasets and conditions. Consistent with this, we directly compared tau and the Gini index in our datasets and observed a very strong agreement between the two metrics (Spearman $\rho = 0.98$, Response Fig. 11; Supplementary Fig. 1B), indicating that tau captures a core notion of expression specificity that is also recovered by other measures.

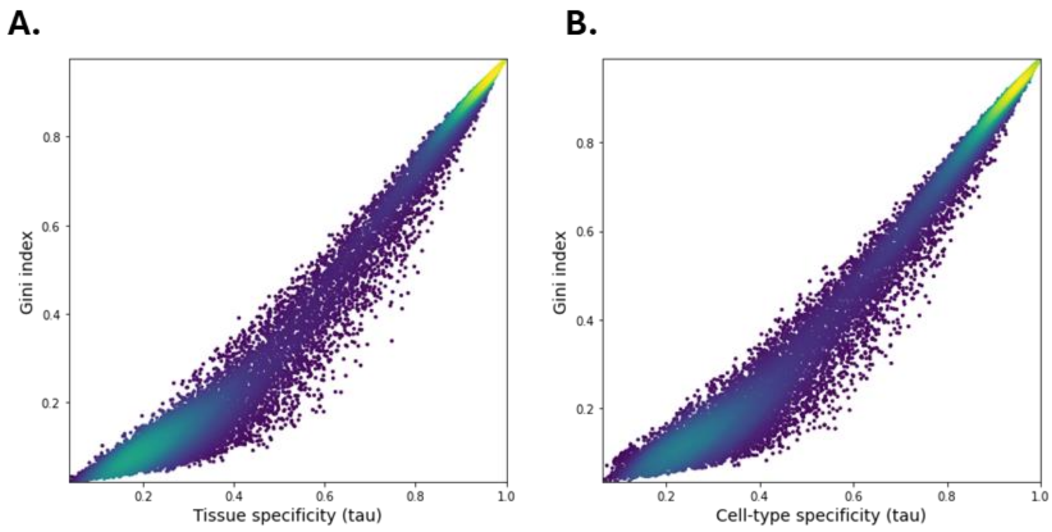

Response Figure 12. Agreement between tau and the Gini index as measures of tissue specificity (Supplementary Fig. 1B in the revised manuscript). Scatterplots showing the relationship between tau and the Gini index across genes. **(A)** Tissue-level comparison (Spearman's ρ = 0.98). **(B)** Cell type–level comparison (Spearman's ρ = 0.98).

We further examined whether lowly expressed genes or expression thresholding could lead to misleading tau values. As described in the Methods, genes with extremely low expression (nTPM<1 at a given tissue) are filtered prior to analysis to reduce noise. In addition, we explicitly tested the robustness of tau to different minimum expression thresholds, including no threshold and a more stringent cutoff (nTPM<5) (Response Fig. 13). Reassuringly, tau values remain highly concordant across thresholds, with near-perfect correlations. Increasing the threshold predictably shifts tau higher for more tissue-specific genes by removing weak expression in additional tissues, whereas including very low expression modestly decreases tau by distributing expression across more tissues. Overall, these results indicate that tau is robust to reasonable choices of expression thresholds and is not driven by low-level background expression.

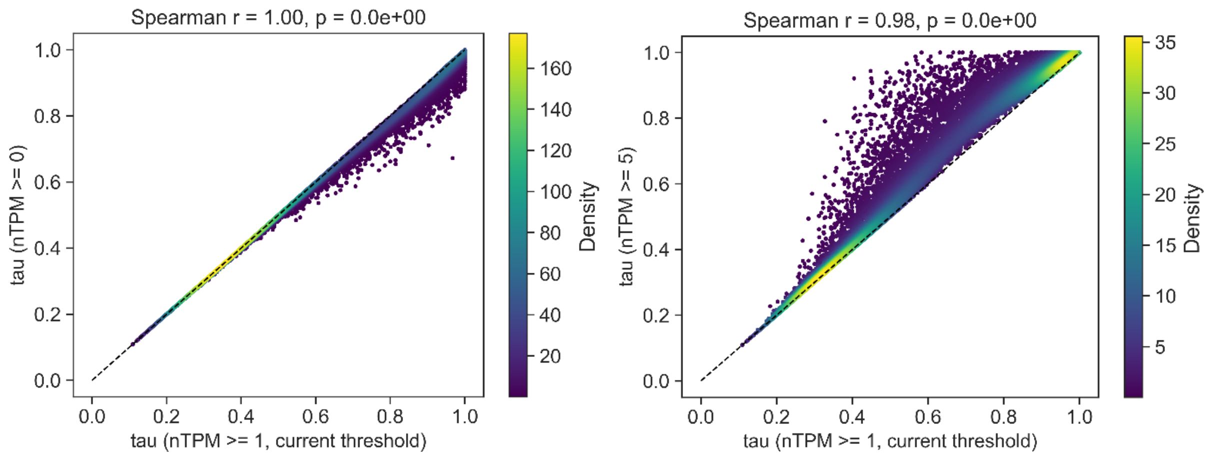

Response Figure 13. Robustness of tau to expression thresholding. Scatterplots comparing tau values computed using the primary threshold (nTPM > 1; x-axis) with values computed using alternative thresholds (y-axis). Points are colored by local density. **(A)** Comparison to no threshold (nTPM > 0; Spearman's ρ = 1.00). **(B)** Comparison to a stringent threshold (nTPM > 5; Spearman's ρ = 0.98).

The robustness of tau is further supported by its consistent behavior across datasets with markedly different numbers of cell types, profiling depths, sparsity levels, and normalization schemes, including bulk human tissue data, single-cell cell-type data, and cross-species analyses in mouse and Drosophila, as shown in the additional analyses presented in this response.

Finally, rather than relying on tau alone as one single metric, a central contribution of this work is to complement expression breadth with a hierarchy-aware measure of regulatory complexity. Together, tau and tMDL provide a more informative and nuanced characterization of gene expression programs than either metric alone.

The tau-Gini comparison is included in Supplementary Fig. 1 (lines 123-125), while analyses involving alternative expression thresholds for tau are reported only in this response.

4. Some cutoff selections appear arbitrary and require further justification. For example, the author uses 0.5 as a rough cutoff—genes with tau < 0.5 are considered broadly expressed across tissues, while genes with tau > 0.5 are thought to have progressively restricted expression. However, from Fig. 1A and S1B, there does not appear to be a clear cutoff at 0.5 that separates genes into distinct expression patterns. Genes with tau = 0.6 or even 0.7 can still be expressed across a broad range of tissues, making the choice of 0.5 questionable. Therefore, in Fig. 1C–E, dividing genes into low, tau = 0.5, and high groups is not convincing. Another unclear example is the division of genes into two groups—expressed in >90% versus <90% of cell types—as shown in Fig. 4A. Therefore, in Fig. 1C–E, dividing genes into low, tau = 0.5, and high groups is not convincing.

We thank the reviewer for this careful observation and agree that avoiding arbitrary cutoffs is important. We appreciate the opportunity to clarify how thresholds are used across the analysis and to strengthen the manuscript where additional justification was needed.

Regarding Fig. 1C-E, genes are not divided into discrete groups using fixed tau cutoffs. Instead, for each analysis we rank all genes by their tau value and examine enrichment patterns along this continuous ordering. Signals labeled as “low tau” and “high tau” correspond to genes drawn from the lower and upper ends of the tau ranking, respectively. For the “intermediate” analysis, genes are ranked by their tau values’ proximity to tau = 0.5, rather than being selected using a threshold.

Beyond being the mathematical midpoint of the tau scale (which ranges from 0 to 1), we chose tau ≈ 0.5 as a reference mid-point because, as shown empirically in Fig. 1A, genes with tau below ~0.5 are almost all expressed across ≥90% of tissues or cell types, whereas above this value the population begins to diversify, with increasing numbers of genes expressed in fewer tissues. Thus, tau ≈ 0.5 appears to naturally serve as a transition region for ordering genes along the tau continuum, rather than as a categorical cutoff. We have revised the text and figure legends to better clarify this analysis.

For the second example raised (Fig. 4A), we agree that the division of genes into those expressed in >90% versus <90% of cell types involves an explicit threshold choice. Because some thresholding is unavoidable in this context, we performed additional analyses using alternative thresholds. Reassuringly, the observed trends remain consistent across a range of cutoff values indicating that the conclusions do not depend on the specific threshold selected (Response Fig. 4; Supplementary Fig. 9).

These additional threshold sensitivity analyses are now included in the Supplementary Information (Supplementary Fig. 9; lines 465-468).

A. Ubiquitous genes = expressed in >**95%** cell types

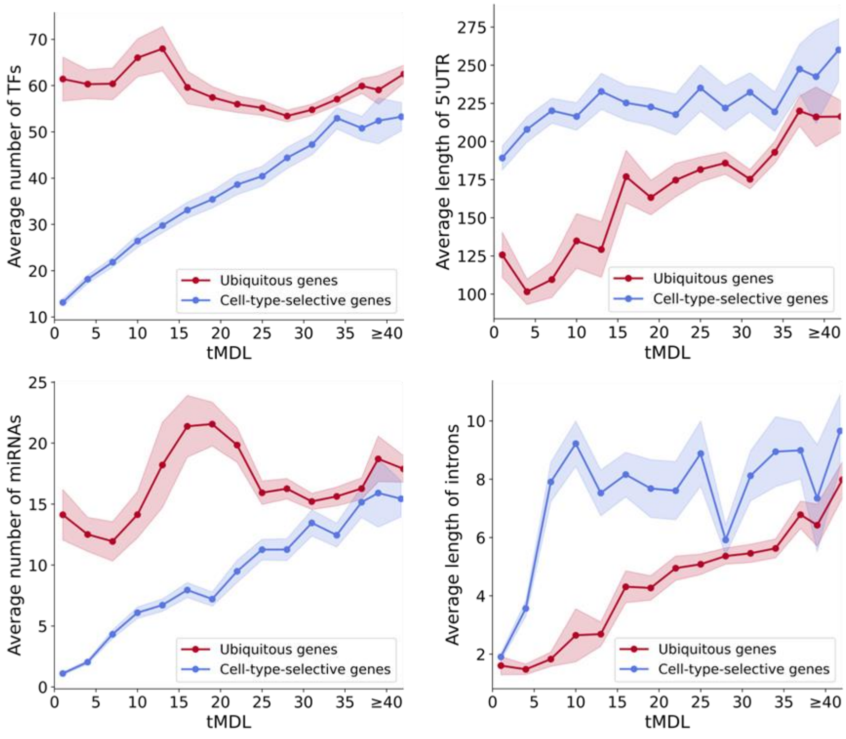

B. Ubiquitous genes = expressed in >**90%** cell types

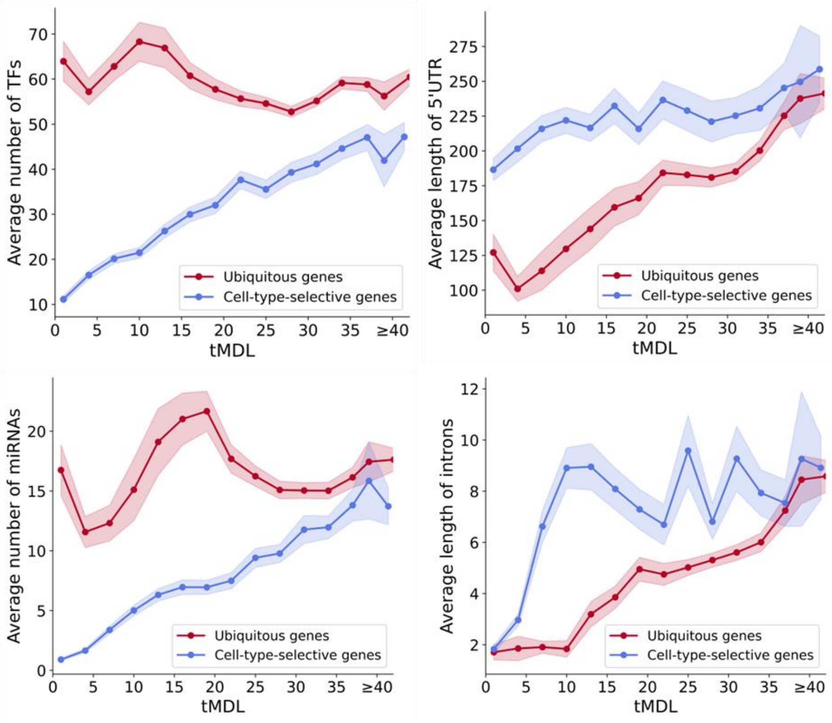

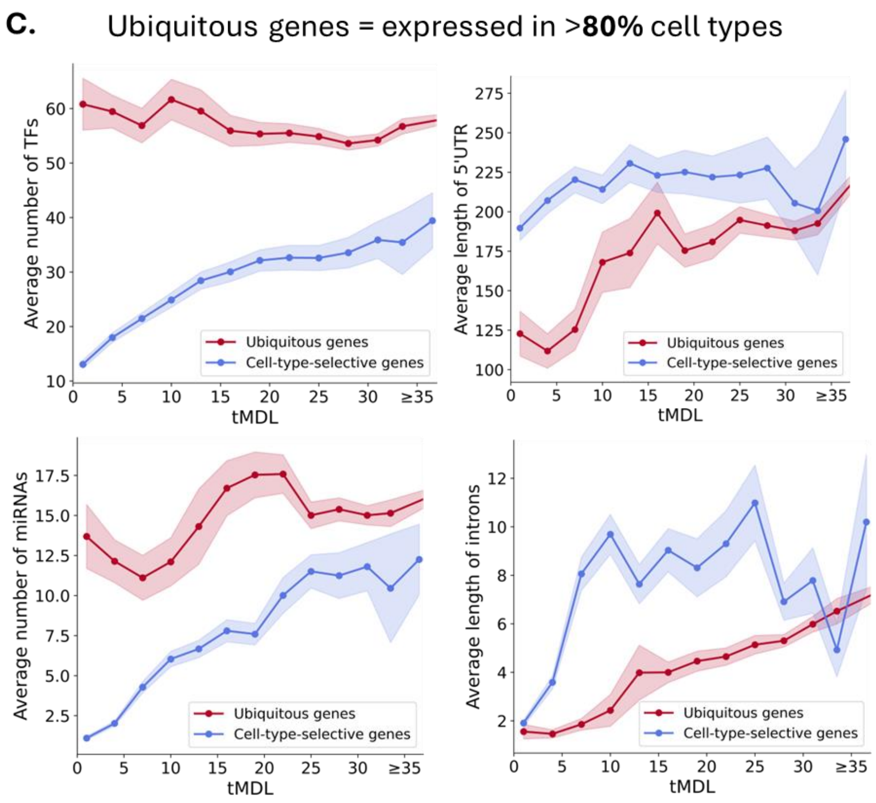

Response Figure 4. Robustness of knob- and switch-like regulatory regimes to the definition of ubiquitous expression. (Supplementary Fig. 9 in the revised manuscript) *Repeated from the analysis presented in response to Reviewer 3 (page 11).* Mean regulatory feature values plotted across ranked tMDL for ubiquitous (red) and cell-type-selective (blue) genes using alternative thresholds to define ubiquitous expression. (A) ≥95% of tissues, (B) ≥90% of tissues (used in the main text), and (C) ≥80% of tissues. Features shown: number of TFs, number of miRNAs, 5' UTR length, and intron length.

5. The relationship between tau and tMDL is not surprising given how they are calculated. As shown in Fig. 2B–C, genes with low or high tau scores have relatively uniform expression across cell types, while genes with intermediate tau scores show more heterogeneous expression, which explains their higher tMDL. A more interesting approach for subsequent analysis would be to split genes into four groups: low tau; intermediate tau with low tMDL; intermediate tau with high tMDL; and high tau.

While the relationship between tau and tMDL may be intuitively expected, we believe it is nonetheless an important and non-trivial result. The observed non-monotonic (horseshoe-shaped) pattern provides further support that tMDL behaves consistently with its intended role, capturing heirarchy of gene expression program, and heterogeneity in gene expression patterns, all in a tree-aware context rather than simply reflecting expression breadth. At the same time, we agree that taking this observation one step further, by distinguishing among genes with similar tau values, offers a particularly informative perspective.

To address this notion directly, we implemented a stratified analysis that separates intermediate-tau genes based on tMDL, as suggested. Rather than using fixed thresholds, which would be arbitrary, we applied a rank-based approach: we selected the middle 30% of genes ranked by tau and, within this group, compared genes in the top versus bottom 20% of tMDL values. Across all examined regulatory features, intermediate-tau genes with high tMDL consistently exhibited significantly greater regulatory complexity than those with low tMDL (Response Fig. 13A; Supplementary Fig 5A).

Importantly, these differences persist even when restricting the analysis to a much narrower tau window (i.e., the central 10% of tau-ranked genes), indicating that the observed effects are not driven by residual variation in tau but instead reflect genuine heterogeneity in regulatory architecture (Response Fig. 13B; Supplementary Fig 5B). We have incorporated the CRE-based stratification of intermediate-tau genes into the main Figure 2 (Fig. 2D), while analogous analyses for additional regulatory features are presented in the Supplementary Figures (Supplementary Fig. 5) (lines 285-292). This suggestion helped strengthen and clarify the interpretation of this section.

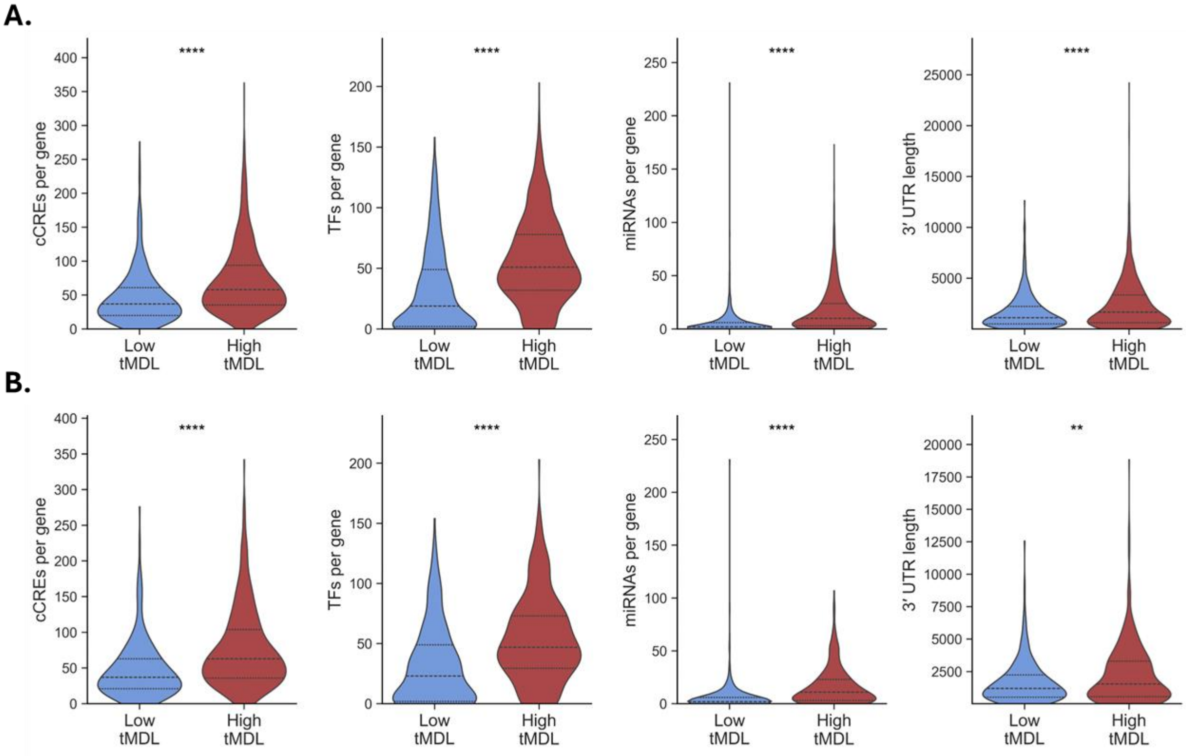

Response Figure 14. Regulatory feature differences between low- and high-tMDL genes at matched tissue specificity. (Supplementary Fig. 5 in the revised manuscript). Genes with intermediate tau values were stratified into low- and high-tMDL groups (bottom and top 20%), and regulatory features were compared between groups. Violin plots show feature distributions; significance was assessed using two-sided Mann-Whitney U tests. **(A)** Intermediate tau defined as the middle 30% of genes by tau. **(B)** Intermediate tau defined

as the middle 10% of genes by tau. Features shown (left to right): number of cCREs, number of TFs, number of miRNAs, and 3' UTR length.

6. The tMDL calculation seems somewhat circular, as the cell-type tree is built based on gene expression similarity, and then genes correlated with this structure are further evaluated. In addition, the author could consider using alternative methods to benchmark tMDL, e.g., https://github.com/landau-lab/PATH.

We appreciate this comment and agree that the interpretability of tMDL depends on the robustness of the underlying cell-type hierarchy. As noted above in response to the reviewer's constructive major comment #3, we addressed this concern by recomputing tMDL using an independent, externally defined cell-type hierarchy derived from genome-wide single-cell ATAC-seq data, rather than one inferred from gene expression. Using this fixed external topology, we recomputed tMDL for the same set of genes and cell types.

Reassuringly, tMDL values computed using the external hierarchy were highly concordant with those obtained from the expression-derived tree, and all key downstream results were preserved. This indicates that our conclusions are not sensitive to the specific method used to construct the hierarchy and alleviates concerns about circularity. We appreciate this crucial point which is also reflected in the modified main text (lines 256-260) and in Supplementary Fig. 3.

7. When calculating tMDL, the methods indicate that expression levels across cell types for each gene were discretized into six bins. I am not sure how this approach handles genes with roughly uniform expression across cell types, such as DHX15.

This is a perceptive point. Genes with approximately uniform expression across cell types are handled naturally by the tMDL framework: when a gene occupies the same discretized expression bin across all cell types, no expression state transitions are required along the hierarchy, and the resulting tMDL is zero or near zero. This outcome is intentional and reflects the interpretation of tMDL as a measure of differential regulatory demand across the cell-type hierarchy. In practice, near-perfect uniform expression across all cell types is not common, and many broadly expressed genes therefore have low but non-zero tMDL values.

Accordingly, broadly expressed genes such as DHX15 receive low tMDL values, distinguishing them from genes with heterogeneous expression across related or distant cell types. In the case of DHX15, the tMDL is low (tMDL = 19; for reference see Fig. 2C) rather than zero, reflecting modest but measurable variation in expression levels across cell types despite its broad housekeeping-like expression.

To make this explicit, we have now highlighted all four example genes from Figure 1B, including DHX15, in Figure 2C, which plots tau against tMDL, allowing their relative positions in both expression breadth and expression-pattern complexity to be visualized directly.

8. The second half of the paper conducts multiple systematic analyses to investigate regulatory features related to tissue specificity and regulatory demand, which are way more interesting. However, none of these analyses go very deep, either in terms of methodology or in providing additional validation or benchmarking, which makes the results less convincing. I would suggest that the author streamline the first half of the manuscript—quickly introducing the concepts of cell-type specificity and regulatory demand (tMDL)—and instead place greater emphasis on how regulatory features correlate with specificity.

We appreciate this perspective and agree that deeper mechanistic dissection of individual regulatory features is an important and valuable direction. The primary aim of this study, however, is to introduce a novel, systematic, and quantitative framework for linking gene expression specificity to regulatory demand across multiple layers of regulation, rather than to provide an in-depth mechanistic analysis of any single feature.

To address this concern, we strengthened the manuscript by adding multiple validation analyses that increase confidence in the observed relationships, many of which were prompted by the reviewer's constructive comments. Specifically, we now show that the main findings are preserved across species (including mouse and *Drosophila*), are robust to the use of alternative expression datasets, and are overall recapitulated across multiple independent CRE annotation databases. Together, these additions increase confidence that the observed correlations reflect stable and biologically meaningful patterns.

Regarding manuscript structure, while we agree that the analyses linking regulatory features to specificity are a central contribution, we believe that a clear and careful introduction of cell-type specificity and tMDL is essential, as these concepts represent novel methodological advances that underlie all subsequent results. Nevertheless, in response to this comment, we have aimed to refine and improve the flow of the paper.

Overall, we believe the revised manuscript better balances conceptual clarity with a broad, integrative view of regulatory architecture, while naturally motivating deeper mechanistic studies as important directions for future work.

9. Can the authors provide (sorry if we missed this) clean lists of human gene names/IDs along w/ associated T, MDL and tMDL values? Ideally, e.g. if you follow some of the suggestions above, it would also be reportable as a single clean file for mouse genes as well.

The field would benefit from such a table that avoids the dichotomization of housekeeping vs. developmental genes and instead presents a more nuanced, continuous view.

We appreciate this suggestion and are glad the reviewer sees value in such a resource. We have included a clean table listing human genes together with their associated tau, tMDL, and the various regulatory features as Supplementary Table 1, and provide a corresponding table for mouse genes as well (Supplementary Table 2). We hope this resource will be useful to others and facilitate further analyses that treat these measures as continuous rather than categorical variables.

REVIEWERS' COMMENTS

Reviewer #1 (Remarks to the Author):

The authors have addressed my concerns. However, code for the new analysis performed in the revisions are missing.

We thank the reviewer for this comment and are pleased that the previous concerns have been addressed. The analyses added in the revision rely on the same tMDL framework provided in Supplementary Code 1. The revisions involved applying this framework to alternative datasets and cell-type hierarchies to assess robustness, rather than introducing new computational methods or modifying the underlying algorithm.

To further facilitate reproducibility and ease of use, we have expanded Supplementary Code 1 to include construction of the expression-based cell-type similarity tree used throughout the manuscript, thereby providing a complete end-to-end implementation of the tMDL framework. While the previous version included expression discretization and application of Fitch's parsimony algorithm, the addition of tree construction now enables users to compute tMDL scores directly from an input expression matrix. Users may provide their own expression matrix or use the publicly available Human Protein Atlas matrix analyzed in this study, whose source is explicitly specified in the Methods and Supplementary Code.

Other analyses in the manuscript (e.g., tau calculation, correlation analyses, binning procedures, enrichment analyses, and statistical testing) involve established analytical approaches fully described in the Methods section and applied to publicly available datasets explicitly listed therein. With the expanded tMDL implementation, clearly specified data sources, and provision of derived gene-level tables, the analyses presented in this study are reproducible.

Reviewer #2 (Remarks to the Author):

I co-reviewed this manuscript with one of the reviewers who provided the listed reports. This is part of the Nature Communications initiative to facilitate training in peer review and to provide appropriate recognition for Early Career Researchers who co-review manuscripts.

Reviewer #2 (Remarks on code availability):

The code submitted in Supplementary Code 1 is only for tMDL score calculation (with binned gene expression values). While the Methods section explain their analysis in detail, the code

for other analyses and plotting is severely lacking. In its current state, this data is not reproducible.

We appreciate the reviewer's concern regarding reproducibility. The central methodological contribution of this study is the tMDL framework, which underlies the primary analyses presented. Additional analyses in the manuscript (e.g., tau calculation, correlation analyses, binning procedures, enrichment analyses, and statistical testing) rely on established analytical approaches that are fully described in the Methods section and applied to publicly available datasets whose sources are explicitly specified therein. These analyses do not involve additional custom computational methods beyond the tMDL framework.

To facilitate reproducibility of the core framework itself, we have expanded Supplementary Code 1 to provide a complete end-to-end implementation of the tMDL calculation, including construction of the expression-based cell-type similarity tree in addition to expression discretization and application of Fitch's parsimony algorithm. The code enables users to compute tMDL scores directly from an input expression matrix. Users may supply their own data or download the publicly available Human Protein Atlas dataset employed in this study, whose source is explicitly specified in the Methods and Supplementary Code.

To further ensure full transparency and reproducibility, we provide complete gene-level annotations and derived features, including tau, tMDL, and various regulatory features, in Supplementary Data 1 and 2 (for human and mouse). These tables contain the processed values underlying all figures and statistical analyses and allow independent regeneration of the reported results.

With the expanded tMDL implementation, clearly specified data sources, and provision of derived gene-level tables, the analyses presented in this study are reproducible.

Reviewer #3 (Remarks to the Author):

The authors have satisfyingly addressed all my comments and suggestions. This is an exciting piece of work.

We thank the reviewer for their thoughtful evaluation and encouraging feedback.

Reviewer #4 (Remarks to the Author):

The authors have done a terrific job, in our view, of addressing the comments of us and the other reviewers, and we wholeheartedly recommend publication.

We sincerely thank the reviewer for their positive assessment and recommendation.
